# Single-cell characterization of neovascularization using hiPSC-derived endothelial cells in a 3D microenvironment

**DOI:** 10.1016/j.stemcr.2023.08.008

**Published:** 2023-09-14

**Authors:** Simon Rosowski, Caroline Remmert, Maren Marder, Misao Akishiba, Judith Bushe, Annette Feuchtinger, Alina Platen, Siegfried Ussar, Fabian Theis, Sandra Wiedenmann, Matthias Meier

**Affiliations:** 1 Helmholtz Pioneer Campus, Helmholtz Zentrum München, Neuherberg, Germany; 2 Research Unit Analytical Pathology, Helmholtz München, 85764 Neuherberg, Germany; 3 Institute for Diabetes and Obesity, Helmholtz Diabetes Center, Helmholtz Zentrum München, Neuherberg, Germany; 4 Institute of Computational Biology, Helmholtz Zentrum München, Neuherberg, Germany; 5 German Center for Diabetes Research (DZD), 85764 Neuherberg, Germany; 6 Department of Mathematics, Technical University of Munich, 85748 Garching bei München, Germany; 7 University Leipzig, Center for Biotechnology and Biomedicine, Institute of Biochemistry, Leipzig, Germany

**Keywords:** single-cell mRNA sequencing, stem cell-derived endothelial cells, culturing technologies, neovascularization, inferred EC-pericyte interactions, microfluidic ligand assay

## Abstract

The formation of vascular structures is fundamental for *in vitro* tissue engineering. Vascularization can enable the nutrient supply within larger structures and increase transplantation efficiency. We differentiated human induced pluripotent stem cells toward endothelial cells in 3D suspension culture. To investigate *in vitro* neovascularization and various 3D microenvironmental approaches, we designed a comprehensive single-cell transcriptomic study. Time-resolved single-cell transcriptomics of the endothelial and co-evolving mural cells gave insights into cell type development, stability, and plasticity. Transfer to a 3D hydrogel microenvironment induced neovascularization and facilitated tracing of migrating, coalescing, and tubulogenic endothelial cell states. During maturation, we monitored two pericyte subtypes evolving mural cells. Profiling cell-cell interactions between pericytes and endothelial cells revealed angiogenic signals during tubulogenesis. *In silico* discovered ligands were tested for their capability to attract endothelial cells. Our data, analyses, and results provide an *in vitro* roadmap to guide vascularization in future tissue engineering.

## Highlights

• In this study, we engineered stem cell-derived vessels in a 3D cell culture
• Time-resolved single-cell transcriptomes reveal similarities to human embryogenesis
• Discovery of cell communication between pericytes and ECs during tubulogenesis

## Graphical abstract

**Figure:**
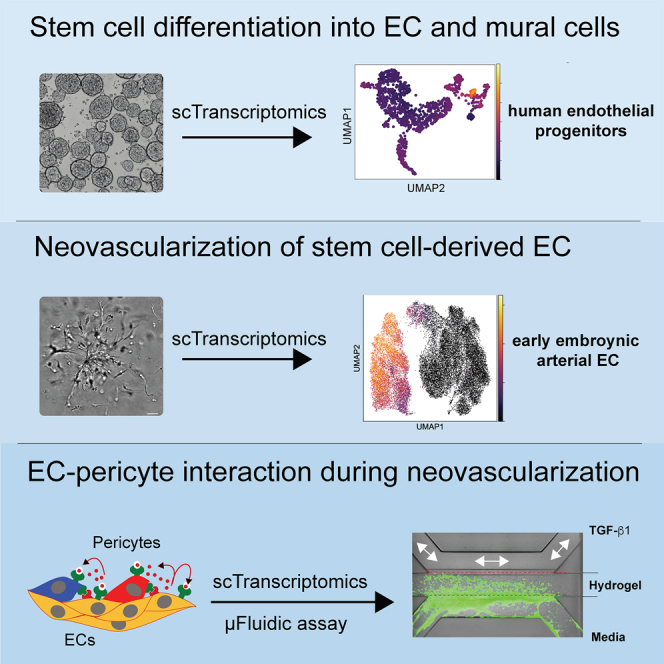

## Teaser

In this article, Meier and colleagues engineered stem cell-derived vessels comprising co-evolving endothelial cells and pericytes in a 3D hydrogel environment. With single-cell transcriptomes and a microfluidic migration assay, the authors characterized the transcriptional changes and cell communication signals between pericytes and endothelial cells during the process of neovascularization.

## Introduction

Endothelial cells (ECs) form the inner luminal epithelium of vascular structures, including arteries, veins, and lymphatic vessels (Ricard et al., 2021). Apart from core nutrient and oxygen transport functions, the vascular system enables immune cell trafficking, vasomotor tone, and wound healing (Chatterjee, 2021). To fulfill the different homeostatic functions, ECs exhibit a high degree of plasticity to form differently sized fenestrae and branched vessel structures, recruit organ-specific scaffold cells, or remodel the extracellular matrix composition of the vascular bed (Dejana et al., 2017; Holm et al., 2018). The developmental factors determining EC formation, maturation, and specification are not fully revealed yet.

*In vitro* cell culture systems with patient and stem cell-derived ECs have become indispensable for vascular research to overcome the paucity of longitudinal studies in patients, reducing the biological complexity. In particular, stem cell-derived ECs are of interest because they provide access to the early development stage of vascular structures involving cell type formation and specification (Nguyen et al., 2021). *In vivo*, ECs evolve from mesoderm-derived progenitors (angioblasts) in response to FGF2 and VEGFA signals from the adjacent visceral endoderm (Poole et al., 2001). Stem cell differentiation protocols recapitulate *in vivo* development by first inducing mesodermal progenitor cells, with BMP4 and GSK3β inhibitors (Tan et al., 2013), and subsequently ECs, by adding VEGFA (Chen et al., 2011). Several variations of the differentiation protocols have been reported (Xu et al., 2019), where the differences between the resulting ECs remain unresolved. Stem cell-derived ECs do not show a direct correlation with any organ-specific ECs, and it remains unclear which developmental stage they reflect (Paik et al., 2020). To closely resemble the *in vivo* microenvironment in a dish, 3D stem cell culture formats were introduced, which enable stem cell-derived ECs to form tubes (Olmer et al., 2018). Moreover, stem cell-derived ECs can fully self-assemble into blood vessels in an organoid-like format, including mural cells, i.e., smooth muscle and pericytes lining the outer surface of the vessel endothelium (Wimmer et al., 2019). In all culture formats, mural cells co-evolve during EC differentiation (McCracken et al., 2020), which implies inherent strong cell-cell communication during differentiation and maturation.

During embryonic development, immature ECs coalesce and undergo tube formation to produce a vascular plexus (Noden, 1989), which further differentiates into the specific vessel types. Single-cell transcriptomic analysis of the *in vivo* neovascularization process was performed by extracting cells from laser-induced choroid lesions in mice (Rohlenova et al., 2020). The data depicted a highly heterogeneous process with multiple transcriptomic EC stages and types, including phalanx and tip cells, with associated distinctive metabolic profiles. Comparable single-cell transcriptomic analyses in a less complex *in vitro* microenvironment with stem cell-derived ECs is not available but would add fundamental knowledge on the formation of vascular structures.

In this study, we used single-cell transcriptomics to sequentially investigate the development and neovascularization of human induced pluripotent stem cells (hiPSCs) in an *in vitro* 3D microenvironment. In the first step, we used single-cell transcriptomics to explore the differentiation trajectory of co-evolving ECs and mural cells in a 3D suspension cell culture format. Comparison of single-cell transcriptomics of ECs, evolved from a monolayer and a 3D suspension culture, revealed differences in ECM gene expression and optimal differentiation parameters. In the second step, the single-cell transcriptomics approach was used to analyze neovascularization in the heterogeneous 3D suspension culture upon transfer into a hydrogel culture. Ligand-receptor links between ECs and subcellular pericyte populations were predicted from the single-cell transcriptomes during vessel maturation. To prove functional importance of the *in silico*-discovered ligands, we then tested for their potential to attract ECs in an *in vitro* migration assay. Our data and analysis provide the resources for future EC specification studies and tissue engineering approaches.

## Results

### Single-cell analysis of endothelial differentiation in a 3D suspension culture

**Figure 1:**
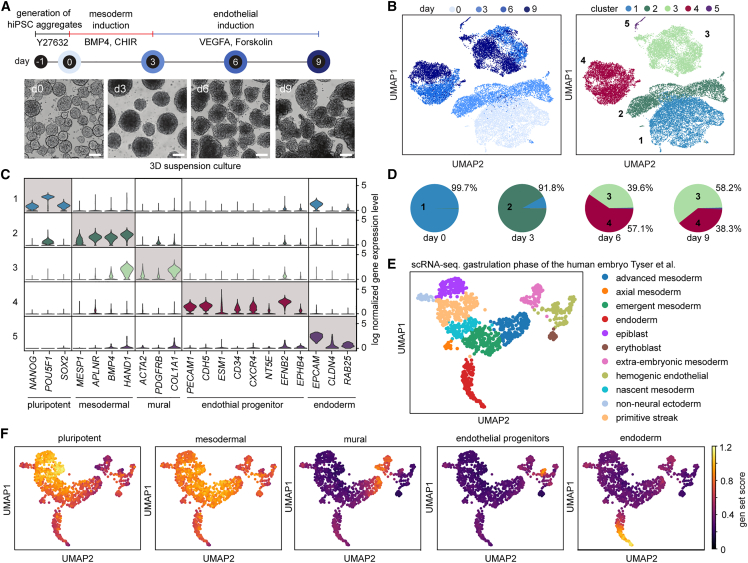
Single-cell transcriptomics reveals the differentiation trajectory of hiPSCs into endothelial cells in 3D suspension culture (A) Schematic of the endothelial differentiation timeline with sampling points and chemical induction protocol. Bright-field images show representative 3D suspension cultures at the corresponding time point. Scale bar denotes 100 μm. (B) UMAP plot of the single-cell transcriptomes. Left: light to dark blue denotes the time points of sampling. Right: five unique cell clusters were identified during the endothelial differentiation: (1) pluripotent cells, (2) mesodermal cells, (3) mural cells, (4) endothelial progenitor cells, and (5) epithelial cells. (C) Violin plot shows the cluster expression levels of differentially expressed genes for the six cell clusters and the commonly used cell markers for cell-type assignment. (D) Cell-type distribution analysis along the differentiation trajectory. (E) Single-cell transcriptomic data with cell-type assignment of the human embryo at the gastrulation phase adopted from Tyser et al. (2021). (F) Gene set enrichment analysis of the top 300 DEGs of each cell cluster found in (B) within the cell clusters of the human embryo at the gastrulation phase shown in (E).

To investigate the differentiation of hiPSCs into ECs in a 3D cell culture format at the single-cell level, we adopted the chemical two-step induction protocol (Olmer et al., 2018; Patsch et al., 2015; Wimmer et al., 2019). Therefore, hiPSCs were differentiated toward the mesoderm germ layer, and EC development was induced in the second step (Figure 1A). In suspension, the 3D cell cultures were stable throughout the differentiation and grew from a diameter of 150 to 300 μm. While hiPSC-derived aggregates exhibited a uniform spheroidal shape, aggregates from day 4 showed a more prolate shape. On day 9, 33.2% of the cells expressed the endothelial marker CD31 (PECAM1), with a standard variation of 5.3% over three biological repeats and two patient cell lines (Figures S1A and S1B). To reconstruct EC development in the 3D suspension culture and define time-resolved cell composition, we performed single-cell mRNA sequencing (scRNA-seq) analysis on 22,192 cells (see Table S1). Upon dimensional reduction (McInnes et al., 2018) and Leiden clustering (Traag et al., 2018), the cells were assigned into five clusters. With the progression of the differentiation process, the recorded single-cell transcriptomes changed, as indicated by the time-dependent emergence of distinct cell clusters (Figure 1B). All cell clusters could be assigned to cell types by matching known mesodermal and endothelial developmental markers to the differentially expressed genes (DEGs) in the respective cluster (Figure 1C). The cell populations were assigned to pluripotent stem cells (cluster 1), mesodermal cells (cluster 2), mural cells (cluster 3), endothelial progenitor cells (cluster 4), and endoderm (cluster 5). At the start of differentiation (day 0), the cell population consisted of homogeneous undifferentiated hiPSCs, where over 96% of the cells expressed the pluripotency markers *OCT4*, *SOX2*, and *NANOG*. Cells assigned as mesodermal cells appeared on day 3 of differentiation and expressed markers for the lateral plate mesoderm, including *HAND1*, *MESP1*, and *APLNR.* Mural cells observed on day 6 of differentiation showed reduced *HAND1* expression level, while the smooth muscle marker *ACTA2*, pericyte marker *PDGFRB*, and mesenchymal marker *COL1A1* were consistently expressed. Only a small fraction (0.9%) of endoderm cells was observed (Figure 1D). Immunohistochemical staining of 3D suspension cell cultures at day 9 of differentiation with the markers PECAM1 and PDFGRB showed de-mixing of the two cell populations; however, there was no induction of vessel formation, supporting the endothelial progenitor cell state (Figure S2A). Evaluation of cell-cycle states showed that endothelial progenitor cells were entirely in the G1 phase, whereas approximately 50% of the mural cells were in G2 and S and thus proliferating (Figure S2B). This explains the increasing proportion of mural cells from days 6 to 9 in the 3D cell culture. To test the robustness of the differentiation approach at the single-cell level, we sequenced the cells from day 9 of two independent differentiation experiments. In both cases, a bimodal distribution of endothelial progenitor cells and mural cells was observed, with comparable distribution numbers (Figure S2C).

To further confirm the cluster assignments, we calculated a gene expression enrichment score of the top 300 DEGs of each cell cluster within a single-cell dataset of the human embryo at the gastrulation phase (Tyser et al., 2021) (Figures 1E and 1F). Expectedly, the DEGs of the pluripotent stem cells were highly enriched in human epiblast cells. The *in vitro*-differentiated mesodermal cell on day 3 correlated broadly with nascent, emergent, and advancing mesoderm cells of the human gastrulating embryo. Strikingly, within the top 300 DEGs of the endothelial progenitors, genes of the hemogenic ECs are significantly enriched. Gene enrichment factors for the co-evolving mural cells were highest with advancing mesoderm and extraembryonic mesoderm cells. In summary, the gene enrichment analysis showed that the occurring cell types of the *in vitro* differentiation resemble the cell types found during the mesodermal-to-hemogenic EC development of the human embryo during gastrulation.

### Time trajectory of endothelial development

**Figure 2:**
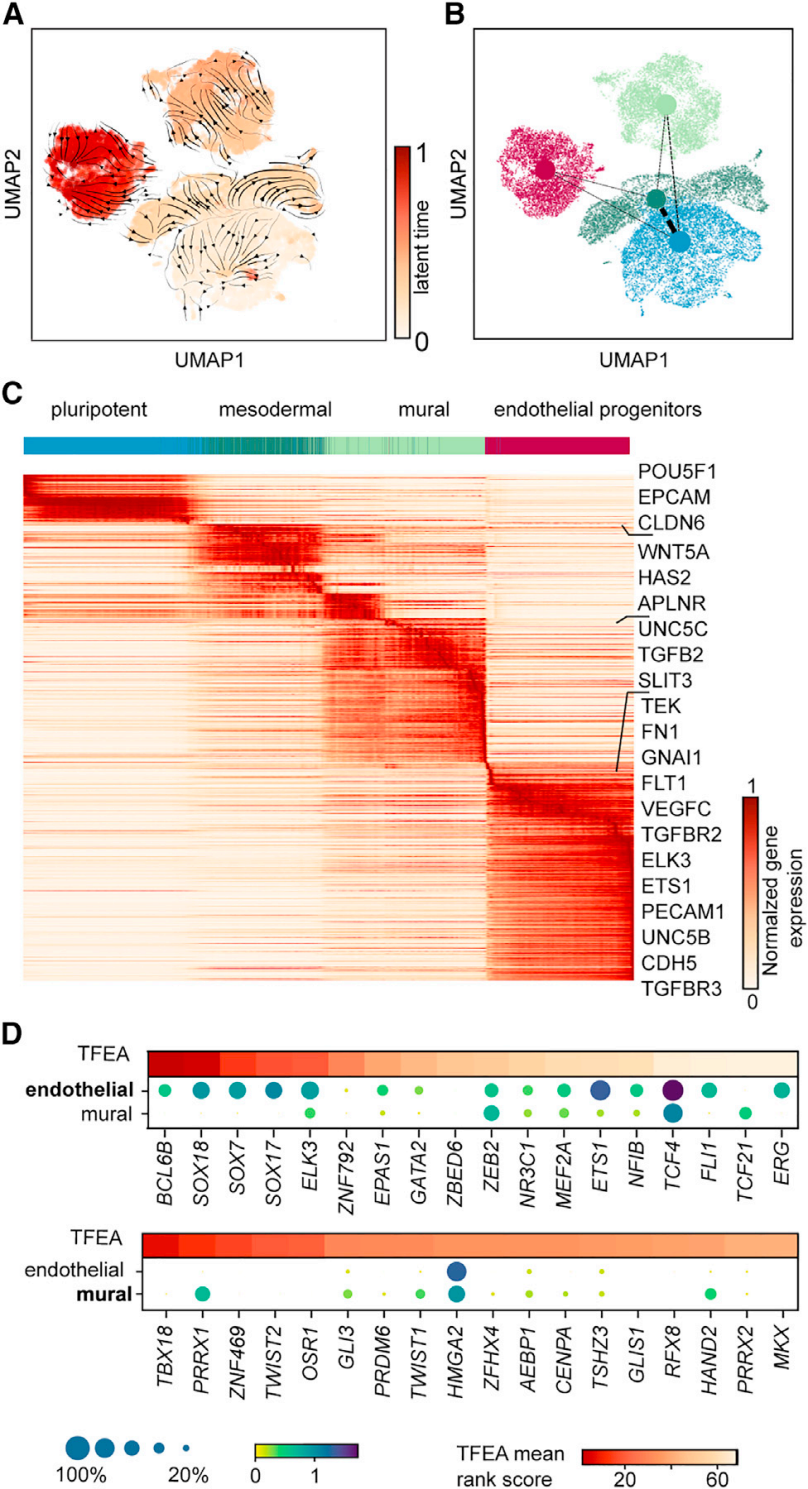
Transcriptomic dynamics predict the differentiation path for endothelial and mural cells (A) UMAP plot colored with the latent time calculated based on RNA velocity analysis. The velocity streamlines are given by the black arrows. (B) UMAP plot colored by annotated cell-type colors and with an overlay of PAGA connectivity. (C) Heatmap of the cluster-specific DDGs sorted according to their likelihood scores and latent time (see Table S4). (D) TFEA on the cluster-specific and unique DDGs for endothelial progenitor cells and mural cells. TFEA scores are represented in a color code, whereas the mean expression levels of the corresponding TFs are shown as dot plots. The color intensity and dot size denote the normalized cluster mean expression and the fraction of cells expressing the gene, respectively.

To resolve the time-dependent relationships of the cell clusters, we performed a dynamic RNA velocity analysis and identified the dynamic driver genes (DDGs) in endothelial differentiation. For this, we first calculated the latent time based on the balance of spliced and unspliced RNA transcripts within the single-cell transcriptomes (Figure 2A). The corresponding RNA velocity is weakly streamlined between the transition states of the cell clusters. Nevertheless, a partition-based graph abstraction (PAGA) analysis on the RNA velocity demonstrated the connectivity (Figure 2B) between the endothelial progenitor and mural cells and the mesodermal progenitors. Subsequently, we plotted the DDGs along the velocity latent time, where mural and endothelial progenitor cells were defined as end states, to trace the central genes for the development of the respective cell types in 3D (Figure 2C). Similar to the DEG analysis, cluster-specific DDGs were identified. The list of DDGs for each cell cluster is given in Table S4. The top DDGs for the mural cell progenitors were involved in cell migration, attraction, or repulsion (*UNC5C*, *SLIT3*, and *TGFB2*). For ECs, known developmental genes of vasculogenesis were upregulated, including the *VEGF* receptors (*KDR*, *FLT1*) and the interacting receptors *TIE1* and *TEK*. To infer the transcription factors (TFs) controlling the development of endothelial progenitor cells and mural cell progenitors, a TF enrichment analysis (TFEA) was performed on the DDGs (Figure 2D). The highest-ranked TFs for EC development were *BCL6B*, *ETS1*, *ELK3*, and *ERG*. All of them are reportedly associated with the process of early vasculogenesis with context-dependent function but integrate the VEGF and Notch signaling pathways. Notably, the extracted TFs are putatively responsible for the development of the two identified cell types, but not for neovascularization, due to the missing vessel organization within the 3D cell cultures at days 6 and 9 of differentiation. TFEA of the DDGs for the mural cell progenitors revealed *TBX18*, *CENPA*, and *HAND2* as the top regulatory TFs (Osterwalder et al., 2014). Expression of *TBX18* was not detected in the scRNA dataset; however, its transcriptional activity matched with its recently identified expression pattern in mouse pericytes and vascular smooth muscle cells of the retina, brain, heart, skeletal muscle, and adipose fat depots (Wu et al., 2013). It must be considered that the velocity analysis with spare time points has to be understood as transcriptional correlation rather than real dynamics. Nevertheless, the turn on and off of genes derived from the velocity analysis match with the expectation, for example, for the cell-cycle regulator *HMGA2*. In mural cells, *HMGA2* is in the turned-on state, whereas in endothelial progenitor cells it is in the turned-off state but still detectable.

### Comparison of endothelial differentiation in 3D versus 2D cell culture formats

In the next step, we sought to compare the *in vitro* development of ECs in 3D suspension culture with a previously performed stem cell differentiation approach with an adherent 2D cell culture on the single-cell level (McCracken et al., 2020). Chemical compounds for the endothelial induction protocol were the same; however, minor concentration differences of the individual compounds existed (Table S2).

**Figure 3:**
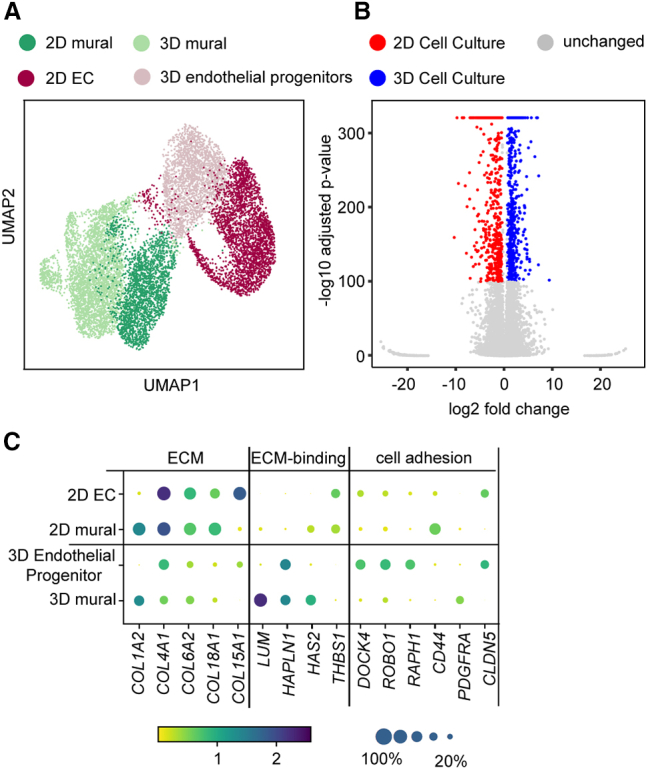
Comparison of EC differentiation in 2D and 3D cell culture formats (A) UMAP plot of single-cell transcriptomes of iPSC-derived endothelial cells differentiated on a 2D adhesion monolayer (5,267 cells) and as 3D suspension culture (6,492 cells). (B) Volcano plot representing genes differentially expressed between ECs differentiated in 2D and 3D cell cultures. (C) Dot plot of representative DEGs with assigned cellular functions and biological processes. The color intensity and dot size denote the normalized cluster mean expression and fraction of cells expressing the corresponding gene, respectively.

Single-cell transcriptomes of mural cells and ECs differentiated to day 9 in a 3D cell culture showed a different marker expression profile than those differentiated to day 8 in a 2D cell culture (Figure 3A). This led to the result of two separate Leiden clusters for the cell types cultured in 2D and 3D in the UMAP plot. To quantify the differences, we combined the two scRNA datasets. Of the 14,383 genes, 575 and 608 showed expression-level differences with a p value lower than 10^−100^ in the 2D and 3D cell cultures, respectively (Figure 3B). Dominantly, the expression patterns of the extracellular matrix (ECM) genes were distinct in different culture formats. Within the 2D cell culture format, ECs exhibited a strong collagen phenotype with high expression levels of basal lamina proteins, such as COL4A1/2, COL6A2, or COL18A1 (Figure 3C). In 3D cell culture, ECs expressed hyaluronic acid and the corresponding binding proteins. In contrast, endothelial progenitor cells within the 3D suspension culture upregulated cell-cell interaction and actin remodeling genes, such as the Wnt signaling proteins CLD5, DOCK4, and CTNNB1 and the Rap1 signaling proteins RAP1B, RAPGEF5, and RASIP1.

Within the 3D cell culture endothelial progenitor cells stop proliferating from day 6 of differentiation, whereas stem cell-derived ECs in the 2D cell culture format proliferated as indicated by the combined scRNA data (Figure S2D).

This raised the question of whether the separation of the ECs from the mural cells or the transfer to a 2D culture format reinstates the proliferation of ECs. Fluorescence-activated cell sorting (FACS)-sorted PECAM1^+^ ECs from the 3D suspension culture proliferated and could be passaged over six generations upon plating on a culture dish, but also endothelial progenitors plated with the mural cells could be passaged without reduction of the endothelial progenitor fraction (Figure S1C), demonstrating that growth arrest is associated with the 3D suspension culture format. A further important question was whether sorted ECs and mural cells maintained cell-type stability after transfer into the 2D cell culture format. To evaluate this aspect, we investigated the ratio of PECAM1^+^ and PDGFRB^+^ cells in 2D cell cultures prepared from sorted and unsorted 3D aggregates at day 6 of differentiation. Within sorted PECAM1^+^/PDGFRB^−^ cell cultures, no reappearance of PDGFRB^+^ cells could be detected after 6 days of culturing in a monolayer culture format under EC differentiation medium (Figure S1D). In contrast, PECAM1^−^/PDGFRB^+^-sorted cells lost the PDGFRB expression under the same conditions. Upon plating of the unsorted 3D cell cultures onto a monolayer on day 6, the fraction of PECAM1^+^ cells increased from 64% to 72% after 6 days of culture, whereas the fraction of PDGFRB^+^ cells decreased from 18% to 10% simultaneously. In comparison, in the 3D suspension culture, the fraction of PECAM1^+^ cells decreased, while the fraction of PDGFRB^+^ increased within the same culture interval as seen by the scRNA analysis. This suggests that cell-cell signaling between endothelial progenitor and mural cells within the 3D culture format leads to changed proliferative behavior compared with the 2D cell culture format.

### Single-cell transcriptomics of neovascularization

**Figure 4:**
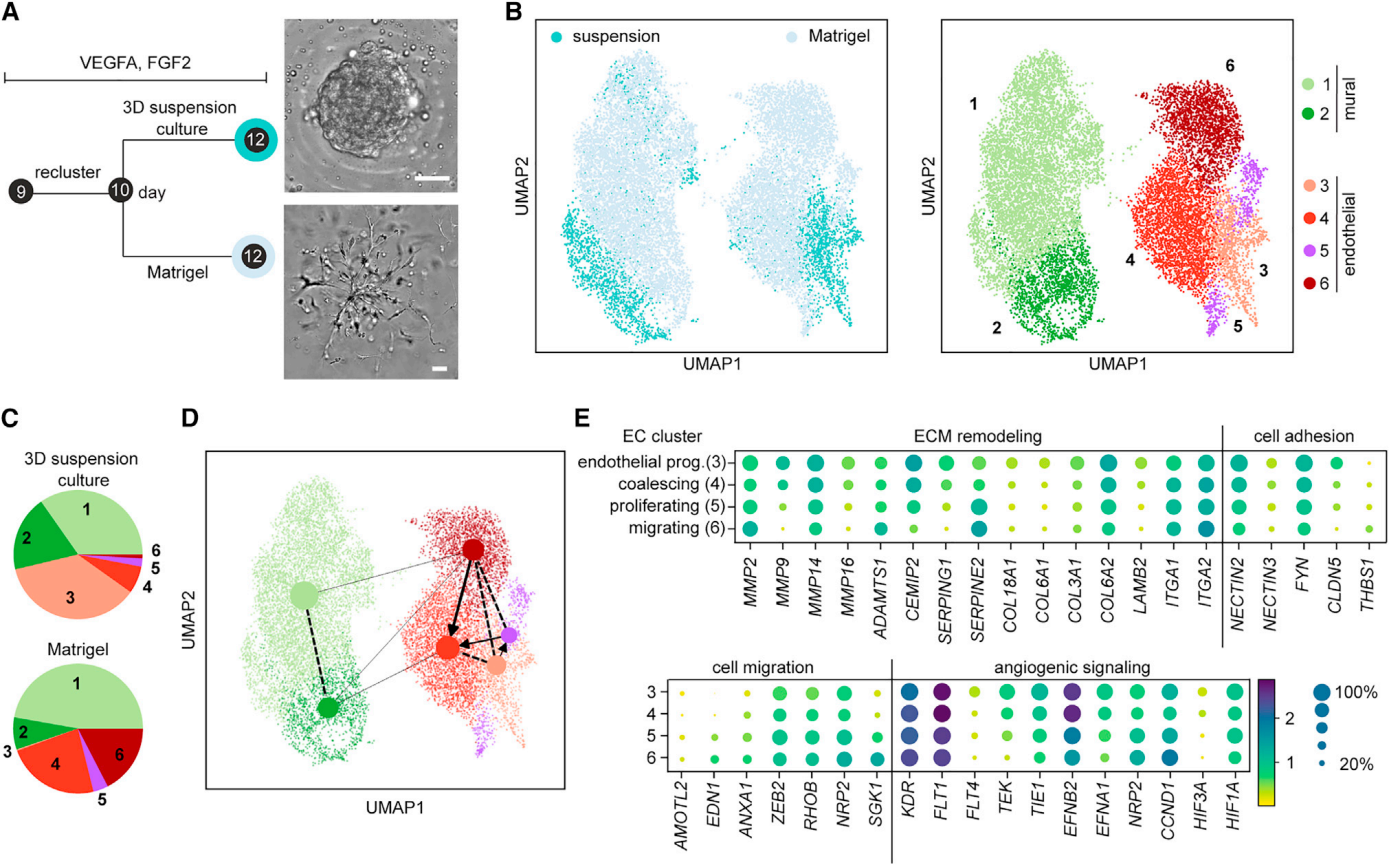
Single-cell analysis of hiPSC-derived ECs undergoing neovascularization in Matrigel (A) Experimental overview of the culturing conditions for microvessel formation induction and representative bright-field images of cell culture morphologies. Scale bar: 50 μm. (B) UMAP plots containing single-cell transcriptomes of cells from 3D suspension and Matrigel cultures. The color code denotes conditions (left) and Leiden cell clusters (right; 1 and 2, mural cells; 3, endothelial progenitor cell; 4, EC coalescing; 5, EC proliferating; 6, EC migrating). (C) Cell type composition of the two culturing conditions is represented as a pie chart. The percentage of each cell population is given in Table S4. (D) Velocity analysis of the single-cell transcriptomic data from (A). (E) Expression levels of representative DEGs sorted by function for the three EC transcriptional states. The color intensity and dot size denote the normalized cluster mean expression level and the fraction of cell expression for the corresponding gene, respectively.

After embedding of the stem cell-derived endothelial progenitor cells from the 3D suspension culture into Matrigel, sprouting was induced within the first 12 h (Figures 4A and S3A). Single-cell transcriptomes of the sprouting Matrigel culture were determined 48 h after transfer and compared with single-cell transcriptomes of cells within 3D suspension cultures kept to the same day of differentiation. Three distinct transcriptomic EC subclusters were detected within the Matrigel culture (clusters 4–6), which separated from the endothelial progenitor cells in the 3D suspension culture (Figure 4B). In addition, mural cells formed two transcriptomic subclusters, where one cluster overlapped with the transcriptomic state of the mural cells in the 3D suspension culture. The expression levels of general cell-type markers for the assignment of mural and EC clusters are shown in Figure S3B. The fraction of mural cells and ECs was comparable to that found in the 3D suspension culture (Figure 4C). A corresponding PAGA analysis on the RNA velocity showed the connectivity between the transcriptional states of ECs and mural cells in the Matrigel and suspension culture cell types (Figure 4D).

For the formation of microvessels, Matrigel-embedded endothelial progenitor cells must migrate into the hydrogel and coalesce. We plotted exemplary DEGs related to ECM remodeling, cell migration and motility, cell interaction, and VEGF signaling (Figure 4E) to determine the genes that induced the processes and assigned transcriptional states to the main EC cluster. Genes associated with cell migration gradually increased from the endothelial progenitor state within the 3D suspension culture to the EC clusters 4 and 6 in Matrigel (e.g., *ANXA1* or *ZEB2*). In contrast, gene expression for cell adhesion (e.g., *NECTIN2/3* or *CLDN5*) and ECM (e.g., *COL3*, *6*, and *18*) gradually decreased from the endothelial progenitor cells to ECs of clusters 4 and 6. This argued that ECs of clusters 4 and 6 represent the coalescing and migrating cell states, respectively. To investigate this further, we stained DEGs between the two clusters within cryosections of hydrogel culture from day 12 of differentiation. For ECs of cluster 4, *DLL4* and *CLDN5* were among the DEGs. Both proteins showed stronger expression in cells with interacting partners than in isolated ECs (Figures S3D–S3G). This further indicated that ECs of cluster 4 are coalescence ECs (cECs). Immunofluorescence (IF) staining of DEGs of cluster 6 was not successful. For example, the strongest DEG of cluster 4 was the serum and glucocorticoid kinase 1 (*SGK1*), for which antibodies in the IF staining were not specific. However, it is known that ablation of *SGK1* in mice leads to a strong reduction of EC migration. Corroboratively, a gene ontology (GO)-term analysis of the DEGs of cluster 6 also indicated the enrichment of genes associated with cell migration (Figure S4A). Furthermore, a TFEA of the DEGs of migrating ECs (mECs) showed enrichment of *ELK3*, *KLF6*, and the epithelial-mesenchymal transition driver *SNAI2* (Figure S4B). Based on these findings, we assigned cluster 6 to mECs. The additional EC cluster 5 can be assigned to proliferating ECs.

Within the DEGs of the ECs were key proteins of the VEGF, Notch, and mTOR signaling pathways. The most obvious was the upregulation of *KDR* and *NRP2* in mECs, both of which act on endothelial motility, sprouting, and survival (Gerhardt et al., 2003). Within the Notch signaling pathway, mECs downregulated *NOTCH1*, *NOTCH4*, *DLL4*, and *JAG1* (Figure S4C). This could be expected, since Notch inhibition in cellular model systems has been shown to induce sprouting, branching, and filopodia induction (Lobov et al., 2007). Most interestingly, the mTOR pathway proteins, particularly those of the mTOR complex 2 (*RICTOR*), were strongly downregulated in mECs. One downstream target of mTORC2 is *SGK1*, which is the top upregulated gene in mECs, indicating strong metabolic regulation in this motile cell state (Zarrinpashneh et al., 2013). Furthermore, *DEPTOR*, an adaptor protein for mTOR complexes 1 and 2, was downregulated and concomitantly a DEG for cECs. IF images showed that in mECs, DEPTOR exhibits a nuclear location, and in cECs, it could be detected in the nucleus and in the cytoplasm, demonstrating regulatory involvement of the mTOR pathway during EC migration (Figure S4C). Notably, the expression levels of integrins changed only slightly between the EC transcriptional states.

### EC maturation in Matrigel

**Figure 5:**
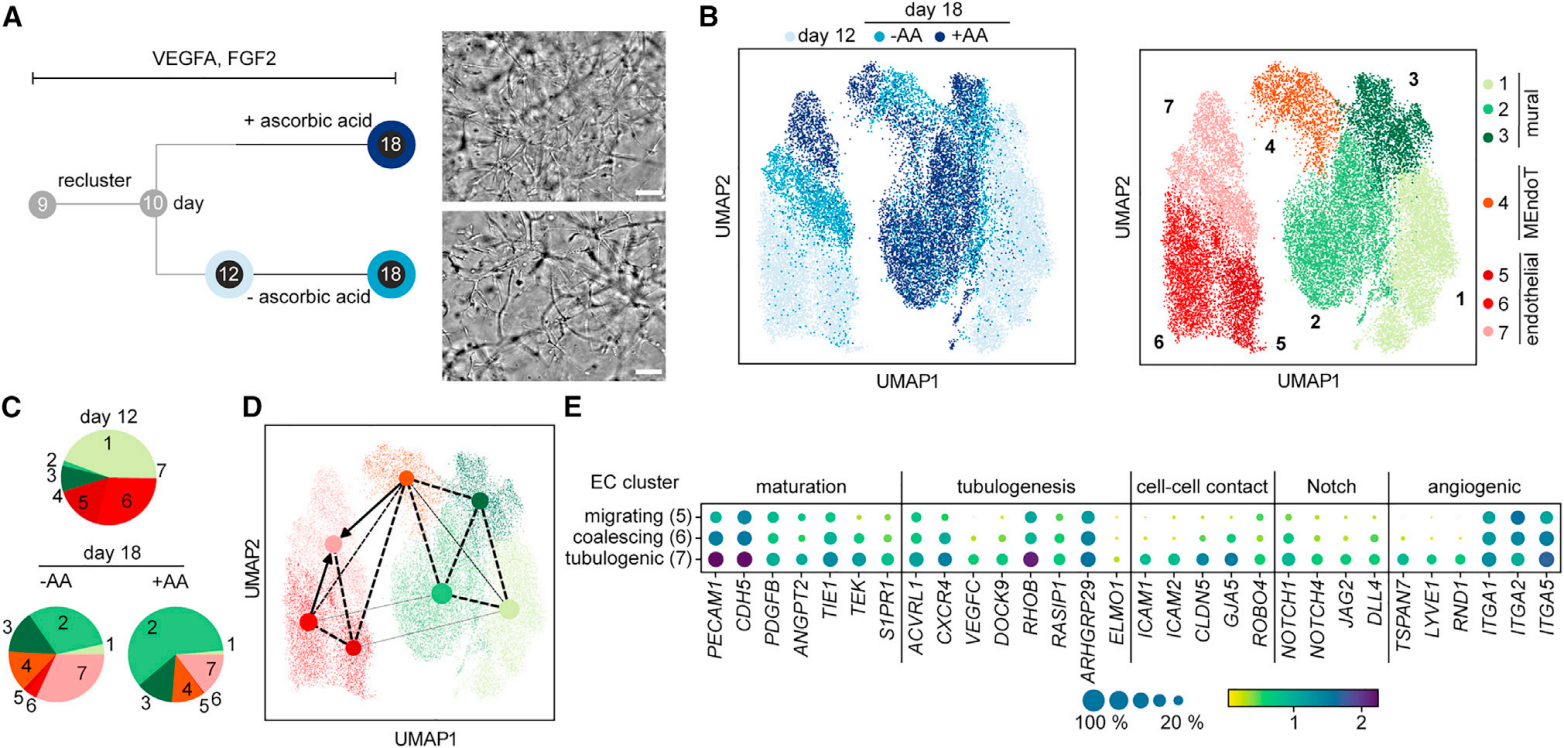
Maturation of endothelial cells in Matrigel in the presence and absence of ascorbic acid (A) Experimental timeline and applied conditions with corresponding bright-field images of stem cell-derived EC and mural cell cultures in Matrigel on day 18. Scale bar: 50 μm. (B) Left: UMAP plot of scRNA-seq data from day 12 and two samples from day 18. Right: UMAP plot colored for the annotated clusters 1, mural cell; 2 and 3, pericytes (P1 and P2); 4, MEndoT; 5, migrating ECs; 6, coalescing ECs; 7, tubulogenic ECs. The dataset contains 13,159, 6,373, and 8,684 cells for days 12 and 18 without and with ascorbic acid in the medium, respectively. (C) Cell-type composition in the samples. Cell-type compositions are also given in Table S4. (D) Overlay of the PAGA and UMAP plots with connectivity lines, indicating the developmental directionality based on the velocity of the single-cell transcriptomes. The dashed lines represent the connectivity between the different clusters. Here, the edge weights indicate the confidence of the connection. The arrows denote a directionality that is based on the mRNA-velocity (spliced vs unspliced). (E) Expression levels of representative DEGs sorted by function for the three EC transcriptional states. The color intensity and dot size denote the normalized cluster mean expression level and the fraction of cell expression for the corresponding gene, respectively.

Upon prolongation of the culturing time, the vessels grew and branched in the Matrigel microenvironment. To determine the genes activated during vessel maturation, we acquired single-cell transcriptomes of day 18 Matrigel cultures. In addition, we investigated the effects of ascorbic acid (AA) on EC maturation. AA increases the synthesis of the basal laminal protein collagen IV and reduces vessel permeability (Utoguchi et al., 1995). Bright-field imaging showed that vessel length and branching network were comparable in the presence and absence of AA (Figure 5A). To extract DEGs responsible for EC maturation, single-cell transcriptomes of all day 12 and 18 Matrigel cultures were clustered together (Figure 5B). ECs formed on day 18 of differentiation in the presence and absence of AA, where a low fraction of migrating and coalescing cells was still observed on day 18 (Figure 5C). Upon addition of AA to the cell culture medium, the fraction of mural cells increased compared with cell culture without AA (Figure 5C). A cell-cycle analysis of the scRNA dataset showed that addition of AA led to an increase in the number of proliferating cells in all cell clusters, but proportionally more in the mural cell cluster (Figures S5A and S5B). The transcriptomes of the mural cells from day 18 converged partially with the transcriptomes of day 12 and formed in total three clusters (clusters 1–3). Next to the EC and mural cell clusters, the scRNA-seq data revealed a cell cluster in which cells expressed mural and endothelial markers, including *PDGFRB* and *PECAM1* (Figure S5C). In addition to the higher expression of endothelial marker genes, a Pearson correlation analysis of the variable genes revealed higher proximity to mural cell clusters than the endothelial ones (Figure S5D). The PAGA connectivity based on the RNA velocity indicated that this population developed from the mural cells toward ECs (Figure 5D), wherefore we assigned these cells to mesenchymal-to-endothelial transitional cells (MEndoT).

Evaluation of the DEGs between ECs revealed that on day 18, ECs increased the expression levels of cell-cell contact genes such as *ICAM*s and *CLDN5* (Figure 5E). Furthermore, the expression of key tubulogenesis genes, such as *RASIP1*, *RHOB*, *ELMO1*, and *ARHGAP29* (Xu and Cleaver, 2011), was upregulated. In addition, the expression level of the *DOCK9* gene, which is a *RAC1* activator responsible for vascular lateral branching (Abraham et al., 2015), increased. Based on the expression pattern, we assigned day 18 ECs to tubulogenic ECs (tECs). To confirm this, we prepared IF staining of ICAM2 and CLDN5 within cryosections of day 12 and 18 hydrogel cultures (Figures S3F and S3G). Both DEG markers increased clearly in vessel structures on day 18. In line with the PAGA analysis, a Pearson correlation of the EC transcriptional states from days 12 and 18 showed that the tubulogenic state was closer to the coalescing than to the migrating state (Figure S5D). In addition to the changes in the levels of genes controlling the structural change, Notch signaling was upregulated again compared with the migrating and coalescent states in the forms of *NOTCH1*, *NOTCH4*, and *DLL4*. By increasing the resolution of the Leiden clustering, it is possible to separate ECs cultured in the presence and absence of AA; however, DEG change was minimal. TF analysis of the DEGs from tECs did not reveal differences in cECs. For comparison, we plotted literature-reported EC cell-type markers for the mEC, cEC, and tEC transcriptional states (Figures S6A and S6B).

To investigate possible toning during the maturation process of tECs, we calculated a gene expression enrichment score of the top 300 DEGs of early arterial and venous cells from human embryos at Carnegie stages 10 and 11 (Zeng et al., 2019) (Figures S6C–S6E) within our day 12 and 18 differentiation dataset. Strikingly, within the top 300 DEGs of the ECs, genes of the early arterial cells increased within tECs but not early venous genes.

### Mural and endothelial cell-cell interaction

**Figure 6:**
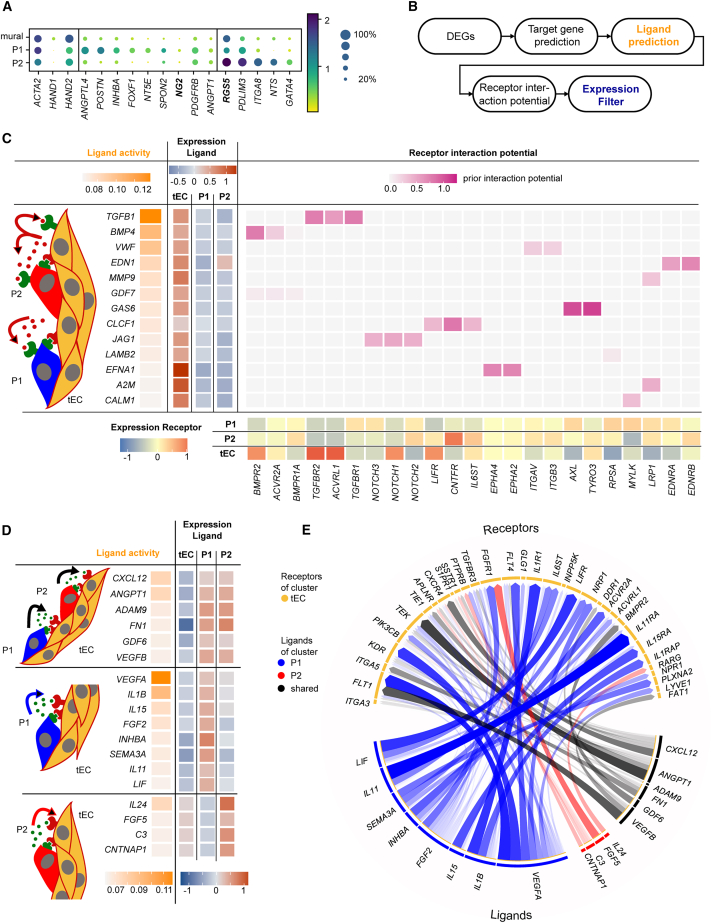
Inferred signaling between endothelial cells and pericytes during vessel maturation from single-cell transcriptomics (A) Gene expression analysis of the mural cells cultured in Matrigel up to day 18 of differentiation. The P1 and P2 clusters show the expression of pericyte markers (bold). The color intensity and dot size denote the normalized cluster mean expression level and the fraction of cell expression for the corresponding gene, respectively. (B) NicheNet analysis workflow with the expression filter to infer ligand-receptor interactions. (C) Ligand-receptor pairs inferred with NicheNet, where single-cell transcriptomes of tECs are used as senders and the combined P1 and P2 pericyte subpopulations as receivers for the analysis. (D and E) Ligand-receptor pairs inferred with NicheNet, where single-cell transcriptomes of the P1 and P2 pericyte subpopulations are used as senders and tECs as receiver cells for the analysis. Overlapping (top rows) and unique pericyte ligands (middle and bottom rows) are listed in table format. Ligand-receptor pairs are presented in the circular chord diagram.

The single-cell transcriptomes of the microvascular culture on day 18 revealed that mural cells of clusters 2 and 3 (Figure 5B) expressed pericyte markers *NG2*, *RGS5*, or *NT5E*. Among the DEGs were further genes previously found to be enriched in pericytes, such as *POSTN* and *PDLIM3*. IF stainings of cryosections from day 18 hydrogel cultures showed that, indeed, PDGFRB-positive cells aligned on the PECAM1 cells (Figure S7). Therefore, we assigned mural clusters 2 and 3 as pericytes, P1 and P2, respectively. While cells of the P1 cluster expressed the TF *FOXF1*, cells of the P2 expressed *GATA4* (Figure 6A), whereas both were found in the primary pericytes (Muhl et al., 2020). Localization of the subcellular pericytes within the Matrigel culture failed due to either the low amount of specific antibodies for the DEGs or the cross-expression of the markers. Mural cells of cluster 1 consisted mainly of cells from day 12 and clustered with mural cells of the 3D suspension culture. The higher expression level for the mesenchymal TF *HAND1/2* and *ACTA2* argued that mural cells of cluster 1 were in a premature stage. The velocity analysis of the mural cell transcriptomes, however, did not show any direction or evolving latent time, highlighting the plasticity of the cells. To support the mural cell assignment, we plotted literature-reported mesenchymal and fibroblast cell-type markers for the mural transcriptional states (Figure S6F).

In the last step, we asked whether it is possible to infer cell-cell communication signals leading to vessel maturation from the scRNA datasets of highly plastic cell types, stability, reduced proliferation, and migration. To trace cell-cell signaling between pericytes and tECs, we performed a ligand-receptor analysis on the single-cell transcriptomic data. Algorithms evaluating only ligand-receptor expression profiles could not recapitulate literature-confirmed signals between ECs and pericytes, for example, *TGFB1* or *VWF*, in our dataset. Therefore, we performed a NicheNet analysis (Browaeys et al., 2020), which infers ligand-target links between interacting cells by combining DEG data with prior knowledge of signaling and gene-regulatory networks (Figure 6B). NicheNet was applied bidirectionally by investigating ECs as ligand sender and receiver cells. The NicheNet results, that is, ligand activities and receptor and target gene interaction potential, were further filtered for gene expression levels to increase the probability of finding relevant interaction links (Figure 6C). For ECs as senders and pericytes as receivers, the algorithm predicted strong ligand activity for *TGFB1*, *BMP4*, and *VWF*. All three have been reported previously to be central for communication between both cell types (Sweeney and Foldes, 2018). *TGFB1* acts in paracrine and autocrine signaling, while tECs express the *TGFB1* receptor *ALK1* (*ACVRL1*) and pericytes express *ALK5* (*TGFBR1*), which is consistent with current reports using *in vitro* culture systems (Dave et al., 2018). The inferred ligand activity for *PDGFB* was low and thus filtered out, although *PDGFB* was highly expressed. This is explained by the fact that we used DEGs of the entire differentiation trajectory to rank ligands, and since *PDGFB* is always expressed strongly, the corresponding target genes within the DEG cannot be expected. In turn, the top predicted ligand activities represented signals that occurred during vessel maturation rather than during vessel formation and the pericyte recruitment phase. Individual NicheNet analyses between ECs and P1 or P2 cells did not differ from the combined analysis. In contrast, the NicheNet analysis for P1 or P2 as signal senders and tECs as the receivers showed that P1 and P2 pericytes exhibited shared, as well as individual, ligands (Figure 6D). The shared ligands included *CXCL12*, *ANGPT1*, fibronectin, and *GDF6*, which antagonize VEGF signaling to promote junctional stability and vascular integrity (Krispin et al., 2018; Salcedo and Oppenheim, 2010). The strongest individual predicted ligand activity of P1 cells was *VEGFA*, followed by a set of cytokines (*IL1b* and *IL15*) and *FGF2*. For P2 cells, the ligands with the strongest individual activity were *IL24* and *FGF5*. The corresponding tEC receptors are shown in Figure 6E. All inferred cytokines have proven pro- or anti-angiogenic function in cancer angiogenesis (Table S4); however, their function during neovascularization and vessel maturation is unknown.

**Figure 7:**
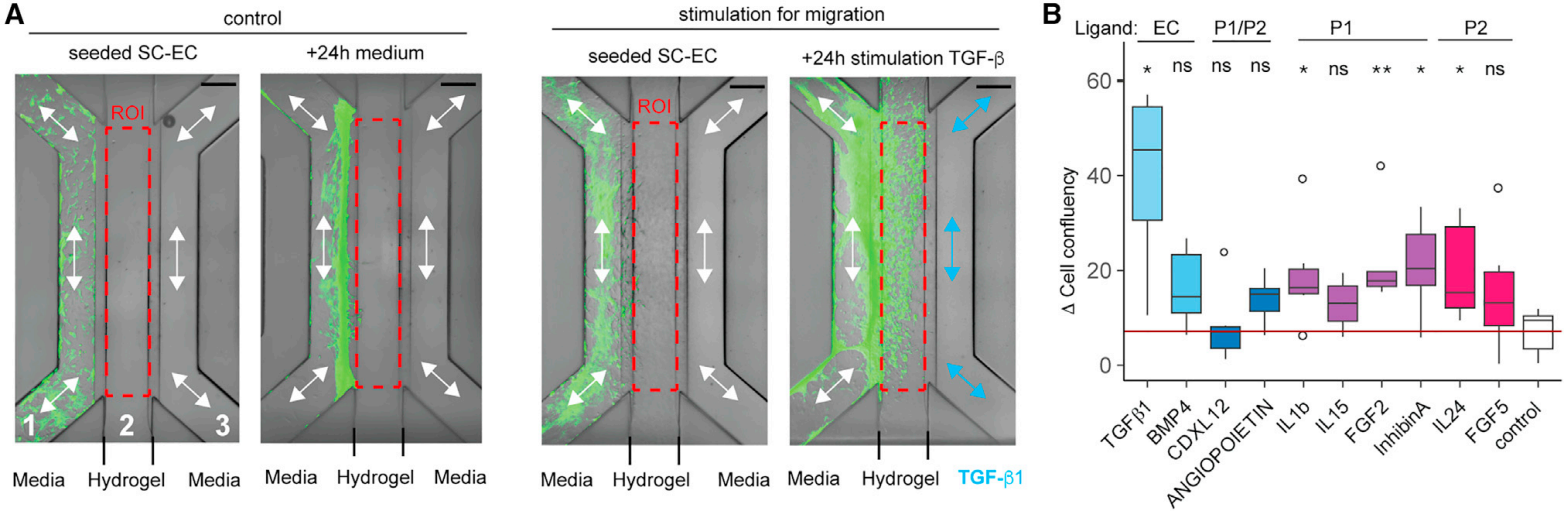
Testing of inferred pericyte ligands to attract stem cell-derived endothelial cells (SC-ECs) (A) Microfluidic chip for testing the ligand activity. While GFP-expressing SC-ECs were seeded within the left microchannel of the chip, within the right microchannel the ligand was applied. Migration of SC-ECs into the Col-1 hydrogel was evaluated by measuring the cell confluency in the region of interest after 24 h of stimulation (ROI, red dashed box). Scale bar denotes 100 μm. (B) Boxplot shows the increase in cell confluency in the hydrogel area (ROI) in the presence of the ligand within the opposing microchannel. A Mann-Whitney U test with exact p value estimation was performed for significance testing (n ≥ 4 for each ligand). Significance levels are ^∗^p < 0.05, ^∗∗^p < 0.01.

To demonstrate that the identified signaling proteins have an angiogenic function, we performed an *in vitro* cell migration assay to test the inferred ligands for their capability to attract stem cell-derived ECs (SC-ECs). For this, we selected 10 ligands from the cell-cell communication analysis (Figure 6D), i.e., two EC, two P1/P2, four P1, and two P2 sender ligands. The migration assay was established on a commercially available microfluidic chip platform, which comprised three converging microfluidic channels (Figure 7). Within the middle microchannel, a COL1 hydrogel was formed to fluidically separate the two outer microchannels. We tested if SC-ECs enter the COL1 hydrogel from one microchannel side when adding the ligand on the opposing microchannel. The attractant capability of the ligands was evaluated by the increase in cell confluency in the hydrogel area compared with a negative control experiment with cell medium only. Five of the 10 tested ligands significantly increased the migration of the SC-ECs. TGF-β1 was one of the ligands that showed a high activity for attracting SC-ECs, but was expressed only by ECs and thus acted in the 3D cell culture system in autocrine mode. While the ligands IL-1b, InhibinA, and FGF2 are paracrine-active ligands expressed from pericytes of the cell cluster P1, IL-24 was a paracrine-active ligand expressed from pericytes of cell cluster P2. In summary, the analysis revealed signaling molecules acting between pericytes and ECs during vasculogenesis. Further, the resolved expression heterogeneity of ligands between pericytes argues for functional differences between P1 and P2 during vessel maturation.

## Discussion

Here, we investigated the differentiation of hiPSCs into ECs in a 3D suspension culture and, subsequently, the process of neovascularization with the stem cell-derived ECs and evolving mural cells at the single-cell level. We found that the differentiation trajectory in the early stage of ECs and mural cells in a 3D suspension culture resembled the development of that in a monolayer format. In both cell culture approaches, one common mesodermal progenitor cell type is formed at day 3 of differentiation before the EC and mural cells evolve. In contrast to the 2D cell culture approach, ECs become quiescent in the 3D suspension culture without entering the neovascularization process. Cell communication between endothelial progenitor cells and mural cells exists within the 3D suspension culture, which is exemplified by the finding that, upon separation from the ECs, the plastic mural cells lost mesenchymal cell-type marker expression of *PDGFRB*. Mesodermal cells express *TGFB1* and *PDGFB*, where the combination of these two factors is used to induce vascular smooth muscle cell development *in vitro* in the same mesodermal precursor cells (Patsch et al., 2015). Lineage specification from day 6 of differentiation into an arterial tone could be observed when taking the stronger expression level of *EFNB2* into account (Zhang et al., 2017) (Figure 1C). Within the hydrogel culture, transcriptomes of the SC-ECs showed high gene enrichment scores for early arterials in embryos from Carnegie stages 10 and 11, confirming the toning (Figures S6E and S6F). Tissue-specific EC gene enrichment could not be detected. For scaling EC production with a 3D suspension culture, the transcriptional cell-type analysis argues that 6 days of differentiation is optimal due to the largest EC-to-mural cell ratio.

Transfer of the 3D suspension culture into Matrigel led to sprouting of the ECs. Single-cell transcriptomics untangles migrating, coalescent, and tubulogenic EC states during neovascularization. In comparison with the *in vivo* reported data, after laser ablation in the choroid layer of mouse eyes, the cellular complexity is far lower. In addition to the expected VEGF and Notch signaling pathways, the mTOR pathway is regulated during neovascularization. mTOR kinase controls various processes; when complexed in the mTORC1, it controls predominantly the cell metabolism. In the metabolism, for example, a switch between OXPHOS and glycolysis for mECs could not be observed as described for tip cells *in vivo* (Rohlenova et al., 2020). However, in mECs, mTORC2 adaptor proteins were downregulated, and the downstream effector kinase *SGK1*, which controls cell survival during angiogenesis (Catela et al., 2010) and EC shape (Tsuji-Tamura and Ogawa, 2016), was strongly upregulated; its ablation led to reduced neovascularization and impaired cell migration (Zarrinpashneh et al., 2013). The function and regulation of *SGK1* through mTORC2 are unknown, but have become the focus of further investigations.

Vessel structure formation within the hydrogel culture was accompanied by a change in ECM-integrin expression level. While *ITGA2* was upregulated in mECs, a gradual increase in *ITGA1* and *ITGA5* was detected toward the cEC and tEC stages (Figure 5E). Blocking collagen-binding ITGA1 and ITGA2 with antibodies has been shown to reduce angiogenesis (Senger et al., 2002). The EC and mural cell stage-assigned integrin profiles provide new dynamic insights. The addition of AA to the cell culture medium increased the expression level and deposition of the central basal lamina protein COL4 (Figure S6E). Therefore, the AA-induced maturation of ECs increased the proliferation rate of mural cells. This may be unwanted in long-term cultures during tissue engineering with no proliferative cells. ECs on day 18 of differentiation did not show a specification marker for arterial or any other vascular cell type. Notably, the scRNA-seq data from day 18 of the Matrigel cell cultures indicated a mesenchymal-to-endothelial transition. Such a mural cell plasticity has been observed before in heart tissue after injury (Ubil et al., 2014), but not in *in vitro* conditions.

The most compelling results of the single-cell transcriptomic analysis during the vessel maturation phase in a reduced *in vitro* microenvironment were the resolved cell-cell communication signals between the two transcriptional pericyte subpopulations and tECs. Pericytes are recruited by PDGFB signaling to capillary walls to stabilize integrity and tube assembly (Lindahl et al., 1997). Next to the known EC-pericyte signaling factors, including further FGF2 and VEGFA, the complex cytokine profiles of the pericytes was resolved by the directionality of the NicheNet ligand-receptor analysis. Here, ligands from the two transcriptional pericyte states, P1 and P2, were inferred, and their activity in attracting EC migration, i.e., IL-1b or IL-24, respectively, was confirmed. In addition, we showed experimentally that inferred *TGFB1* autocrine signals, where sender and ligands are expressed only by tECs, are active in attracting SC-ECs in an *in vitro* assay. The previous signaling axis CXCR4/CXCL12 for pericyte vessel maturation (Salcedo et al., 1999) was also here detected as CSCL12 sent from pericytes to ECs. However, ligand testing of CXCL12 for attraction of EC migration was negative. The present single-cell transcriptomic data and analysis of the vascular structure formation process can be used as a benchmark set for future *in vitro* vascularization approaches, differentiation attempts to alter the specification of ECs, or investigation into disease-specific gene functions.

## Experimental procedures

### Resource availability

#### Corresponding author

Further information should be directed to Matthias Meier (matthias.meier@helmholtz-munich.de).

#### Materials availability

This study did not generate new unique reagents nor use new biological samples.

#### Data and code availability

The code for scRNA-seq analysis has been deposited on https://github.com/MeierLabMiBioEng/scRNA_3D_EC_differentiation GitHub and is publicly available. DOIs are listed in the key resources table. Raw data are publicly available on the Gene Expression Omnibus repository under accession code GSE196799. Any additional information required to reanalyze the data reported in this paper is available from the corresponding author upon request.

### Experimental model details

When not specified otherwise, experiments were conducted using a hiPSC line, which was kindly provided by Prof. Lickert (Helmoltz Zentrum Munich) and is registered in the human pluripotent stem cell registry (https://hpscreg.eu) under the name HMGUi002-A. The cell line was derived from a Caucasian male donor. The control iPSC line was purchased from the Coriell Institute (Coriell Institute, cat. no. AICS-0036-028). The cell line (AICS) expressed mEGFP constantly from the AAV1 locus and was derived from a parental line from an Asian donor. The general scientific use of the HMGUi002-A cell line was approved by the local ethics committee at the Technical University Munich (reference no. 400/21 S-KH).

### Two-dimensional hiPSC cell culture

Human iPSCs were cultured on hESC Matrigel-precoated six-well plates according to the manufacturer’s recommendations (Corning, cat. no. 354277) in mTeSR1 medium (StemCell Technologies, cat. no. 85850) at 5% CO_2_, 5% O_2_, and 37°C with daily medium change and passaged twice a week in a 1:6 ratio using 0.05% trypsin-EDTA (Sigma Aldrich, cat. no. T4174). The hiPSC culture was free of mycoplasma contamination as tested by the MycoSensor PCR assay kit (Agilent Technology, cat. no. 302109).

### Three-dimensional suspension culture

For the transfer into a 3D hiPSC cell culture, the medium was aspirated, cells were washed with 2 mL PBS−/−, and 500 μL Accutase (Sigma Aldrich, cat. no. A6964) was added. During incubation at 37°C for 5 min, the cells detached. Addition of 2.5 mL mTeSR stopped the Accutase reaction. The wells were washed with 1 mL mTeSR before centrifugation for 5 min at 200 × *g* and resuspended in 500 μL mTeSR with 10 μM Rock inhibitor Y-27632 dihydrochloride (Abcam, cat. no. HY-10583) and 1% penicillin/streptomycin (P/S) (Thermo Fisher Scientific, cat. no. 15140122). Cells were transferred to six-well ultra-low-attachment plates (Corning, cat. no. 3471) at a cell concentration of 5 × 10^5^ cells/mL. The plate was placed on an orbital shaker in the cell incubator and rotated at a frequency of 100 rpm.

### Three-dimensional suspension culture differentiation to ECs

On day −1 of the differentiation, cells were transferred into low-attachment six-well plates at a concentration of 1.5 × 10^6^ cells/well. The differentiation protocol was adopted by combining the protocols from Olmer et al. (2018), Patsch et al. (2015), and Wimmer et al. (2019). From day 0 to day 3 of differentiation, Neurobasal medium with B27 supplement (Life Technologies, cat. nos. 21103049 and 12587010) supplemented with BMP4 (25 ng/mL) (PeproTech, cat. no. 120-05ET-10) and CHIR 99021 (7.5 μM) (Axon Medchem, cat. no. 1385) was used without medium exchange. Medium was changed daily from day 3 to day 7 of differentiation using StemPro-34 (Life Technologies, cat. no. 10639011) with VEGFA (200 ng/mL) (PeproTech, cat. no. 10-20-100) and forskolin (2 μM) (Abcam, cat. no. ab120058). Afterward, StemPro-34 with VEGFA (30 ng/mL) and FGF2 (30 ng/mL) (Miltenyi Biotech, cat. no. 130-093-838) was used with exchange after 2 days.

### Two-dimensional endothelial and mural cell culture

Sorted endothelial and mural cells were cultured in T75 flasks (Sarstedt, cat. no. 83.3911.002). Flasks were coated with a 5 μg/mL fibronectin (Life Technologies, cat. no. 33010018) solution before use. Endothelial and mural cells were cultured in StemPro with 15% fetal bovine serum (FBS), 100 ng/mL VEGFA, and 100 ng/mL FGF2.

### Hydrogel cell culture

For cultivation in 24-well plates (Corning, cat. no. 353047), 100 μL undiluted hESC-Qualified Matrigel (Corning, cat. no. 354277) was added into each well of the plate and incubated at 37°C for 1 h. Aggregates were centrifuged at 800 rpm for 5 min at 4°C, resuspended in 80 μL of Matrigel, and spread on top of the first hydrogel layer. Matrigel polymerization was done at 37°C inside the cell incubator on ice to allow a slow temperature adjustment. After 1 h, 0.5 mL medium (StemPro-34 with 100 ng/mL VEGFA, 100 ng/mL FGF2, and 15% FBS; Fisher Scientific, cat. no. 15377636) was added for cultivation. The medium was exchanged every second day. Samples with AA contained 60 μg/mL AA (Sigma Aldrich, cat. no. A4544). For imaging, cell aggregates were embedded in Matrigel on μ-Slides (IBIDI, cat. no. 80826) with 10–20 per well.

### Cell analysis

Standard FACS, flow cytometry, and cryoembedding procedures are given in the supplemental experimental procedures.

### Cell-type stability experiments

On day 6, aggregates were harvested and prepared for FACS (see Figure S5), and ECs (PECAM1^+^, PDGFRB^−^) and mural cells (PDGFRB^+^, PECAM1^−^) were sorted. The sorted cell types (2 × 10^5^ each) and the unsorted mixture were seeded and cultured in six-well plates coated with fibronectin bovine (Life Technologies, cat. no. 33010018). The medium composition was equivalent to the 3D differentiation culture from day 6 on.

### Fluorescence imaging

Slides were washed with PBS, permeabilized in PBS with 0.1% Triton (Sigma Aldrich, cat. no. 93443) for 30 min at room temperature, washed with 0.2% Tween 20 in PBS (PBST), and blocked with 2% BSA (Proliant Biologicals, cat. no. 68700) in PBST for 1 h. Antibodies were applied in the concentrations recommended by the manufacturer’s specifications in the blocking solution. After primary and secondary antibody staining, five washing steps of 5 min with PBST were performed. Before confocal imaging (Zeiss Axio Observer LSM 880), Vectashield mounting medium (Biozol Diagnostica, cat. no. VEC-H-1000) was added to the sample, and it was covered and sealed by a coverslip.

### Image analysis

IF and bright-field images were corrected for brightness and contrast with ImageJ. Z projection of fluorescence images was performed using maximal intensity. ImageJ version 1.52p was used (Schindelin et al., 2012).

### Cell migration assay

A detailed description of the microfluidic cell migration assay is given in the supplemental experimental procedures.

## Supplemental information

Document S1. Figures S1–S7, Tables S3, and supplemental experimental procedures

Table S4. The table contains the differentially expressed genes for the cell clusters found in single cell transcriptome analysis of the main figures

Document S2. Article plus supplemental information
