# Supplementary material for: Single-cell characterization of neovascularization using hiPSC-derived endothelial cells in a 3D microenvironment
Source: Stem Cell Reports. 2023 Sep 14;18(10):1972–86. doi: 10.1016/j.stemcr.2023.08.008 (PMC10656300; doi:10.1016/j.stemcr.2023.08.008)
Supplement: Document S1. Figures S1–S7, Tables S3, and supplemental experimental procedures [file mmc1.pdf]

Stem Cell Reports, Volume *18*

Supplemental Information

Single-cell characterization of neovascularization using hiPSC-derived endothelial cells in a 3D microenvironment

Simon Rosowski, Caroline Remmert, Maren Marder, Misao Akishiba, Judith Bushe, Annette Feuchtinger, Alina Platen, Siegfried Ussar, Fabian Theis, Sandra Wiedenmann, and Matthias Meier

Supplemental Figures and legends

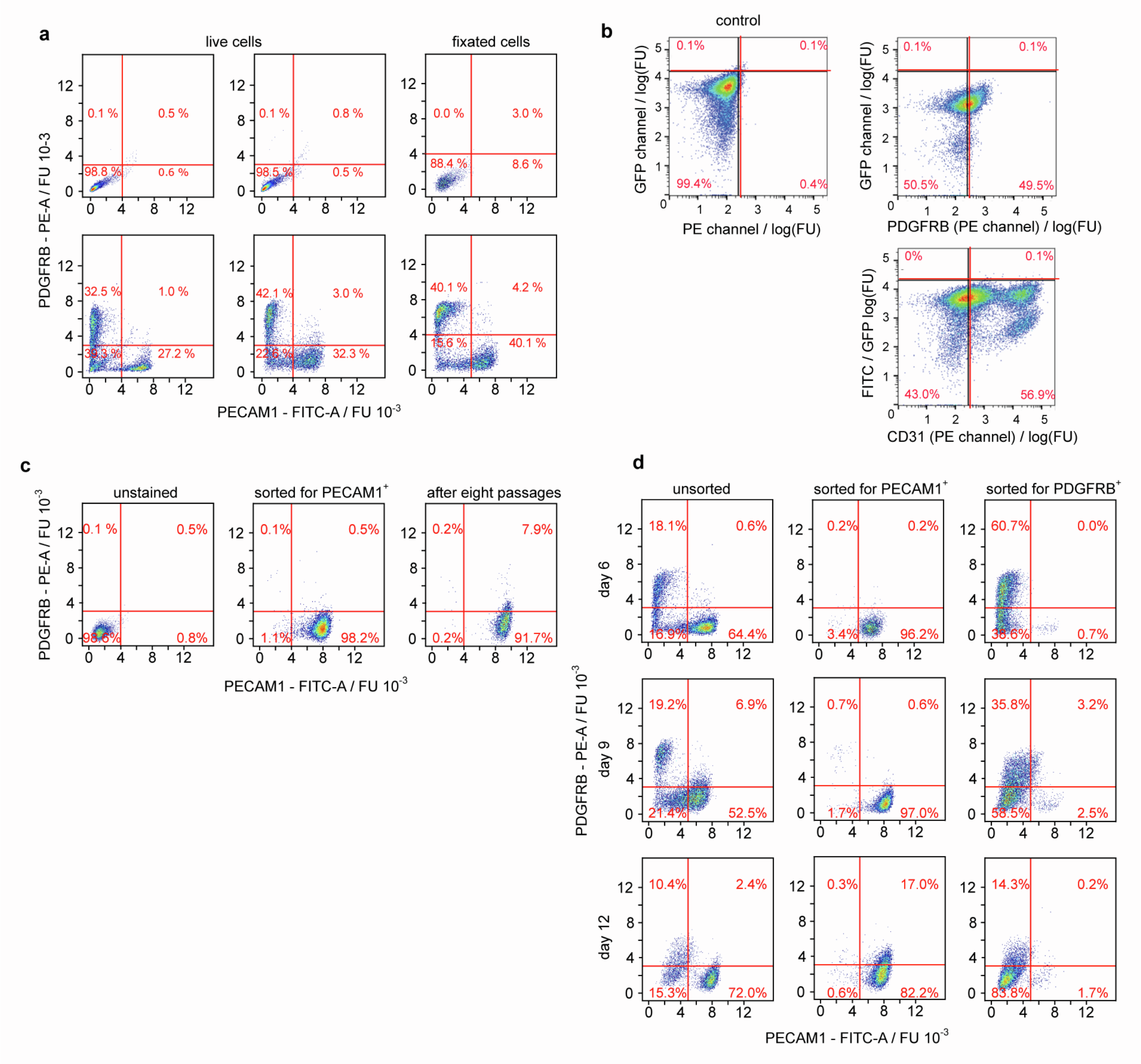

Figure S1. Reproducibility and Cell type stability and long-term culturing of stem cell-derived endothelial cells. **a**, Flow cytometric analysis of three independent EC differentiation experiments. Cells (HMGUi002-A) were stained with PECAM1-FITC and PDGFRB-PE antibodies on day nine of differentiation. Upper left, unstained cell sample control; upper center and right, isotype control of the first EC differentiation experiment; lower row, three independent EC differentiation experiments. **b**, Repeat the EC differentiation with a second fluorescently labeled hiPSC line (*Coriell Institute, Cat# AICS-0036-028*). Fluorescently labeled cells (AICS) were stained with PECAM1-PE or PDGFRB-PE antibodies on day ten of differentiation. Left: FACS plot shows the unstained cell sample control with the intrinsic fluorescence of the GFP reporter signal. Right: FACS plots of the stained sample. **c**, Flow cytometry analysis of sorted PECAM1 positive cells from 3D suspension cultures at day nine of differentiation. Cells are plated on fibronectin coated well plates for long-term culturing in 2D cell culture format within EC maturation media. Flow cytometry analysis of the cells culture in 2D after eight passages showed a comparable fraction of PECAM1 positive cells. **d,** Cell type stability of ECs and mural cells in a 2D culture format under EC maturation media condition. Upon plating 3D suspension cultures on day six of differentiation onto a 2D fibronectin coated 2D culture surface the fraction of PDGFRB positive cells gradually decreased over the time of six days (left panel from top to

bottom). PECAM sorted EC from day six 3D cell cultures maintained their PECAM marker expression over six days within the 2D culture format (middle panel from top to bottom). This is following **c** that PDGFRB sorted mural cells from day six 3D cell cultures lost the expression of PDGFRB over the monitored time frame, which argued that the cell lost their identity. The figure corresponds to the main **Figure 1**.

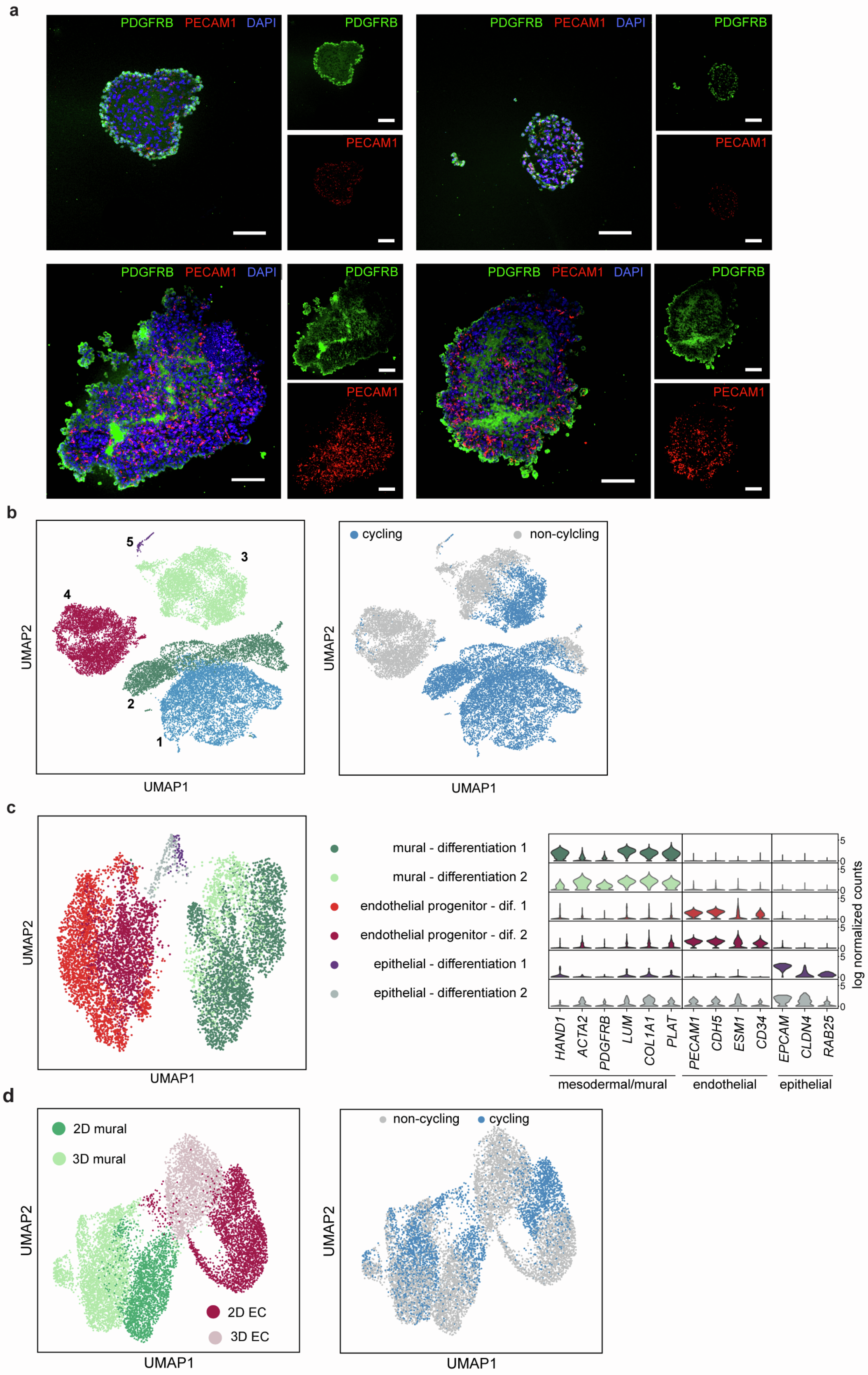
a
PDGFRB PECAM1 DAPI
PDGFRB
PECAM1
b
5
3
4
2
1
UMAP2
UMAP1
cycling
non-cylcling
c
mural - differentiation 1
mural - differentiation 2
endothelial progenitor - dif. 1
endothelial progenitor - dif. 2
epithelial - differentiation 1
epithelial - differentiation 2
HAND1
ACTA2
PDGFRB
LUM
COL1A1
PLAT
PECAM1
CDH5
ESM1
CD34
EPCAM
CLDN4
RAB25
mesodermal/mural
endothelial
epithelial
log normalized counts
d
2D mural
3D mural
2D EC
3D EC
non-cycling
cycling

Figure S2. Cell cycle analysis and reproducibility of the endothelial differentiation in a 3D suspension culture. a, **Left**: UMAP plot of the scRNA-seq dataset from experiment 1 (experiment from the main **Figure 1**), where cells in the G2, S, and M phases were colored in blue, and cells in the G1 phase in grey. **Right:**, UMAP plot corresponding to the left plot with color code for Leiden clustering and cell type annotation. **b**, **Left**: UMAP plot of single-cell transcriptomes from two independent differentiation experiments. The color code denotes the cell types found in the two differentiations. 6618 cells were analyzed in the first and 5035 in the second sequencing experiment. **Right:** Violin plot of common cell type marker genes for annotation of the clusters. The density distribution indicates the normalized cluster mean expression. **c**, **Left**: UMAP plot shows the combined sc-transcriptomes acquired for EC differentiated in 3D suspension and 2D dish culture up to day nine (our study) and eight (McCracken et al.), respectively. **Right**: UMAP plot shows the cells with gene expression relating to S, G2, or M-phase (blue). Cells expressing genes indicative of the G1 phase are denoted with a grey color. The figure corresponds to the main **Figure 3**.

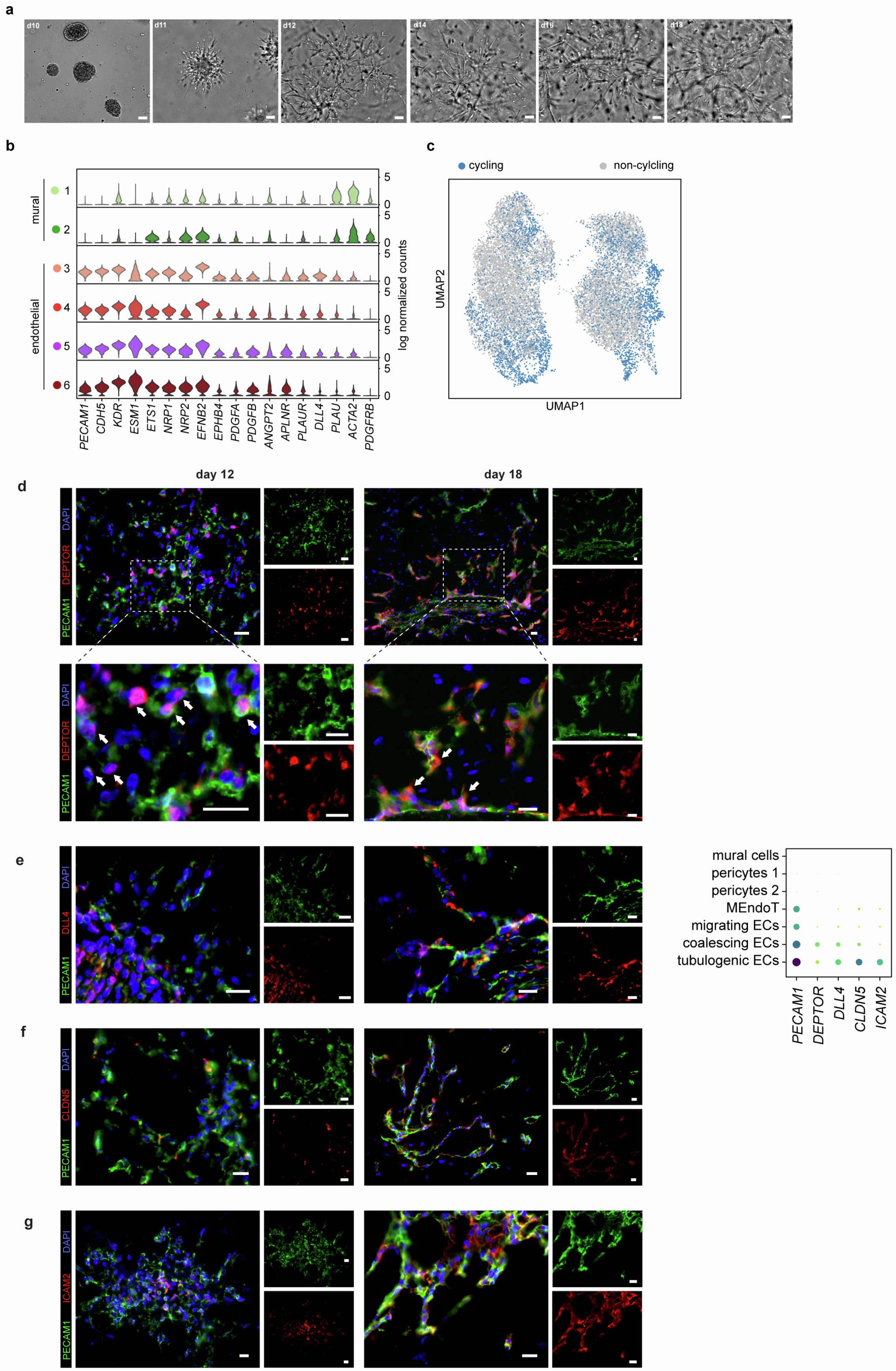

a
d10
d11
d12
d14
d16
d18
b
mural
endothelial
1
2
3
4
5
6
PECAM1
CDH5
KDR
ESM1
ETS1
NRP1
NRP2
EFNB2
EPHB4
PDGFA
PDGFB
ANGPT2
APLNR
PLAUR
DLL4
PLAU
ACTA2
PDGFRB
log normalized counts
c
cycling
non-cylcling
UMAP2
UMAP1
d
day 12
day 18
DAPI
DEPTOR
PECAM1
e
DLL4
f
CLDN5
g
ICAM2
mural cells
pericytes 1
pericytes 2
MEndoT
migrating ECs
coalescing ECs
tubulogenic ECs

Figure S3. Single-cell transcriptomes and immunofluorescence (IF) analysis of ECs and mural cells cultured in Matrigel. **a**, Bright-field images of the Matrigel culture along the timeline of days 10 to 18. Scale: 50 µm. **b**, Violin plot of representative marker genes used to assign the cell clusters in the UMAP plot in main Fig 4. The density distribution indicates the normalized cluster mean expression. **c**, UMAP plot shows the cells with gene expression relating to S, G2, or M-phase (blue). Cells expressing genes indicative of the G1 phase are denoted with a grey color. IF stainings show the protein signal of the differentially expressed genes indicative of the migrating (**d**, **e**), coalescing, and tubulogenic ECs (**f-g**). Arrows in **d**, day 12 (lower left panel) highlight the location of DEPTOR in the nucleus, while on day 18 (lower right panel) DEPTOR is expressed in the nucleus and cytoplasm. Scale: 20 µm. **h**, Dot plot of mRNA expression of genes whose corresponding protein staining is shown above. The color intensity and dot size denote the normalized cluster mean expression and the fraction of cells expressing the gene, respectively. *n*=4 images have been acquired for each condition. The figure corresponds to the main **Figure 4 and 5**.

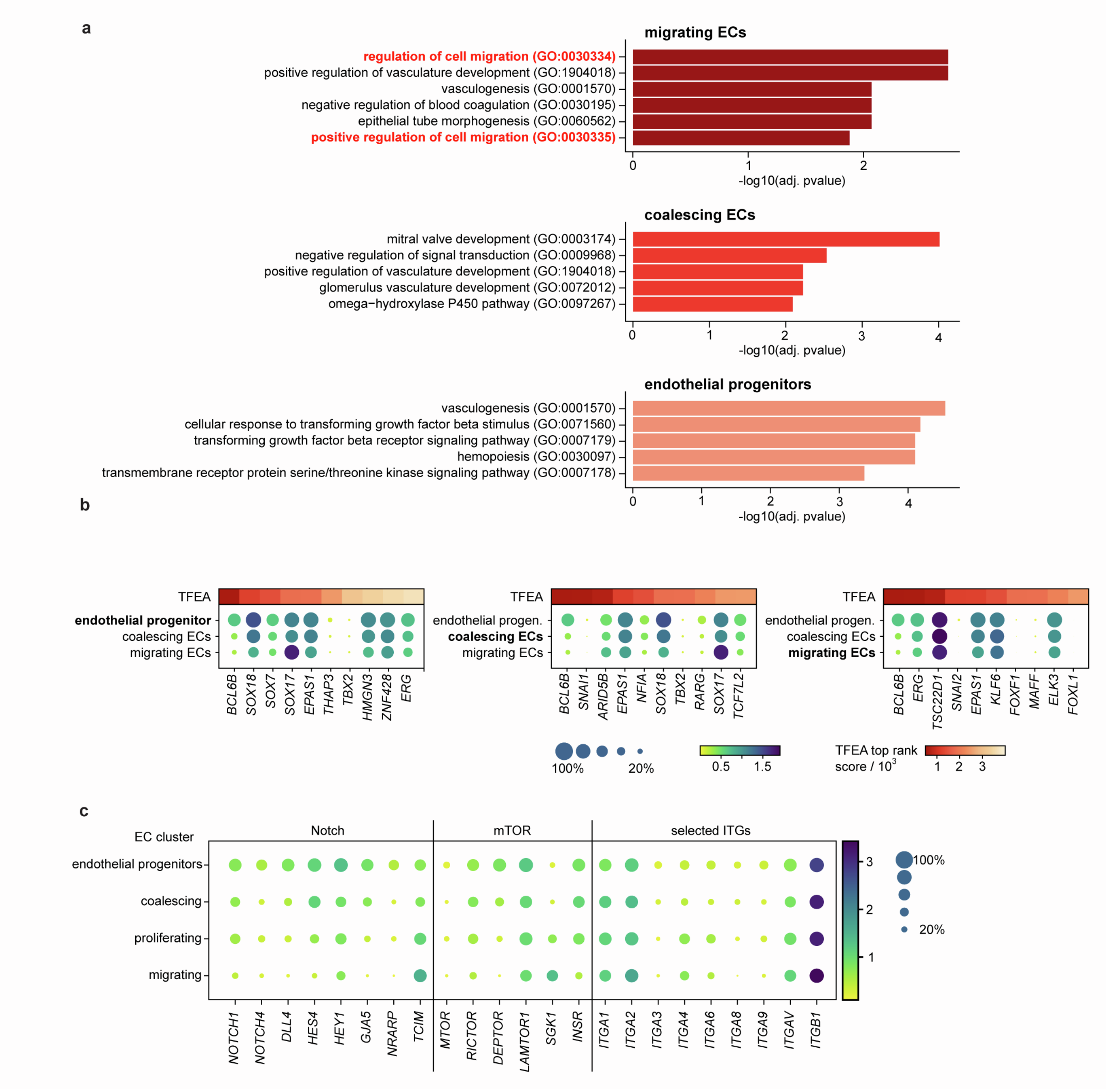

Figure S4. Signal pathway analysis during neovascularization at day 12 of differentiation in Matrigel. **a**, Gene ontology term analysis based on DEGs from the sc-transcriptomes of endothelial progenitor cells, coalescing and migrating ECs. **b**, Transcription factor enrichment analysis based DEGs the endothelial progenitor cells (left), coalescing (middle), and migrating (right) EC cluster. DEGs between the EC clusters were filtered for expression and standard deviation before TFEA. TFEA scores are represented in a color code, whereas the mean expression levels of the corresponding TFs as dot plots. **c**, Dot plot of expression of genes within the mTOR and Notch pathway regulated during the cell state transition from endothelial progenitor cells to coalescing ECs and sprouting ECs. The color intensity and dot size denote the normalized cluster mean expression and the fraction of cells expressing the gene, respectively. The figure corresponds to the main **Figure 4**.

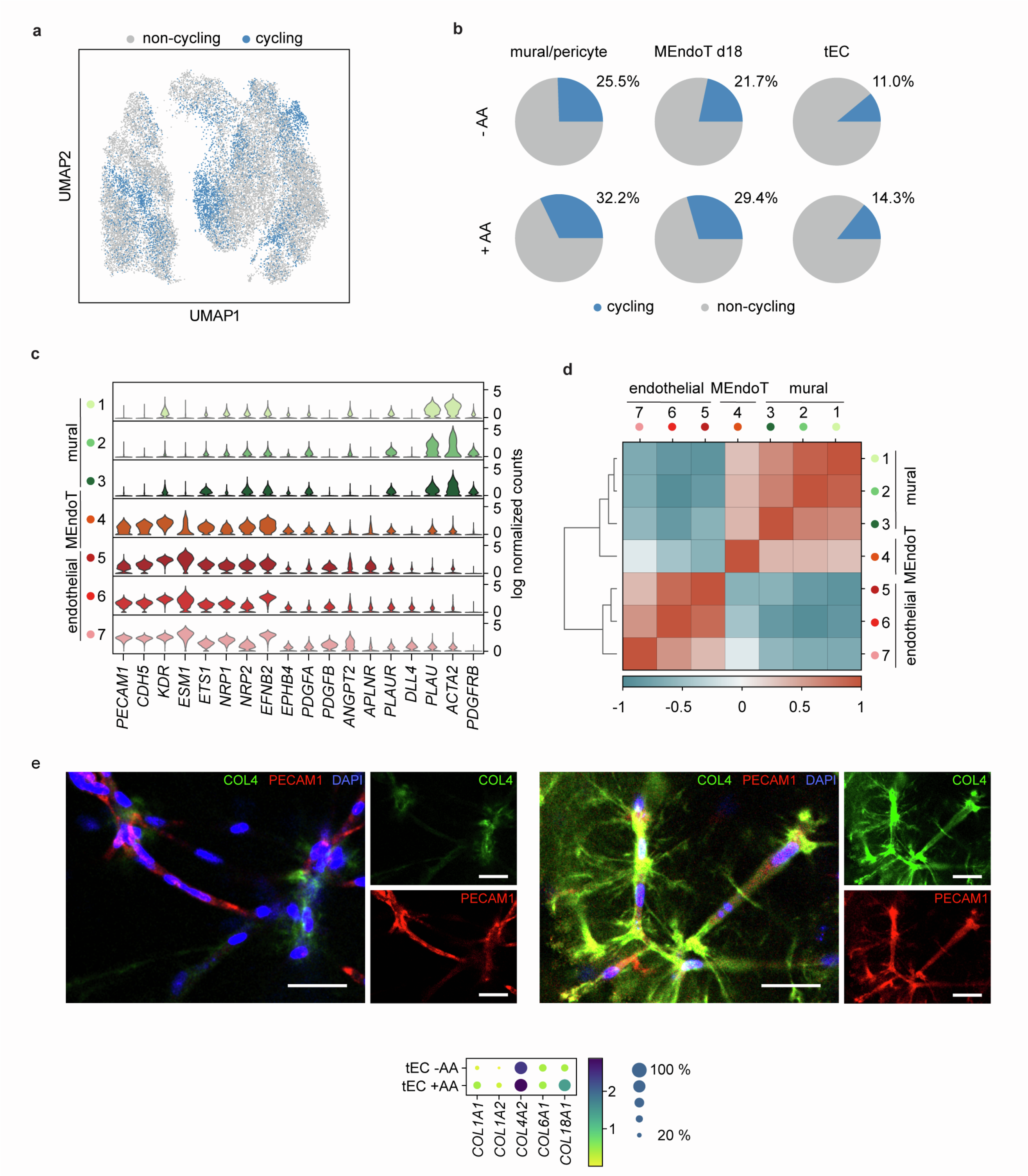

Figure S5. Single-cell transcriptomes and immunofluorescence analysis of stem-cell derived endothelial cells forms microvessels in Matrigel culture on day 18 of differentiation with and without ascorbic acid. a, UMAP plot of scRNA-transcriptomes acquired from cells in the Matrigel cultures on days 12 and 18. Blue colored dots represent cells expressing genes relating to S, G2, or M-phase. **b**, Pie charts show the percentage of cells undergoing the cell cycle within the respective clusters in the presence and

absence of ascorbic acid (AA). **c**, Violin plot of marker genes used to assign the cell cluster. The density distribution indicates the normalized cluster mean expression. **d**, Cell cluster Pearson correlation plot. **e, Left:** IF image of Matrigel culture on day 18 without and **right**: with AA addition to the media, where COL4, DAPI, and PECAM1 were counterstained. Scale: 20 μm. Dot plot of single-cell profile of selected collagen genes with ECM functionality in EC cultures with and without ascorbic acid. The color intensity and dot size denote the normalized cluster mean expression level and the fraction of cell expression for the corresponding gene, respectively. The figure corresponds to the main **Figure 5**.

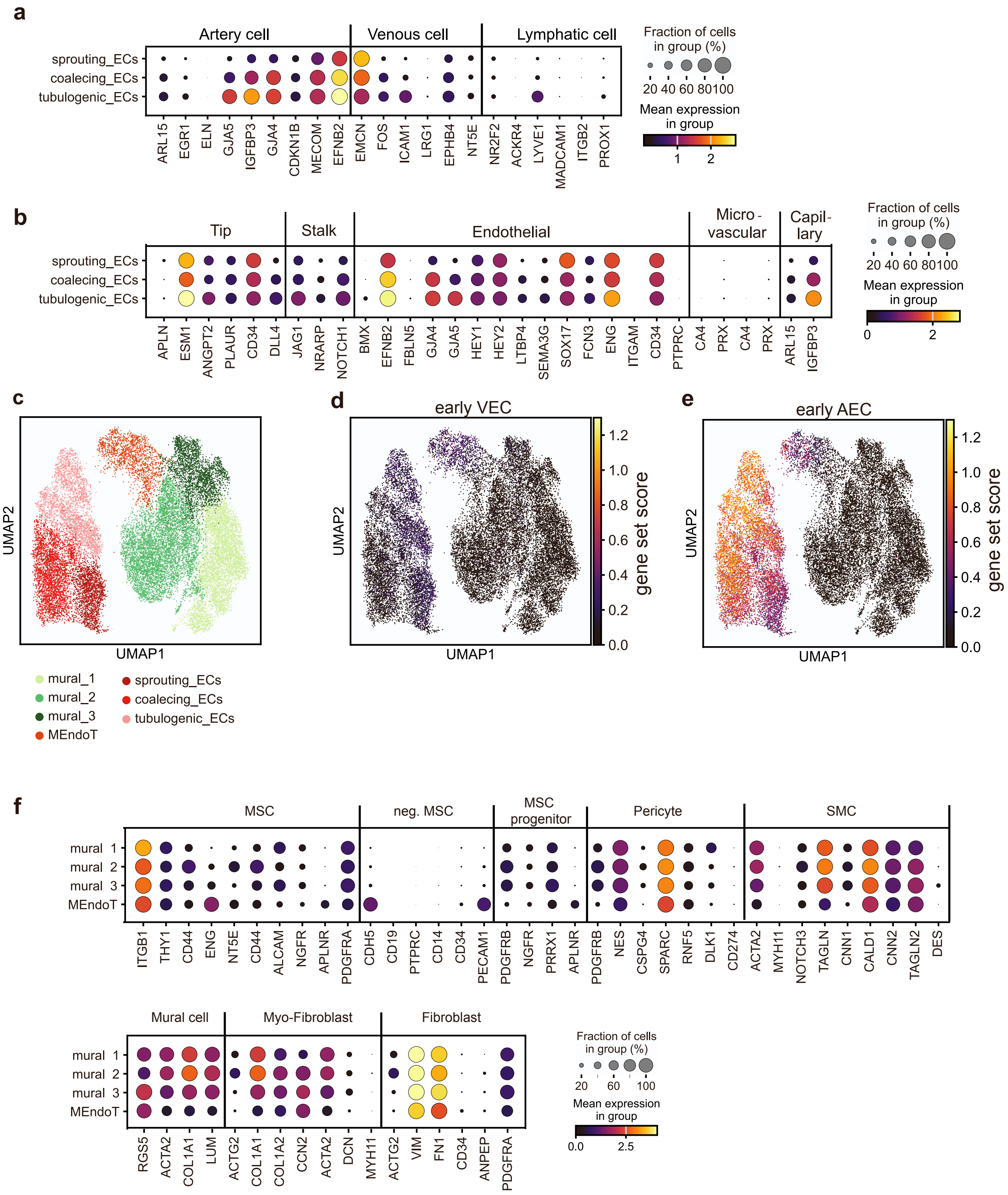

Figure 6. Standard cell type marker within the singe-cell transcriptomes of day 12 and 18 stem cell-derived hydrogel culture. a and **b**, Dot plots of mRNA expressions of literature curated cell type markers

in cells of the ECs clusters. **c**, UMAP plot of the single-cell transcriptomes as shown in Figure 5b of the main text. **d**, and **e**, Gene set enrichment analysis of the top 300 DEGs from single cell transcriptomes of early at the arterial, and venous ECs (Carnegie stage 10 and 11) within the cell clusters shown in **c**. Dot plots of mRNA expressions of literature curated cell type markers in the cells of the mural clusters. The figure corresponds to the main **Figure 5.**

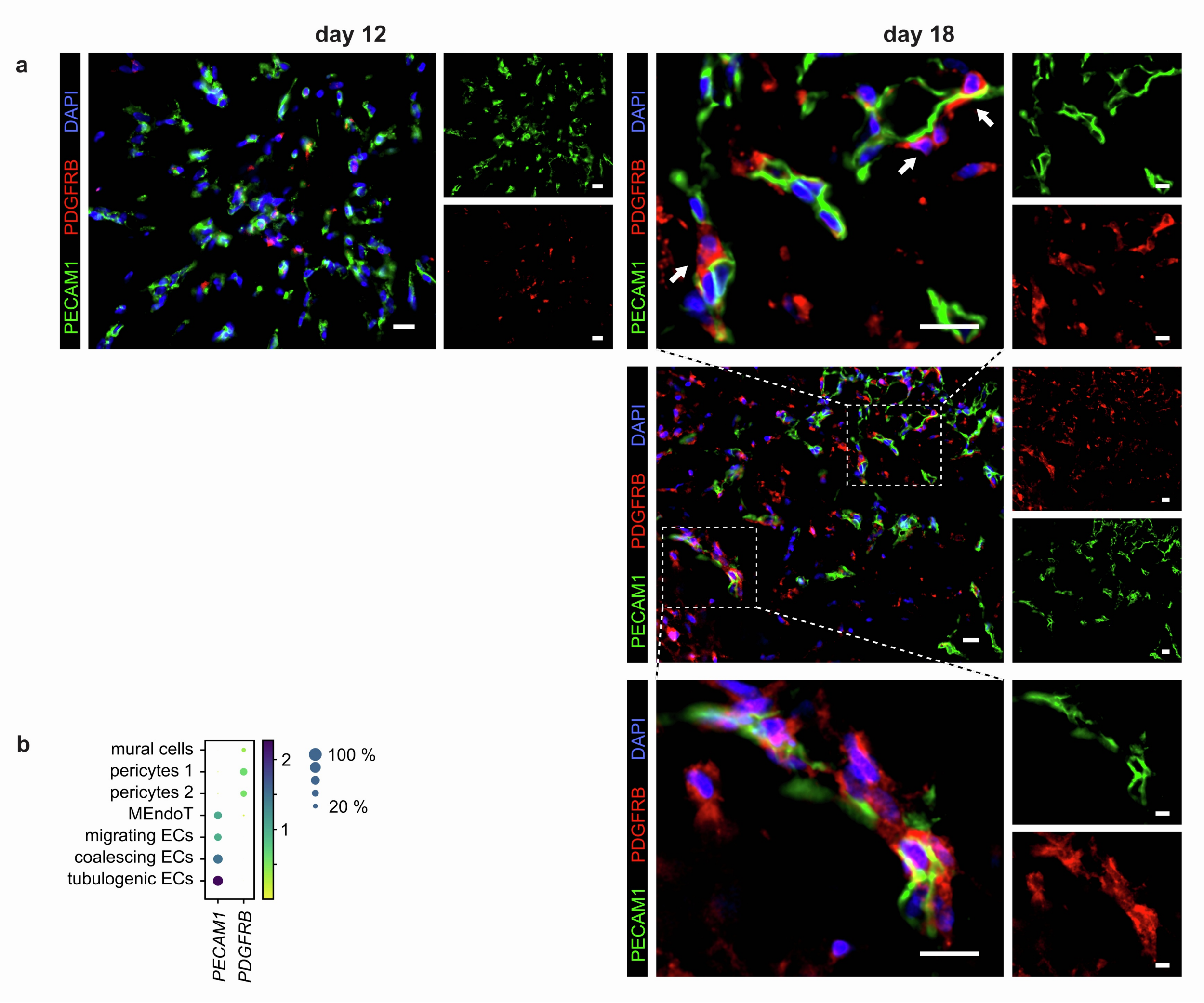

Figure S7. IF images of cryo-sections prepared from 3D hydrogel cultures on days 12 and 18 of differentiation. a, Location of mural cells to ECs is shown upon co-staining of PDGFRB and PECAM1, respectively. On day 18 (right panel) pericyte recruitment is highlighted by arrows. Scale: 20 µm. **b**, Dot plot of mRNA expression of genes whose corresponding protein staining is shown above. The color intensity and dot size denote the normalized cluster mean expression and the fraction of cells expressing the gene, respectively. *n*=4 images have been acquired for each condition. The figure corresponds to the main **Figure 6**.

Supplemental Tables

Table S1. Cell sample and sequencing statistics.

sample ID	condition	percentage of dead cells pre sequencing	total cell numbers pre quality filtering	mean number of genes per cell	mean number of sequencing read counts	number of analyzed cells after quality filtering	cell numbers with more than 10% mitochondrial genes	cell numbers with less than 2000 genes
day 0	3D suspension culture	17.4	8554	4440	22361	6411	811	1332
day 3	3D suspension culture	36.5	9621	4132	19660	4549	490	4582
day 6	3D suspension culture	19.1	6428	3644	12080	4807	345	1276
day 9_1	3D suspension culture	25	8154	4051	15200	6425	211	1517
day 9_2	3D suspension culture	30.8	7010	4388	19199	3011	804	3193
day 12	3D suspension culture	16.1	10551	5354	24817	4114	3161	3276
day 12	3D Matrigel	4.3	16097	3835	12165	13530	689	1876
day 18	3D Matrigel	12.3	11777	4807	19542	6462	2268	3047
day 18	3D Matrigel + ascorbic acid	7.6	14135	4673	19369	8743	2620	2772

The table corresponds to the main **Figure 1**.

Table S2 Culturing conditions of the differentiation in 2D and 3D.

	**day of differentiation**		**this study**	**McCracken et al.**	**day of differentiation**
**lateral mesoderm induction**	0-3	media	N2B27	N2B27	1-4
		BMP4	25 ng/mL	25 ng/mL	
		CHIR99021	7.5 µM	7 µM	
**endothelial induction**	3-7	media	StemPro-34	StemPro-34	4
		VEGF-A	200 ng/ml	200 ng/ml	
		Forskolin	2 µM	2 µM	
**maturation stage**	from 8 on	media	StemPro-34	EGM-2	until 8
		VEGF-A	30 ng/ml	50 ng/ml	
		FGF-2	30 ng/ml		
		human AB serum		1%	

The table corresponds to the main **Figure 1**.

Table S3. Cell type composition of the two culturing conditions on day 12 and day 18 (corresponds to Figure 4c and Figure 5c)**.**

**Day 12**	**d12 suspension**	**d12 MTG**	**d12 suspension / %**	**d12 MTG / %**
mural_1	1120	5339	34.7	47.2
mural_2	612	904	19	8
Endothelial progenitor	1175	42	36.4	0.4
coalescing_EC	221	2631	6.9	23.3
proliferating_EC	66	422	2	3.7
migrating_EC	30	1966	0.9	17.4

**Day 18**	**d12 MTG**	**d18 -AA**	**d18+AA**	**d12 MTG / %**	**d18 -AA / %**	**d18+AA / %**
mural_1	4950	197	104	44.3	3.5	1.4
mural_2/P1	198	1762	4528	1.8	31.2	59.8
mural_3/P2	980	816	949	8.8	14.4	12.5
MEndoT	13	779	850	0.1	13.8	11.2
migrating_ECs	1789	107	5	16	1.9	0.1
coalescing_ECs	3218	188	17	28.8	3.3	0.2
tubulogenic_ECs	31	1805	1116	0.3	31.9	14.7

The table corresponds to the main **Figure 5**.

Supplemental Experimental Procedures

Sample preparation for scRNA-seq

Matrix-free samples were washed with PBS, resuspended in Accutase, and incubated for cell detachment at 4°C for 30 min. The reaction was stopped by adding five volumes of media. Afterward, cells were washed once with PBS. Matrigel embedded samples were washed with PBS and incubated with 1 mg/mL Collagenase/Dispase solution (Sigma-Aldrich, Cat#10269638001) in StemPro-34 for around 4 h, until organoids detached from the Matrigel. The enzymatic reaction was stopped with 1 mL neutralisation buffer comprising 1%BSA, 1%P/S in DMEM:F12 (Sigma Aldrich, Cat#D6421). The single cells were cryo-preserved in DMEM with 10% heat-inactivated FBS (Thermo Fisher Scientific, Cat#A3160401) and 10% DMSO based on a previously described scRNA-seq sample preparation protocol (Guillaumet-Adkins et al., 2017). For sequencing, cryo-preserved cells were thawed in DMEM:F12. RNA libraries were generated using Chromium Single Cell 3' library and gel bead kit v3.1 (10x Genomics, Cat#1000079). The amplified cDNA library was sequenced on a NovaSeq 6000 S2 flow cell (Illumina, Cat#20028314). The sequenced cell numbers can be found in **Table S1**.

ScRNA-seq data pre-processing

Sequencing raw files were demultiplexed, aligned (reference genome hg38_ensrel97), filtered, barcodes and UMIs counted, and subjected to a quality filter with CellRanger (version 3.0.1, 10xGenomics). The pre-processing and downstream analysis was performed with the package 'Scanpy API' in python with default parameters, if not stated differently (Wolf et al., 2018). First, dead or stressed cells, identified by a percentage of mitochondrial genes higher than 10%, were filtered out. Next, cells with less than 200 and genes expressed in less than three cells were excluded. Afterward, the datasets of different days and experiments were concatenated, normalized to 10^4 gene counts per cell, and log-transformed. Batch effects were corrected using ComBat (Johnson et al., 2007). Further on, the highly variable genes were used for the downstream analysis. We corrected for the total gene counts, percentage of mitochondrial genes, and the cell cycle distribution of S, G2, and M phases to investigate differentiation-dependent changes on the transcriptome level (Luecken and Theis, 2019).

Dimensionality reduction, clustering, and cell-type annotation

The single cell nearest neighborhood graph was computed with the first 50 principal components and ten nearest neighbors. The cells were clustered with the Leiden algorithm with a resolution of 0.5. For visualization, the dimensionality of the data was reduced using Uniform Manifold Approximation and Projection (UMAP). For cell-type annotation, 300 DEGs for each of the clusters were calculated by ranking the clusters against all remaining cells with the t-test method (**Data File 1**). Clusters with proteasome-related genes scored at the top or a significantly reduced gene count were removed from the dataset as representing dying or damaged cells. The remaining clusters were annotated based on known marker genes.

RNA velocity through dynamical modeling

We analyzed the RNA velocity to investigate developmental trajectories by recovering directed dynamic gene information through splicing kinetics. Information like clustering and UMAP coordinates were retrieved from the Scanpy analysis. The pre-processing and downstream analysis was performed with scVelo using default parameters (Bergen et al., 2020). Splice variants and cells were filtered, normalized, and logarithmized with the function scv.pp.filter_and_normalize (parameters: min_cells=3, min_counts=200, min_shared_counts=20, n_top_genes=2000). The moments based on the connectivities were calculated with 40 PCAs and 10 neighbors in the next step. After recovering the dynamics, the latent time was calculated, and the velocity was calculated as a dynamical model.

Integration of datasets from different sequencing approaches

For integration and correction of datasets from different sequencing runs (**Figure 3**, **Figure S3c** and **d**), we applied bbknn to the datasets (neighbors_within_batch=40, n_pcs=10, trim=0, copy=True). We then re-clustered the cells with the Leiden algorithm at a resolution of 0.5 (Polański et al., 2020; Traag et al., 2018)).

Transcription factor enrichment analysis

The identified cluster-specific DDGs or DEGs (**Data File 1**) were entered in the ChEA3 web tool (Keenan et al., 2019) and the mean rank was plotted using R.

Enriched gene expression of gene sets

For the gene enrichment UMAP plots from Figure 1f, we used the data from Tyser et al. and converted the raw data to anndata file using scanpy. Cells were normalized to 10000 reads per cell and logarithmized. For enrichment scoring we used the scanpy command sc.tl.score_genes. The score function subtracts the average expression of a set of genes (top 300 DEGs of the clusters) with a reference gene set expression, randomly sampled from the whole gene pool.

Pathway and Gene Ontology enrichment

DEGs were filtered by their unique expression over all clusters (standard deviation above 0.5) and an expression value above 0.5. For the GO term enrichment, the R package enrichR was used with the "GO Biological Process 2018" database and plotted in R (Chen et al., 2013; Kuleshov et al., 2016).

CellphoneDB

The count matrix and cluster annotation were exported from scanpy, imported into R, and processed as recommended by the authors (Efremova et al., 2020). Cell-cell interactions were selected by the highest mean score and lowest p-value.

NicheNet

As Target gene input, the top 300 DEGs have been used. The calculation was done in R, converting the anndata element into a Seurat object. The process was performed as recommended by the authors (Browaeys et al., 2020).

Software specifications

The scRNA-seq alignment was run in CellRanger version 3.0.1 and the analyses were run in python 3.7.4 with Scanpy API version 1.4.4 or 1.5.1, anndata version 0.6.22 or 0.7.6, umap version 0.4.6, numpy version 1.17.4, scipy version 1.5.2, pandas version 1.1.3 or 1.0.5, scikit-learn version 0.22, statsmodels version 0.10.1, python-igraph version 0.7.1, scvelo version 0.2.1, matplotlib version 3.2.1, seaborn version 0.9.0, loompy version 3.0.6, XlsxWriter version 1.2.6, bbknn version 1.3.6 and scrublet version 0.2.1.

The plots of TFEA and GO term analysis were generated in RStudio with R version 3.6.0 with the usage of the R packages enrichR_3.0, ggpubr_0.4.0, ggplot2_3.3.3, stringr_1.4.0, EBImage_4.32.0, and bioimagetools_1.1.5.

NicheNet analysis was performed using following package versions: xlsx_0.6.5, ggpubr_0.4.0.999, cowplot_1.1.1, RColorBrewer_1.1-2, circlize_0.4.13, forcats_0.5.1, stringr_1.4.0, dplyr_1.0.7, purrr_0.3.4, readr_2.1.1, tidyr_1.1.4, tibble_3.1.0, ggplot2_3.3.5, tidyverse_1.3.1, SeuratObject_4.0.4, Seurat_4.0.2, nichenetr_1.0.0, gridBase_0.4-7, and ComplexHeatmap_2.6.2

Fluorescence-activated cell sorting (FACS)

Harvested cells were washed three times with PBS (200 x g for 5 minutes) and singularized using Accutase. Five volumes of FACS buffer (10% FBS in PBS) were added and centrifuged at 300 x g for 5 minutes. Blocking was done for 20 minutes with StemPro-34 plus 10 % FBS on ice. The cell suspension was filtered using a 40 µm nylon cell strainer (Corning, Cat#431750). Live dead staining was performed with Trypan

Blue (Fisher Scientific, Cat#15393661) while cells were counted. 20 µL of the antibody FITC anti-Human CD31 (BD Pharmingen, Cat#555445) and PE anti-Human CD140b (BD Pharmingen, Cat#558821), and 1 µL of violet, fluorescent reactive dye (Invitrogen, Cat# L34963A) were added per 10^6 cells and incubated for 30 minutes at room temperature (RT). After washing once with the FACS buffer, cells were resuspended in Tyto buffer (10^6 cells/mL, MACSQuant Tyto (TM) Running Buffer (Miltenyi Biotech, Cat#130-107-207). The sorting was performed on a MACSQuant Tyto. For isotype controls the FITC mouse IgG1 κ isotype control (BD Pharmingen, Cat#554679) and PE mouse IgG2a κ Isotype Control (BD Pharmingen, Cat#559319) were used.

Flow cytometry

Singularized cells were washed once with PBS (centrifugation conditions: 300 x g, 5 min). Cells were fixated at RT for 15 minutes with 4% PFA (LifeTechnologies, Cat#28908) in PBS and afterward washed twice with FACS buffer. 10^5 cells were transferred into a U-bottom-shaped 96-well plate (Greiner, 650101) and an antibody (2 µL per 15^5 cells) diluted in 100 µL FACS buffer was applied per well. After incubation for 30 minutes at RT, cells were washed twice with FACS buffer. For the measurement, the pellet was resuspended in 200 µL of FACS buffer and transferred through a cell strainer into 5mL round-bottom FACS-tubes (Coring, Cat#352052). Flow cytometry analysis was performed on a MACSQuant VYB.

Cryo-embedding

For embedding of 3D suspension culture aggregates, 500 µL of 4% PFA were added and incubated for 15 minutes on ice. The disc was detached from the walls with a needle. After two washing steps with PBS, an incubation with, first, 10%, second, 30% sucrose at RT for 2 h and, third, with a 1:1 mixture of 30% sucrose and OCT medium (Carl Roth, 6478.1) at 4°C overnight. All incubation steps were implemented on a wave shaker at RT. The medium was replaced by pure OCT and the sample was frozen on dry ice. The slicing was performed on a Leica CM1860 cryostat, where the thickness was adjusted to 20 µm. We used the following antibodies to prepare the immunofluorescence stainings: PECAM1 (Thermofisher, Cat#AbWM59), PDGFRB (Thermofisher, Cat#AbAPB5), DLL4 (Cell Signaling, Cat#mAb 96406), DEPTOR (Cell Signaling, Cat#mAB 11816), CLDN5 (Thermofisher, Cat#MA5-32614), ICAM2 (Cell Signaling, Cat#mAB 13355), Collagen IV (Abcam, Cat#ab6586), anti-mouse Alexa 488 (Thermofisher, Cat#A21202), and anti-rabbit Alexa 555 (Invitrogen, Cat#A31572).

Cell migration assay

A fluorescently labeled iPSC line (Corell Institute, Cat# AICS-0036-028) was differentiated according to the above reported 3D differentiation protocol. On day 6 of differentiation, endothelial progenitors were FACS sorted for $PECAM1^+$ (Becton Dickinson, Cat#555455) and then cultivated in EGM2 media (Angio-Proteomie, Cat#cAP-02) with supplements (EGM2) in 6-wells following the given procedure for 2D cell cultures. Sorted endothelial progenitors were two times passaged before use. The cell migration assay was set up on a commercially available microfluidic chip from Mimetas (Mimetas, OrganoPlate 3-lane 40 Cat#4004-400-B). For handling the chip platform, we followed the vendor's instructions. The chip comprises three converging microfluidic channels with inlet and outlet ports. Upon filling the middle channel with a 2.5 mg/ml neutralized Collagen-1 (Corning, Cat#354236) hydrogel solution solved in PBS, the two outer channels were separated by a diffusion barrier. After gelation at 37°C for 30 min the inlet port of the gel chamber was filled with Hanks' Balanced Salt solution (Gibco, Cat#14175095) to prevent drying out of the collagen solution. Then, the two outer microchannels were filled with EGM2 supplemented with half the volume of a given growth factor supplement mix solution (EGM2-s/2) to reduce the internal growth factor contribution to the assay. For cell seeding on the chip, the endothelial progenitors were retrieved from the cell culture plate with Accutase (Sigma Aldrich, Cat#A6964) treatment for 5 minutes. After two times washing SC-ECs with EGM2-s/2, the cells were pipette into one of the microchannel inlet ports with a

concentration of 15.000 cells/ml. 30 minutes after seeding the inlet ports were filled up with 50 µL EGM2-s/2 and placed on the rock shaker (tilt angle 7° with an inverting interval of 8 minutes) to induce flow conditions. After SC-EC reached confluency between 30% and below 80% within the microfluidic channel the ligands were added to the inlet port of the microchannel opposing the microchannel with the cells. The cell confluency was measured as described below. Fluorescence images of the hydrogel chamber were taken before and 24h after ligand addition. All ligands had a concentration of 10ng/mL, except for angiopoietin and CRX12 which had a concentration of 100ng/mL. The increase of the cell confluency within the hydrogel area or seeding channel of the chip platform was obtained by first cropping the same region of interest (ROI) from all fluorescence images. Cropped images were converted into binary images by using a constant global threshold value. Cell occupancy areas in the binary images were determined with the measure function of ImageJ.

References

Bergen, V., Lange, M., Peidli, S., Wolf, F.A., and Theis, F.J. (2020). Generalizing RNA velocity to transient cell states through dynamical modeling. Nature Biotechnology 2020 38:12 *38*, 1408–1414. https://doi.org/10.1038/s41587-020-0591-3.

Browaeys, R., Saelens, W., and Saeys, Y. (2020). NicheNet: modeling intercellular communication by linking ligands to target genes. Nature Methods *17*, 159–162. https://doi.org/10.1038/s41592-019-0667-5.

Chen, E.Y., Tan, C.M., Kou, Y., Duan, Q., Wang, Z., Meirelles, G.V., Clark, N.R., and Ma'ayan, A. (2013). Enrichr: Interactive and collaborative HTML5 gene list enrichment analysis tool. BMC Bioinformatics *14*, 128. https://doi.org/10.1186/1471-2105-14-128.

Efremova, M., Vento-Tormo, M., Teichmann, S.A., and Vento-Tormo, R. (2020). CellPhoneDB: inferring cell–cell communication from combined expression of multi-subunit ligand–receptor complexes. Nature Protocols *15*, 1484–1506. https://doi.org/10.1038/s41596-020-0292-x.

Johnson, W.E., Li, C., and Rabinovic, A. (2007). Adjusting batch effects in microarray expression data using empirical Bayes methods. Biostatistics *8*, 118–127. https://doi.org/10.1093/biostatistics/kxj037.

Keenan, A.B., Torre, D., Lachmann, A., Leong, A.K., Wojciechowicz, M.L., Utti, V., Jagodnik, K.M., Kropiwnicki, E., Wang, Z., and Ma'ayan, A. (2019). ChEA3: transcription factor enrichment analysis by orthogonal omics integration. Nucleic Acids Research *47*, W212–W224. https://doi.org/10.1093/nar/gkz446.

Kuleshov, M.V., Jones, M.R., Rouillard, A.D., Fernandez, N.F., Duan, Q., Wang, Z., Koplev, S., Jenkins, S.L., Jagodnik, K.M., Lachmann, A., et al. (2016). Enrichr: a comprehensive gene set enrichment analysis web server 2016 update. Nucleic Acids Research *44*, W90–W97. https://doi.org/10.1093/nar/gkw377.

Luecken, M.D., and Theis, F.J. (2019). Current best practices in single-cell RNA-seq analysis: a tutorial. Molecular Systems Biology *15*. https://doi.org/10.15252/msb.20188746.

Polański, K., Young, M.D., Miao, Z., Meyer, K.B., Teichmann, S.A., and Park, J.E. (2020). BBKNN: fast batch alignment of single cell transcriptomes. Bioinformatics *36*, 964–965. https://doi.org/10.1093/bioinformatics/btz625.

Traag, V.A., Waltman, L., and Eck, N.J. van (2018). From Louvain to Leiden: guaranteeing well-connected communities. Sci. Reports *9*, 5233. https://doi.org/10.1038/s41598-019-41695-z.

Wolf, F.A., Angerer, P., and Theis, F.J. (2018). SCANPY: Large-scale single-cell gene expression data analysis. Genome Biology *19*, 15. https://doi.org/10.1186/s13059-017-1382-0.
