# Supplementary material for: Single-cell characterization of neovascularization using hiPSC-derived endothelial cells in a 3D microenvironment
Source: Stem Cell Reports. 2023 Sep 14;18(10):1972–86. doi: 10.1016/j.stemcr.2023.08.008 (PMC10656300; doi:10.1016/j.stemcr.2023.08.008)
Supplement: Document S2. Article plus supplemental information [file mmc3.pdf]

Stem Cell Reports
Resource
OPEN ACCESS

Single-cell characterization of neovascularization using hiPSC-derived endothelial cells in a 3D microenvironment

Simon Rosowski,[1] Caroline Remmert,[1] Maren Marder,[1] Misao Akishiba,[1] Judith Bushe,[2] Annette Feuchtinger,[2] Alina Platen,[1] Siegfried Ussar,[3,5] Fabian Theis,[4,6] Sandra Wiedenmann,[1,*] and Matthias Meier[1,7,*]
[1]Helmholtz Pioneer Campus, Helmholtz Zentrum München, Neuherberg, Germany
[2]Research Unit Analytical Pathology, Helmholtz München, 85764 Neuherberg, Germany
[3]Institute for Diabetes and Obesity, Helmholtz Diabetes Center, Helmholtz Zentrum München, Neuherberg, Germany
[4]Institute of Computational Biology, Helmholtz Zentrum München, Neuherberg, Germany
[5]German Center for Diabetes Research (DZD), 85764 Neuherberg, Germany
[6]Department of Mathematics, Technical University of Munich, 85748 Garching bei München, Germany
[7]University Leipzig, Center for Biotechnology and Biomedicine, Institute of Biochemistry, Leipzig, Germany
*Correspondence: sandra.wiedenmann@helmholtz-muenchen.de (S.W.), matthias.meier@helmholtz-muenchen.de (M.M.)
https://doi.org/10.1016/j.stemcr.2023.08.008

SUMMARY

The formation of vascular structures is fundamental for *in vitro* tissue engineering. Vascularization can enable the nutrient supply within larger structures and increase transplantation efficiency. We differentiated human induced pluripotent stem cells toward endothelial cells in 3D suspension culture. To investigate *in vitro* neovascularization and various 3D microenvironmental approaches, we designed a comprehensive single-cell transcriptomic study. Time-resolved single-cell transcriptomics of the endothelial and co-evolving mural cells gave insights into cell type development, stability, and plasticity. Transfer to a 3D hydrogel microenvironment induced neovascularization and facilitated tracing of migrating, coalescing, and tubulogenic endothelial cell states. During maturation, we monitored two pericyte subtypes evolving mural cells. Profiling cell-cell interactions between pericytes and endothelial cells revealed angiogenic signals during tubulogenesis. *In silico* discovered ligands were tested for their capability to attract endothelial cells. Our data, analyses, and results provide an *in vitro* roadmap to guide vascularization in future tissue engineering.

INTRODUCTION

Endothelial cells (ECs) form the inner luminal epithelium of vascular structures, including arteries, veins, and lymphatic vessels (Ricard et al., 2021). Apart from core nutrient and oxygen transport functions, the vascular system enables immune cell trafficking, vasomotor tone, and wound healing (Chatterjee, 2021). To fulfill the different homeostatic functions, ECs exhibit a high degree of plasticity to form differently sized fenestrae and branched vessel structures, recruit organ-specific scaffold cells, or remodel the extracellular matrix composition of the vascular bed (Dejana et al., 2017; Holm et al., 2018). The developmental factors determining EC formation, maturation, and specification are not fully revealed yet.

In vitro cell culture systems with patient and stem cell-derived ECs have become indispensable for vascular research to overcome the paucity of longitudinal studies in patients, reducing the biological complexity. In particular, stem cell-derived ECs are of interest because they provide access to the early development stage of vascular structures involving cell type formation and specification (Nguyen et al., 2021). *In vivo*, ECs evolve from mesoderm-derived progenitors (angioblasts) in response to FGF2 and VEGFA signals from the adjacent visceral endoderm (Poole et al., 2001). Stem cell differentiation protocols recapitulate *in vivo* development by first inducing mesodermal progenitor cells, with BMP4 and GSK3β inhibitors (Tan et al., 2013), and subsequently ECs, by adding VEGFA (Chen et al., 2011). Several variations of the differentiation protocols have been reported (Xu et al., 2019), where the differences between the resulting ECs remain unresolved. Stem cell-derived ECs do not show a direct correlation with any organ-specific ECs, and it remains unclear which developmental stage they reflect (Paik et al., 2020). To closely resemble the *in vivo* microenvironment in a dish, 3D stem cell culture formats were introduced, which enable stem cell-derived ECs to form tubes (Olmer et al., 2018). Moreover, stem cell-derived ECs can fully self-assemble into blood vessels in an organoid-like format, including mural cells, i.e., smooth muscle and pericytes lining the outer surface of the vessel endothelium (Wimmer et al., 2019). In all culture formats, mural cells co-evolve during EC differentiation (McCracken et al., 2020), which implies inherent strong cell-cell communication during differentiation and maturation.

During embryonic development, immature ECs coalesce and undergo tube formation to produce a vascular plexus (Noden, 1989), which further differentiates into the specific vessel types. Single-cell transcriptomic analysis of the *in vivo* neovascularization process was performed by extracting cells from laser-induced choroid lesions in mice (Rohlenova

This is an open access article under the CC BY license (http://creativecommons.org/licenses/by/4.0/).

et al., 2020). The data depicted a highly heterogeneous process with multiple transcriptomic EC stages and types, including phalanx and tip cells, with associated distinctive metabolic profiles. Comparable single-cell transcriptomic analyses in a less complex *in vitro* microenvironment with stem cell-derived ECs is not available but would add fundamental knowledge on the formation of vascular structures.

In this study, we used single-cell transcriptomics to sequentially investigate the development and neovascularization of human induced pluripotent stem cells (hiPSCs) in an *in vitro* 3D microenvironment. In the first step, we used single-cell transcriptomics to explore the differentiation trajectory of co-evolving ECs and mural cells in a 3D suspension cell culture format. Comparison of single-cell transcriptomics of ECs, evolved from a monolayer and a 3D suspension culture, revealed differences in ECM gene expression and optimal differentiation parameters. In the second step, the single-cell transcriptomics approach was used to analyze neovascularization in the heterogeneous 3D suspension culture upon transfer into a hydrogel culture. Ligand-receptor links between ECs and subcellular pericyte populations were predicted from the single-cell transcriptomes during vessel maturation. To prove functional importance of the *in silico*-discovered ligands, we then tested for their potential to attract ECs in an *in vitro* migration assay. Our data and analysis provide the resources for future EC specification studies and tissue engineering approaches.

RESULTS

Single-cell analysis of endothelial differentiation in a 3D suspension culture

To investigate the differentiation of hiPSCs into ECs in a 3D cell culture format at the single-cell level, we adopted the chemical two-step induction protocol (Olmer et al., 2018; Patsch et al., 2015; Wimmer et al., 2019). Therefore, hiPSCs were differentiated toward the mesoderm germ layer, and EC development was induced in the second step (Figure 1A). In suspension, the 3D cell cultures were stable throughout the differentiation and grew from a diameter of 150 to 300 μm. While hiPSC-derived aggregates exhibited a uniform spheroidal shape, aggregates from day 4 showed a more prolate shape. On day 9, 33.2% of the cells expressed the endothelial marker CD31 (PECAM1), with a standard variation of 5.3% over three biological repeats and two patient cell lines (Figures S1A and S1B). To reconstruct EC development in the 3D suspension culture and define time-resolved cell composition, we performed single-cell mRNA sequencing (scRNA-seq) analysis on 22,192 cells (see Table S1). Upon dimensional reduction (McInnes et al., 2018) and Leiden clustering (Traag et al., 2018), the cells were assigned into five clusters. With the progression of the differentiation process, the recorded single-cell transcriptomes changed, as indicated by the time-dependent emergence of distinct cell clusters (Figure 1B). All cell clusters could be assigned to cell types by matching known mesodermal and endothelial developmental markers to the differentially expressed genes (DEGs) in the respective cluster (Figure 1C). The cell populations were assigned to pluripotent stem cells (cluster 1), mesodermal cells (cluster 2), mural cells (cluster 3), endothelial progenitor cells (cluster 4), and endoderm (cluster 5). At the start of differentiation (day 0), the cell population consisted of homogeneous undifferentiated hiPSCs, where over 96% of the cells expressed the pluripotency markers *OCT4*, *SOX2*, and *NANOG*. Cells assigned as mesodermal cells appeared on day 3 of differentiation and expressed markers for the lateral plate mesoderm, including *HAND1*, *MESP1*, and *APLNR*. Mural cells observed on day 6 of differentiation showed reduced *HAND1* expression level, while the smooth muscle marker *ACTA2*, pericyte marker *PDGFRB*, and mesenchymal marker *COL1A1* were consistently expressed. Only a small fraction (0.9%) of endoderm cells was observed (Figure 1D). Immunohistochemical staining of 3D suspension cell cultures at day 9 of differentiation with the markers PECAM1 and PDFGRB showed de-mixing of the two cell populations; however, there was no induction of vessel formation, supporting the endothelial progenitor cell state (Figure S2A). Evaluation of cell-cycle states showed that endothelial progenitor cells were entirely in the G1 phase, whereas approximately 50% of the mural cells were in G2 and S and thus proliferating (Figure S2B). This explains the increasing proportion of mural cells from days 6 to 9 in the 3D cell culture. To test the robustness of the differentiation approach at the single-cell level, we sequenced the cells from day 9 of two independent differentiation experiments. In both cases, a bimodal distribution of endothelial progenitor cells and mural cells was observed, with comparable distribution numbers (Figure S2C).

To further confirm the cluster assignments, we calculated a gene expression enrichment score of the top 300 DEGs of each cell cluster within a single-cell dataset of the human embryo at the gastrulation phase (Tyser et al., 2021) (Figures 1E and 1F). Expectedly, the DEGs of the pluripotent stem cells were highly enriched in human epiblast cells. The *in vitro*-differentiated mesodermal cell on day 3 correlated broadly with nascent, emergent, and advancing mesoderm cells of the human gastrulating embryo. Strikingly, within the top 300 DEGs of the endothelial progenitors, genes of the hemogenic ECs are significantly enriched. Gene enrichment factors for the co-evolving mural cells were highest with advancing mesoderm and extraembryonic mesoderm cells. In summary, the gene enrichment analysis showed that the occurring cell types

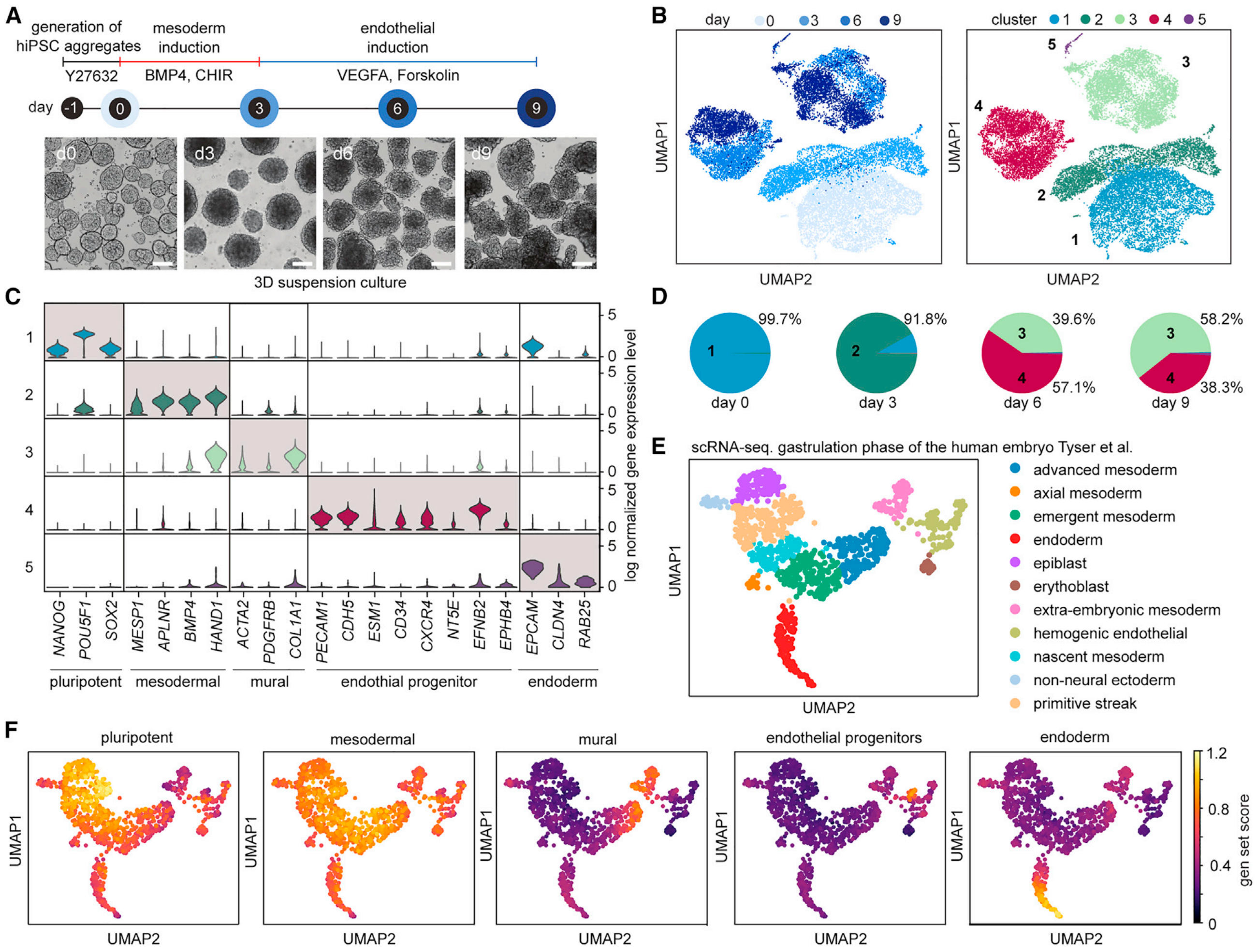

Figure 1. Single-cell transcriptomics reveals the differentiation trajectory of hiPSCs into endothelial cells in 3D suspension culture

(A) Schematic of the endothelial differentiation timeline with sampling points and chemical induction protocol. Bright-field images show representative 3D suspension cultures at the corresponding time point. Scale bar denotes 100 μm.

(B) UMAP plot of the single-cell transcriptomes. Left: light to dark blue denotes the time points of sampling. Right: five unique cell clusters were identified during the endothelial differentiation: (1) pluripotent cells, (2) mesodermal cells, (3) mural cells, (4) endothelial progenitor cells, and (5) epithelial cells.

(C) Violin plot shows the cluster expression levels of differentially expressed genes for the six cell clusters and the commonly used cell markers for cell-type assignment.

(D) Cell-type distribution analysis along the differentiation trajectory.

(E) Single-cell transcriptomic data with cell-type assignment of the human embryo at the gastrulation phase adopted from Tyser et al. (2021).

(F) Gene set enrichment analysis of the top 300 DEGs of each cell cluster found in (B) within the cell clusters of the human embryo at the gastrulation phase shown in (E).

of the *in vitro* differentiation resemble the cell types found during the mesodermal-to-hemogenic EC development of the human embryo during gastrulation.

Time trajectory of endothelial development

To resolve the time-dependent relationships of the cell clusters, we performed a dynamic RNA velocity analysis and identified the dynamic driver genes (DDGs) in endothelial differentiation. For this, we first calculated the latent time based on the balance of spliced and unspliced RNA transcripts within the single-cell transcriptomes (Figure 2A). The corresponding RNA velocity is weakly streamlined between the transition states of the cell clusters. Nevertheless, a partition-based graph abstraction (PAGA)

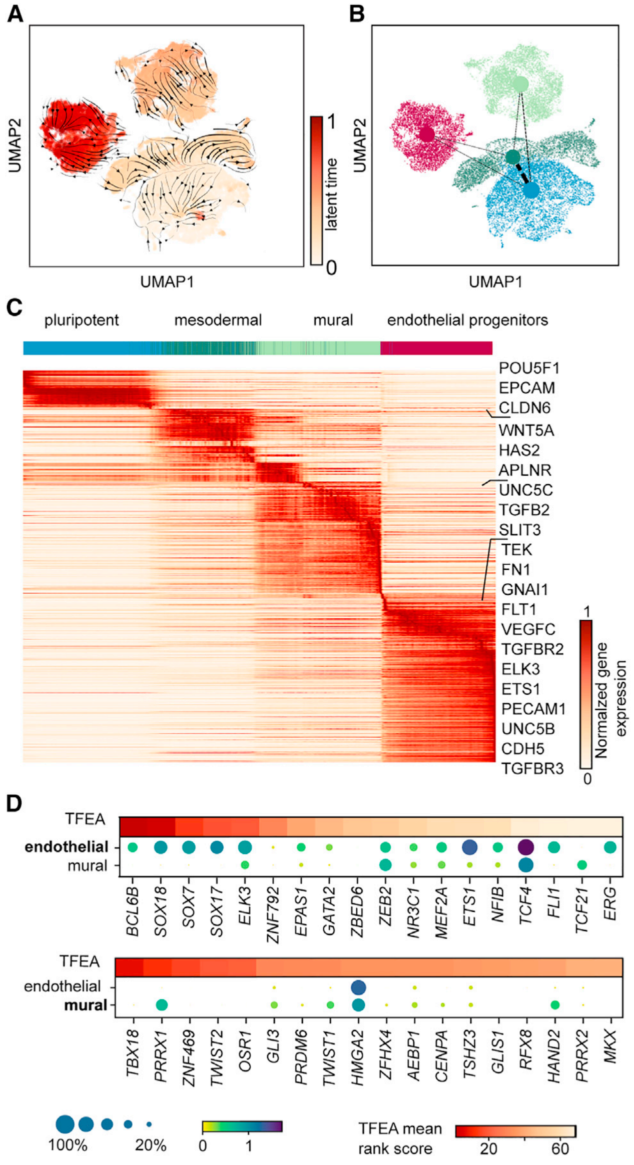

Figure 2. Transcriptomic dynamics predict the differentiation path for endothelial and mural cells

(A) UMAP plot colored with the latent time calculated based on RNA velocity analysis. The velocity streamlines are given by the black arrows.

(B) UMAP plot colored by annotated cell-type colors and with an overlay of PAGA connectivity.

(C) Heatmap of the cluster-specific DDGs sorted according to their likelihood scores and latent time (see Table S4).

(D) TFEA on the cluster-specific and unique DDGs for endothelial progenitor cells and mural cells. TFEA scores are represented in a color code, whereas the mean expression levels of the corresponding TFs are shown as dot plots. The color intensity and dot size denote the normalized cluster mean expression and the fraction of cells expressing the gene, respectively.

analysis on the RNA velocity demonstrated the connectivity (Figure 2B) between the endothelial progenitor and mural cells and the mesodermal progenitors. Subsequently, we plotted the DDGs along the velocity latent time, where mural and endothelial progenitor cells were defined as end states, to trace the central genes for the development of the respective cell types in 3D (Figure 2C). Similar to the DEG analysis, cluster-specific DDGs were identified. The list of DDGs for each cell cluster is given in Table S4. The top DDGs for the mural cell progenitors were involved in cell migration, attraction, or repulsion (*UNC5C*, *SLIT3*, and *TGFB2*). For ECs, known developmental genes of vasculogenesis were upregulated, including the *VEGF* receptors (*KDR*, *FLT1*) and the interacting receptors *TIE1* and *TEK*. To infer the transcription factors (TFs) controlling the development of endothelial progenitor cells and mural cell progenitors, a TF enrichment analysis (TFEA) was performed on the DDGs (Figure 2D). The highest-ranked TFs for EC development were *BCL6B*, *ETS1*, *ELK3*, and *ERG*. All of them are reportedly associated with the process of early vasculogenesis with context-dependent function but integrate the VEGF and Notch signaling pathways. Notably, the extracted TFs are putatively responsible for the development of the two identified cell types, but not for neovascularization, due to the missing vessel organization within the 3D cell cultures at days 6 and 9 of differentiation. TFEA of the DDGs for the mural cell progenitors revealed *TBX18*, *CENPA*, and *HAND2* as the top regulatory TFs (Osterwalder et al., 2014). Expression of *TBX18* was not detected in the scRNA dataset; however, its transcriptional activity matched with its recently identified expression pattern in mouse pericytes and vascular smooth muscle cells of the retina, brain, heart, skeletal muscle, and adipose fat depots (Wu et al., 2013). It must be considered that the velocity analysis with spare time points has to be understood as transcriptional correlation rather than real dynamics. Nevertheless, the turn on and off of genes derived from the velocity analysis match with the expectation, for example, for the cell-cycle regulator *HMGA2*. In mural cells, *HMGA2* is in the turned-on state, whereas in endothelial progenitor cells it is in the turned-off state but still detectable.

Comparison of endothelial differentiation in 3D versus 2D cell culture formats

In the next step, we sought to compare the *in vitro* development of ECs in 3D suspension culture with a previously performed stem cell differentiation approach with an adherent 2D cell culture on the single-cell level (McCracken et al., 2020). Chemical compounds for the endothelial induction protocol were the same; however, minor concentration differences of the individual compounds existed (Table S2).

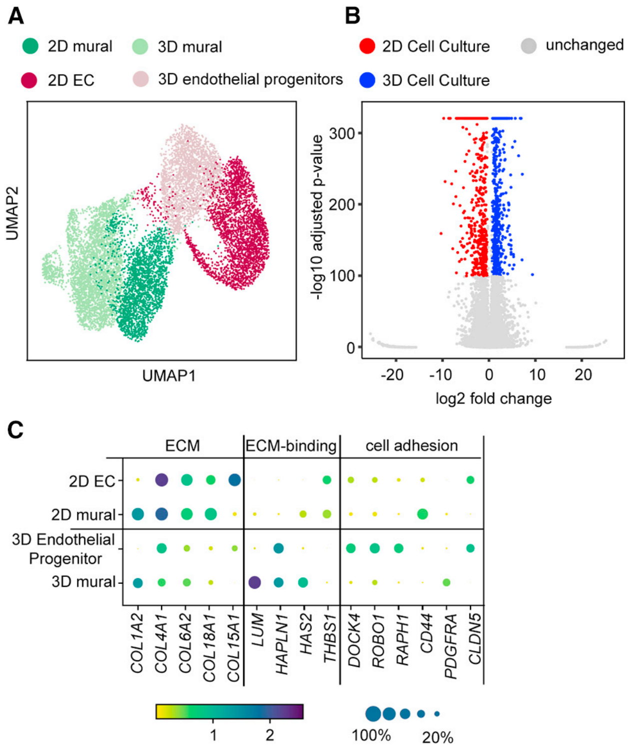

Figure 3. Comparison of EC differentiation in 2D and 3D cell culture formats

(A) UMAP plot of single-cell transcriptomes of iPSC-derived endothelial cells differentiated on a 2D adhesion monolayer (5,267 cells) and as 3D suspension culture (6,492 cells).

(B) Volcano plot representing genes differentially expressed between ECs differentiated in 2D and 3D cell cultures.

(C) Dot plot of representative DEGs with assigned cellular functions and biological processes. The color intensity and dot size denote the normalized cluster mean expression and fraction of cells expressing the corresponding gene, respectively.

Single-cell transcriptomes of mural cells and ECs differentiated to day 9 in a 3D cell culture showed a different marker expression profile than those differentiated to day 8 in a 2D cell culture (Figure 3A). This led to the result of two separate Leiden clusters for the cell types cultured in 2D and 3D in the UMAP plot. To quantify the differences, we combined the two scRNA datasets. Of the 14,383 genes, 575 and 608 showed expression-level differences with a p value lower than 10^{-100} in the 2D and 3D cell cultures, respectively (Figure 3B). Dominantly, the expression patterns of the extracellular matrix (ECM) genes were distinct in different culture formats. Within the 2D cell culture format, ECs exhibited a strong collagen phenotype with high expression levels of basal lamina proteins, such as COL4A1/2, COL6A2, or COL18A1 (Figure 3C). In 3D cell culture, ECs expressed hyaluronic acid and the corresponding binding proteins. In contrast, endothelial progenitor cells within the 3D suspension culture upregulated cell-cell interaction and actin remodeling genes, such as the Wnt signaling proteins CLD5, DOCK4, and CTNNB1 and the Rap1 signaling proteins RAP1B, RAPGEF5, and RASIP1.

Within the 3D cell culture endothelial progenitor cells stop proliferating from day 6 of differentiation, whereas stem cell-derived ECs in the 2D cell culture format proliferated as indicated by the combined scRNA data (Figure S2D).

This raised the question of whether the separation of the ECs from the mural cells or the transfer to a 2D culture format reinstates the proliferation of ECs. Fluorescence-activated cell sorting (FACS)-sorted PECAM1^{+} ECs from the 3D suspension culture proliferated and could be passaged over six generations upon plating on a culture dish, but also endothelial progenitors plated with the mural cells could be passaged without reduction of the endothelial progenitor fraction (Figure S1C), demonstrating that growth arrest is associated with the 3D suspension culture format. A further important question was whether sorted ECs and mural cells maintained cell-type stability after transfer into the 2D cell culture format. To evaluate this aspect, we investigated the ratio of PECAM1^{+} and PDGFRB^{+} cells in 2D cell cultures prepared from sorted and unsorted 3D aggregates at day 6 of differentiation. Within sorted PECAM1^{+}/PDGFRB^{-} cell cultures, no reappearance of PDGFRB^{+} cells could be detected after 6 days of culturing in a monolayer culture format under EC differentiation medium (Figure S1D). In contrast, PECAM1^{-}/PDGFRB^{+}-sorted cells lost the PDGFRB expression under the same conditions. Upon plating of the unsorted 3D cell cultures onto a monolayer on day 6, the fraction of PECAM1^{+} cells increased from 64% to 72% after 6 days of culture, whereas the fraction of PDGFRB^{+} cells decreased from 18% to 10% simultaneously. In comparison, in the 3D suspension culture, the fraction of PECAM1^{+} cells decreased, while the fraction of PDGFRB^{+} increased within the same culture interval as seen by the scRNA analysis. This suggests that cell-cell signaling between endothelial progenitor and mural cells within the 3D culture format leads to changed proliferative behavior compared with the 2D cell culture format.

Single-cell transcriptomics of neovascularization

After embedding of the stem cell-derived endothelial progenitor cells from the 3D suspension culture into Matrigel, sprouting was induced within the first 12 h (Figures 4A and S3A). Single-cell transcriptomes of the sprouting Matrigel culture were determined 48 h after transfer and compared with single-cell transcriptomes of cells within 3D suspension cultures kept to the same day of differentiation. Three distinct transcriptomic EC subclusters were detected within the Matrigel culture (clusters 4–6), which separated from the endothelial progenitor cells in the 3D suspension

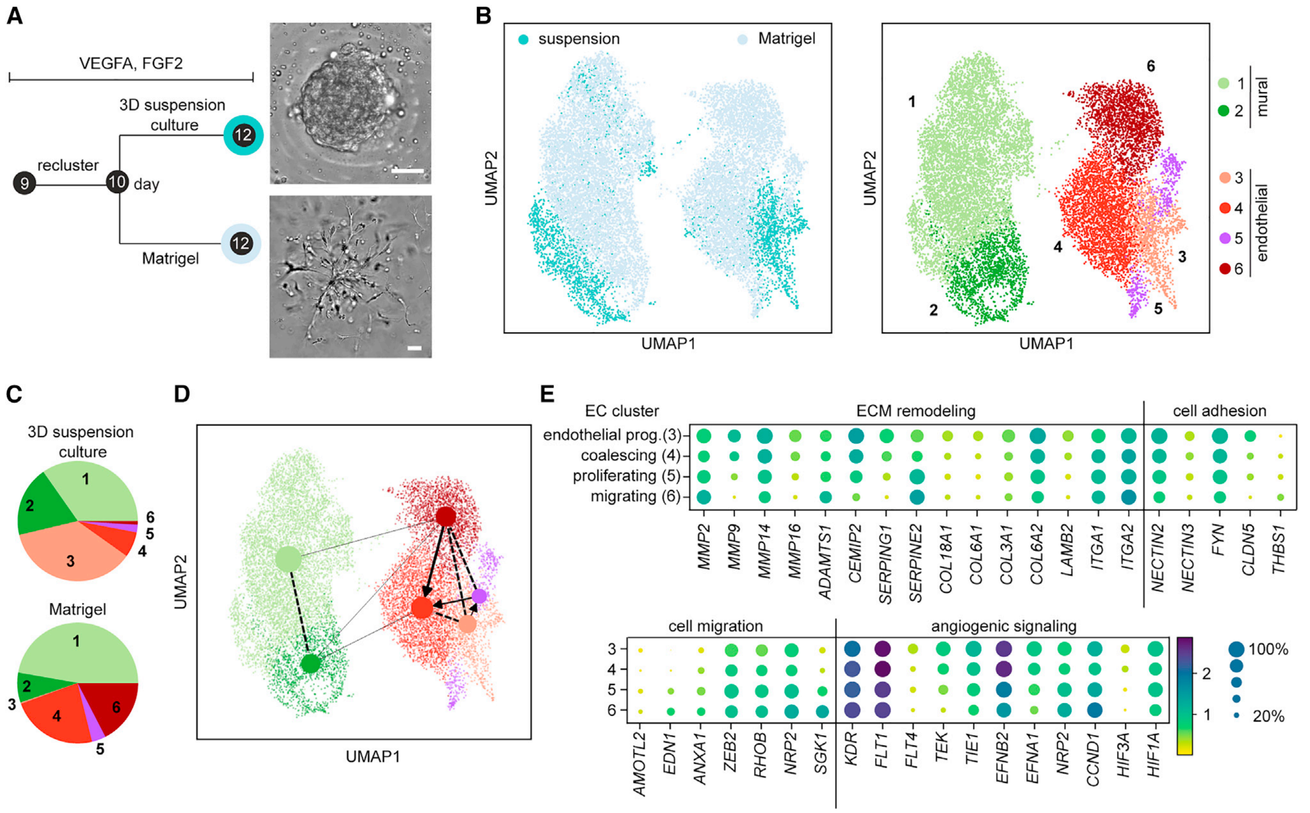

Figure 4. Single-cell analysis of hiPSC-derived ECs undergoing neovascularization in Matrigel
(A) Experimental overview of the culturing conditions for microvessel formation induction and representative bright-field images of cell culture morphologies. Scale bar: 50 μm.
(B) UMAP plots containing single-cell transcriptomes of cells from 3D suspension and Matrigel cultures. The color code denotes conditions (left) and Leiden cell clusters (right; 1 and 2, mural cells; 3, endothelial progenitor cell; 4, EC coalescing; 5, EC proliferating; 6, EC migrating).
(C) Cell type composition of the two culturing conditions is represented as a pie chart. The percentage of each cell population is given in Table S4.
(D) Velocity analysis of the single-cell transcriptomic data from (A).
(E) Expression levels of representative DEGs sorted by function for the three EC transcriptional states. The color intensity and dot size denote the normalized cluster mean expression level and the fraction of cell expression for the corresponding gene, respectively.

culture (Figure 4B). In addition, mural cells formed two transcriptomic subclusters, where one cluster overlapped with the transcriptomic state of the mural cells in the 3D suspension culture. The expression levels of general cell-type markers for the assignment of mural and EC clusters are shown in Figure S3B. The fraction of mural cells and ECs was comparable to that found in the 3D suspension culture (Figure 4C). A corresponding PAGA analysis on the RNA velocity showed the connectivity between the transcriptional states of ECs and mural cells in the Matrigel and suspension culture cell types (Figure 4D).

For the formation of microvessels, Matrigel-embedded endothelial progenitor cells must migrate into the hydrogel and coalesce. We plotted exemplary DEGs related to ECM remodeling, cell migration and motility, cell interaction, and VEGF signaling (Figure 4E) to determine the genes that induced the processes and assigned transcriptional states to the main EC cluster. Genes associated with cell migration gradually increased from the endothelial progenitor state within the 3D suspension culture to the EC clusters 4 and 6 in Matrigel (e.g., *ANXA1* or *ZEB2*). In contrast, gene expression for cell adhesion (e.g., *NECTIN2/3* or *CLDN5*) and ECM (e.g., *COL3*, *6*, and *18*) gradually decreased from the endothelial progenitor cells to ECs of clusters 4 and 6. This argued that ECs of clusters 4 and 6 represent the coalescing and migrating cell states, respectively. To investigate this further, we stained DEGs between the two clusters within cryosections of hydrogel culture from day 12 of differentiation. For ECs of cluster 4, *DLL4* and *CLDN5* were among the DEGs. Both proteins

showed stronger expression in cells with interacting partners than in isolated ECs (Figures S3D–S3G). This further indicated that ECs of cluster 4 are coalescence ECs (cECs). Immunofluorescence (IF) staining of DEGs of cluster 6 was not successful. For example, the strongest DEG of cluster 4 was the serum and glucocorticoid kinase 1 (*SGK1*), for which antibodies in the IF staining were not specific. However, it is known that ablation of *SGK1* in mice leads to a strong reduction of EC migration. Corroboratively, a gene ontology (GO)-term analysis of the DEGs of cluster 6 also indicated the enrichment of genes associated with cell migration (Figure S4A). Furthermore, a TFEA of the DEGs of migrating ECs (mECs) showed enrichment of *ELK3*, *KLF6*, and the epithelial-mesenchymal transition driver *SNAI2* (Figure S4B). Based on these findings, we assigned cluster 6 to mECs. The additional EC cluster 5 can be assigned to proliferating ECs.

Within the DEGs of the ECs were key proteins of the VEGF, Notch, and mTOR signaling pathways. The most obvious was the upregulation of *KDR* and *NRP2* in mECs, both of which act on endothelial motility, sprouting, and survival (Gerhardt et al., 2003). Within the Notch signaling pathway, mECs downregulated *NOTCH1*, *NOTCH4*, *DLL4*, and *JAG1* (Figure S4C). This could be expected, since Notch inhibition in cellular model systems has been shown to induce sprouting, branching, and filopodia induction (Lobov et al., 2007). Most interestingly, the mTOR pathway proteins, particularly those of the mTOR complex 2 (*RICTOR*), were strongly downregulated in mECs. One downstream target of mTORC2 is *SGK1*, which is the top upregulated gene in mECs, indicating strong metabolic regulation in this motile cell state (Zarrinpashneh et al., 2013). Furthermore, *DEPTOR*, an adaptor protein for mTOR complexes 1 and 2, was downregulated and concomitantly a DEG for cECs. IF images showed that in mECs, DEPTOR exhibits a nuclear location, and in cECs, it could be detected in the nucleus and in the cytoplasm, demonstrating regulatory involvement of the mTOR pathway during EC migration (Figure S4C). Notably, the expression levels of integrins changed only slightly between the EC transcriptional states.

EC maturation in Matrigel

Upon prolongation of the culturing time, the vessels grew and branched in the Matrigel microenvironment. To determine the genes activated during vessel maturation, we acquired single-cell transcriptomes of day 18 Matrigel cultures. In addition, we investigated the effects of ascorbic acid (AA) on EC maturation. AA increases the synthesis of the basal laminal protein collagen IV and reduces vessel permeability (Utoguchi et al., 1995). Bright-field imaging showed that vessel length and branching network were comparable in the presence and absence of AA (Figure 5A). To extract DEGs responsible for EC maturation, single-cell transcriptomes of all day 12 and 18 Matrigel cultures were clustered together (Figure 5B). ECs formed on day 18 of differentiation in the presence and absence of AA, where a low fraction of migrating and coalescing cells was still observed on day 18 (Figure 5C). Upon addition of AA to the cell culture medium, the fraction of mural cells increased compared with cell culture without AA (Figure 5C). A cell-cycle analysis of the scRNA dataset showed that addition of AA led to an increase in the number of proliferating cells in all cell clusters, but proportionally more in the mural cell cluster (Figures S5A and S5B). The transcriptomes of the mural cells from day 18 converged partially with the transcriptomes of day 12 and formed in total three clusters (clusters 1–3). Next to the EC and mural cell clusters, the scRNA-seq data revealed a cell cluster in which cells expressed mural and endothelial markers, including *PDGFRB* and *PECAM1* (Figure S5C). In addition to the higher expression of endothelial marker genes, a Pearson correlation analysis of the variable genes revealed higher proximity to mural cell clusters than the endothelial ones (Figure S5D). The PAGA connectivity based on the RNA velocity indicated that this population developed from the mural cells toward ECs (Figure 5D), wherefore we assigned these cells to mesenchymal-to-endothelial transitional cells (MEndoT).

Evaluation of the DEGs between ECs revealed that on day 18, ECs increased the expression levels of cell-cell contact genes such as *ICAM*s and *CLDN5* (Figure 5E). Furthermore, the expression of key tubulogenesis genes, such as *RASIP1*, *RHOB*, *ELMO1*, and *ARHGAP29* (Xu and Cleaver, 2011), was upregulated. In addition, the expression level of the *DOCK9* gene, which is a *RAC1* activator responsible for vascular lateral branching (Abraham et al., 2015), increased. Based on the expression pattern, we assigned day 18 ECs to tubulogenic ECs (tECs). To confirm this, we prepared IF staining of ICAM2 and CLDN5 within cryosections of day 12 and 18 hydrogel cultures (Figures S3F and S3G). Both DEG markers increased clearly in vessel structures on day 18. In line with the PAGA analysis, a Pearson correlation of the EC transcriptional states from days 12 and 18 showed that the tubulogenic state was closer to the coalescing than to the migrating state (Figure S5D). In addition to the changes in the levels of genes controlling the structural change, Notch signaling was upregulated again compared with the migrating and coalescent states in the forms of *NOTCH1*, *NOTCH4*, and *DLL4*. By increasing the resolution of the Leiden clustering, it is possible to separate ECs cultured in the presence and absence of AA; however, DEG change was minimal. TF analysis of the DEGs from tECs did not reveal differences in cECs. For comparison, we plotted literature-reported EC cell-type markers for the mEC, cEC, and tEC transcriptional states (Figures S6A and S6B).

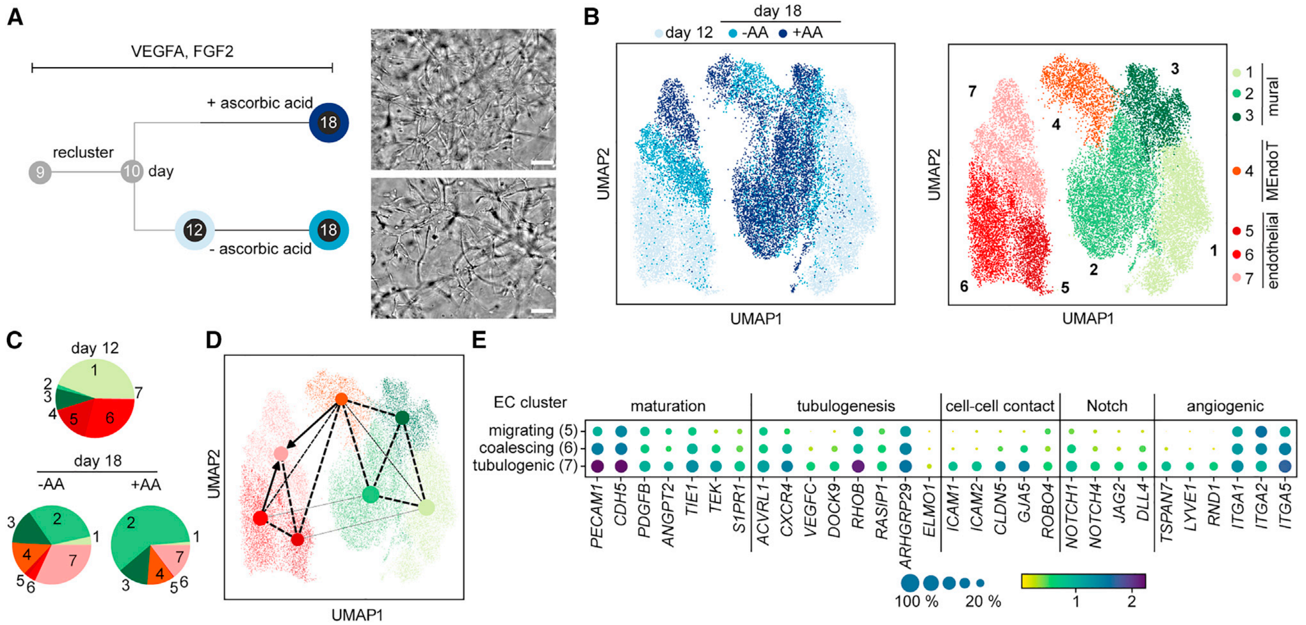

Figure 5. Maturation of endothelial cells in Matrigel in the presence and absence of ascorbic acid

(A) Experimental timeline and applied conditions with corresponding bright-field images of stem cell-derived EC and mural cell cultures in Matrigel on day 18. Scale bar: 50 μm.

(B) Left: UMAP plot of scRNA-seq data from day 12 and two samples from day 18. Right: UMAP plot colored for the annotated clusters 1, mural cell; 2 and 3, pericytes (P1 and P2); 4, MEndoT; 5, migrating ECs; 6, coalescing ECs; 7, tubulogenic ECs. The dataset contains 13,159, 6,373, and 8,684 cells for days 12 and 18 without and with ascorbic acid in the medium, respectively.

(C) Cell-type composition in the samples. Cell-type compositions are also given in Table S4.

(D) Overlay of the PAGA and UMAP plots with connectivity lines, indicating the developmental directionality based on the velocity of the single-cell transcriptomes. The dashed lines represent the connectivity between the different clusters. Here, the edge weights indicate the confidence of the connection. The arrows denote a directionality that is based on the mRNA-velocity (spliced vs unspliced).

(E) Expression levels of representative DEGs sorted by function for the three EC transcriptional states. The color intensity and dot size denote the normalized cluster mean expression level and the fraction of cell expression for the corresponding gene, respectively.

To investigate possible toning during the maturation process of tECs, we calculated a gene expression enrichment score of the top 300 DEGs of early arterial and venous cells from human embryos at Carnegie stages 10 and 11 (Zeng et al., 2019) (Figures S6C–S6E) within our day 12 and 18 differentiation dataset. Strikingly, within the top 300 DEGs of the ECs, genes of the early arterial cells increased within tECs but not early venous genes.

Mural and endothelial cell-cell interaction

The single-cell transcriptomes of the microvascular culture on day 18 revealed that mural cells of clusters 2 and 3 (Figure 5B) expressed pericyte markers *NG2*, *RGS5*, or *NT5E*. Among the DEGs were further genes previously found to be enriched in pericytes, such as *POSTN* and *PDLIM3*. IF stainings of cryosections from day 18 hydrogel cultures showed that, indeed, PDGFRB-positive cells aligned on the PECAM1 cells (Figure S7). Therefore, we assigned mural clusters 2 and 3 as pericytes, P1 and P2, respectively. While cells of the P1 cluster expressed the TF *FOXF1*, cells of the P2 expressed *GATA4* (Figure 6A), whereas both were found in the primary pericytes (Muhl et al., 2020). Localization of the subcellular pericytes within the Matrigel culture failed due to either the low amount of specific antibodies for the DEGs or the cross-expression of the markers. Mural cells of cluster 1 consisted mainly of cells from day 12 and clustered with mural cells of the 3D suspension culture. The higher expression level for the mesenchymal TF *HAND1/2* and *ACTA2* argued that mural cells of cluster 1 were in a premature stage. The velocity analysis of the mural cell transcriptomes, however, did not show any direction or evolving latent time, highlighting the plasticity of the cells. To support the mural cell assignment, we plotted literature-reported mesenchymal and fibroblast cell-type markers for the mural transcriptional states (Figure S6F).

In the last step, we asked whether it is possible to infer cell-cell communication signals leading to vessel maturation from the scRNA datasets of highly plastic cell types, stability, reduced proliferation, and migration. To trace cell-cell signaling between pericytes and tECs, we performed

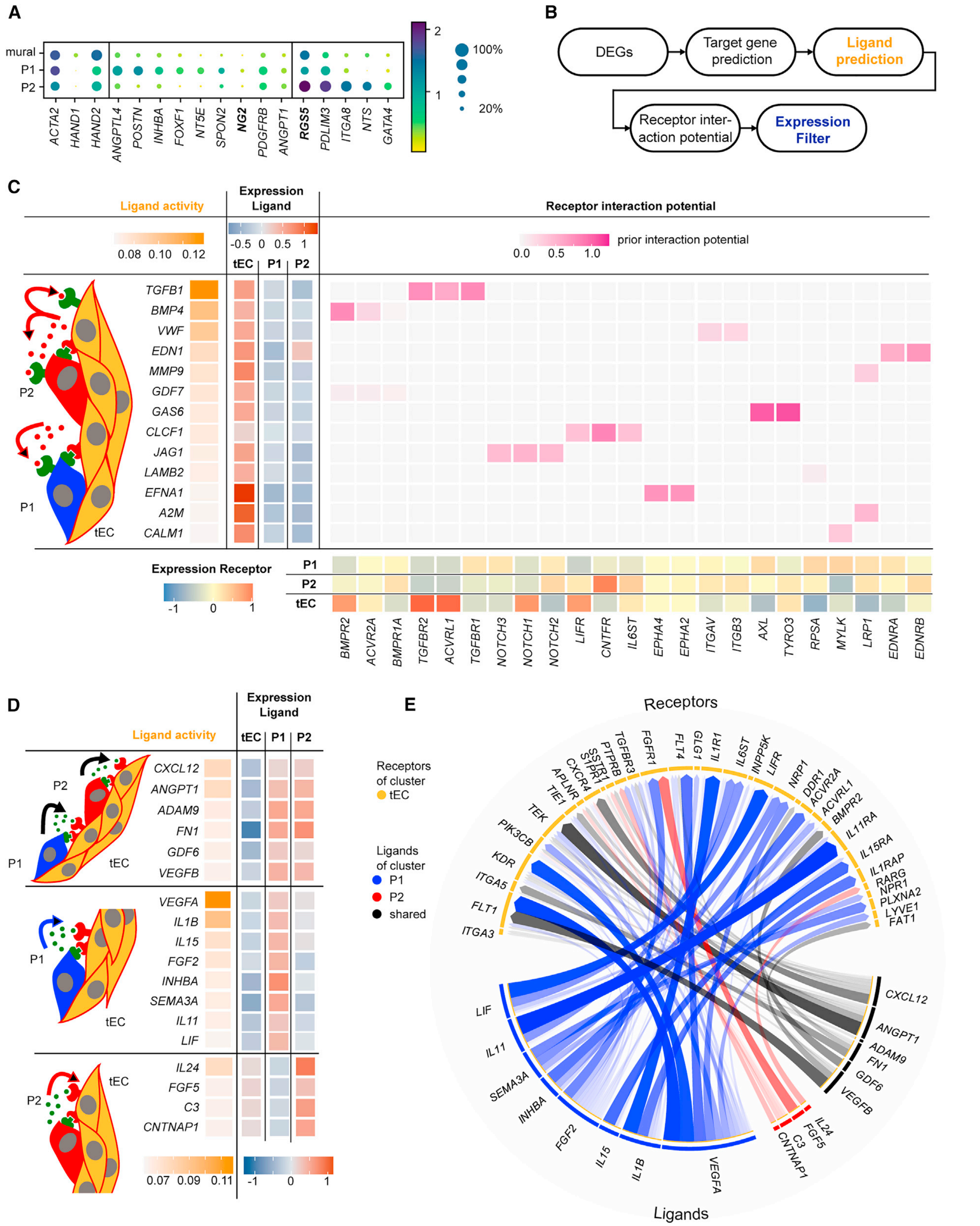

(legend on next page)

a ligand-receptor analysis on the single-cell transcriptomic data. Algorithms evaluating only ligand-receptor expression profiles could not recapitulate literature-confirmed signals between ECs and pericytes, for example, *TGFB1* or *VWF*, in our dataset. Therefore, we performed a NicheNet analysis (Browaeys et al., 2020), which infers ligand-target links between interacting cells by combining DEG data with prior knowledge of signaling and gene-regulatory networks (Figure 6B). NicheNet was applied bidirectionally by investigating ECs as ligand sender and receiver cells. The NicheNet results, that is, ligand activities and receptor and target gene interaction potential, were further filtered for gene expression levels to increase the probability of finding relevant interaction links (Figure 6C). For ECs as senders and pericytes as receivers, the algorithm predicted strong ligand activity for *TGFB1*, *BMP4*, and *VWF*. All three have been reported previously to be central for communication between both cell types (Sweeney and Foldes, 2018). *TGFB1* acts in paracrine and autocrine signaling, while tECs express the *TGFB1* receptor *ALK1* (*ACVRL1*) and pericytes express *ALK5* (*TGFBR1*), which is consistent with current reports using *in vitro* culture systems (Dave et al., 2018). The inferred ligand activity for *PDGFB* was low and thus filtered out, although *PDGFB* was highly expressed. This is explained by the fact that we used DEGs of the entire differentiation trajectory to rank ligands, and since *PDGFB* is always expressed strongly, the corresponding target genes within the DEG cannot be expected. In turn, the top predicted ligand activities represented signals that occurred during vessel maturation rather than during vessel formation and the pericyte recruitment phase. Individual NicheNet analyses between ECs and P1 or P2 cells did not differ from the combined analysis. In contrast, the NicheNet analysis for P1 or P2 as signal senders and tECs as the receivers showed that P1 and P2 pericytes exhibited shared, as well as individual, ligands (Figure 6D). The shared ligands included *CXCL12*, *ANGPT1*, fibronectin, and *GDF6*, which antagonize VEGF signaling to promote junctional stability and vascular integrity (Krispin et al., 2018; Salcedo and Oppenheim, 2010). The strongest individual predicted ligand activity of P1 cells was *VEGFA*, followed by a set of cytokines (*IL1b* and *IL15*) and *FGF2*. For P2 cells, the ligands with the strongest individual activity were *IL24* and *FGF5*. The corresponding tEC receptors are shown in Figure 6E. All inferred cytokines have proven pro- or anti-angiogenic function in cancer angiogenesis (Table S4); however, their function during neovascularization and vessel maturation is unknown.

To demonstrate that the identified signaling proteins have an angiogenic function, we performed an *in vitro* cell migration assay to test the inferred ligands for their capability to attract stem cell-derived ECs (SC-ECs). For this, we selected 10 ligands from the cell-cell communication analysis (Figure 6D), i.e., two EC, two P1/P2, four P1, and two P2 sender ligands. The migration assay was established on a commercially available microfluidic chip platform, which comprised three converging microfluidic channels (Figure 7). Within the middle microchannel, a COL1 hydrogel was formed to fluidically separate the two outer microchannels. We tested if SC-ECs enter the COL1 hydrogel from one microchannel side when adding the ligand on the opposing microchannel. The attractant capability of the ligands was evaluated by the increase in cell confluency in the hydrogel area compared with a negative control experiment with cell medium only. Five of the 10 tested ligands significantly increased the migration of the SC-ECs. TGF-β1 was one of the ligands that showed a high activity for attracting SC-ECs, but was expressed only by ECs and thus acted in the 3D cell culture system in autocrine mode. While the ligands IL-1b, InhibinA, and FGF2 are paracrine-active ligands expressed from pericytes of the cell cluster P1, IL-24 was a paracrine-active ligand expressed from pericytes of cell cluster P2. In summary, the analysis revealed signaling molecules acting between pericytes and ECs during vasculogenesis. Further, the resolved expression heterogeneity of ligands between pericytes argues for functional differences between P1 and P2 during vessel maturation.

DISCUSSION

Here, we investigated the differentiation of hiPSCs into ECs in a 3D suspension culture and, subsequently, the process of neovascularization with the stem cell-derived ECs and

Figure 6. Inferred signaling between endothelial cells and pericytes during vessel maturation from single-cell transcriptomics
(A) Gene expression analysis of the mural cells cultured in Matrigel up to day 18 of differentiation. The P1 and P2 clusters show the expression of pericyte markers (bold). The color intensity and dot size denote the normalized cluster mean expression level and the fraction of cell expression for the corresponding gene, respectively.
(B) NicheNet analysis workflow with the expression filter to infer ligand-receptor interactions.
(C) Ligand-receptor pairs inferred with NicheNet, where single-cell transcriptomes of tECs are used as senders and the combined P1 and P2 pericyte subpopulations as receivers for the analysis.
(D and E) Ligand-receptor pairs inferred with NicheNet, where single-cell transcriptomes of the P1 and P2 pericyte subpopulations are used as senders and tECs as receiver cells for the analysis. Overlapping (top rows) and unique pericyte ligands (middle and bottom rows) are listed in table format. Ligand-receptor pairs are presented in the circular chord diagram.

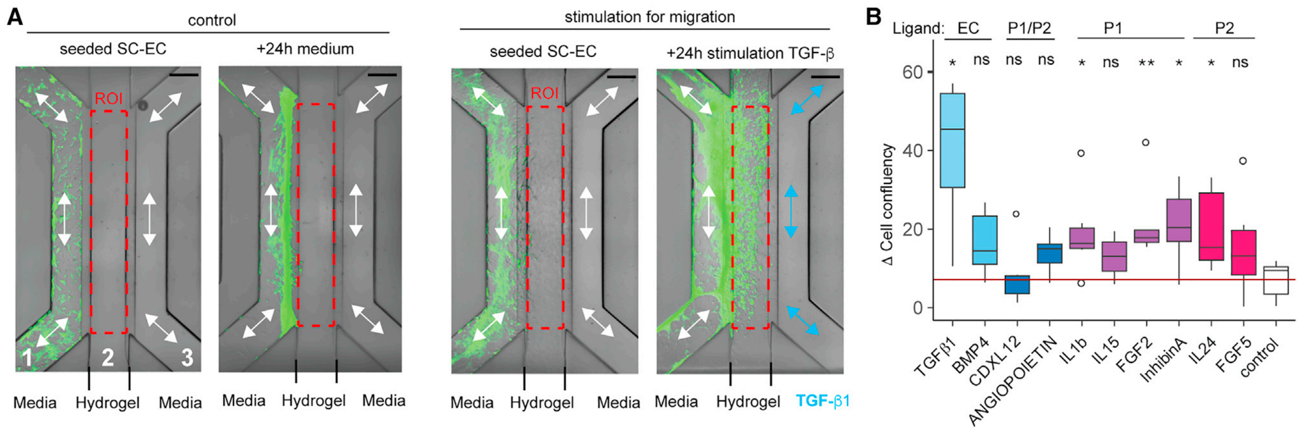

Figure 7. Testing of inferred pericyte ligands to attract stem cell-derived endothelial cells (SC-ECs)

(A) Microfluidic chip for testing the ligand activity. While GFP-expressing SC-ECs were seeded within the left microchannel of the chip, within the right microchannel the ligand was applied. Migration of SC-ECs into the Col-1 hydrogel was evaluated by measuring the cell confluency in the region of interest after 24 h of stimulation (ROI, red dashed box). Scale bar denotes 100 μm.

(B) Boxplot shows the increase in cell confluency in the hydrogel area (ROI) in the presence of the ligand within the opposing microchannel. A Mann-Whitney U test with exact p value estimation was performed for significance testing ($n \geq 4$ for each ligand). Significance levels are $^*p < 0.05$, $^{**}p < 0.01$.

evolving mural cells at the single-cell level. We found that the differentiation trajectory in the early stage of ECs and mural cells in a 3D suspension culture resembled the development of that in a monolayer format. In both cell culture approaches, one common mesodermal progenitor cell type is formed at day 3 of differentiation before the EC and mural cells evolve. In contrast to the 2D cell culture approach, ECs become quiescent in the 3D suspension culture without entering the neovascularization process. Cell communication between endothelial progenitor cells and mural cells exists within the 3D suspension culture, which is exemplified by the finding that, upon separation from the ECs, the plastic mural cells lost mesenchymal cell-type marker expression of *PDGFRB*. Mesodermal cells express *TGFB1* and *PDGFB*, where the combination of these two factors is used to induce vascular smooth muscle cell development *in vitro* in the same mesodermal precursor cells (Patsch et al., 2015). Lineage specification from day 6 of differentiation into an arterial tone could be observed when taking the stronger expression level of *EFNB2* into account (Zhang et al., 2017) (Figure 1C). Within the hydrogel culture, transcriptomes of the SC-ECs showed high gene enrichment scores for early arterials in embryos from Carnegie stages 10 and 11, confirming the toning (Figures S6E and S6F). Tissue-specific EC gene enrichment could not be detected. For scaling EC production with a 3D suspension culture, the transcriptional cell-type analysis argues that 6 days of differentiation is optimal due to the largest EC-to-mural cell ratio.

Transfer of the 3D suspension culture into Matrigel led to sprouting of the ECs. Single-cell transcriptomics untangles migrating, coalescent, and tubulogenic EC states during neovascularization. In comparison with the *in vivo* reported data, after laser ablation in the choroid layer of mouse eyes, the cellular complexity is far lower. In addition to the expected VEGF and Notch signaling pathways, the mTOR pathway is regulated during neovascularization. mTOR kinase controls various processes; when complexed in the mTORC1, it controls predominantly the cell metabolism. In the metabolism, for example, a switch between OXPHOS and glycolysis for mECs could not be observed as described for tip cells *in vivo* (Rohlenova et al., 2020). However, in mECs, mTORC2 adaptor proteins were downregulated, and the downstream effector kinase *SGK1*, which controls cell survival during angiogenesis (Catela et al., 2010) and EC shape (Tsuji-Tamura and Ogawa, 2016), was strongly upregulated; its ablation led to reduced neovascularization and impaired cell migration (Zarrinpashneh et al., 2013). The function and regulation of *SGK1* through mTORC2 are unknown, but have become the focus of further investigations.

Vessel structure formation within the hydrogel culture was accompanied by a change in ECM-integrin expression level. While *ITGA2* was upregulated in mECs, a gradual increase in *ITGA1* and *ITGA5* was detected toward the cEC and tEC stages (Figure 5E). Blocking collagen-binding ITGA1 and ITGA2 with antibodies has been shown to reduce angiogenesis (Senger et al., 2002). The EC and mural cell stage-assigned integrin profiles provide new dynamic insights. The addition of AA to the cell culture medium increased the expression level and deposition of the central basal lamina protein COL4 (Figure S6E). Therefore, the

AA-induced maturation of ECs increased the proliferation rate of mural cells. This may be unwanted in long-term cultures during tissue engineering with no proliferative cells. ECs on day 18 of differentiation did not show a specification marker for arterial or any other vascular cell type. Notably, the scRNA-seq data from day 18 of the Matrigel cell cultures indicated a mesenchymal-to-endothelial transition. Such a mural cell plasticity has been observed before in heart tissue after injury (Ubil et al., 2014), but not in *in vitro* conditions.

The most compelling results of the single-cell transcriptomic analysis during the vessel maturation phase in a reduced *in vitro* microenvironment were the resolved cell-cell communication signals between the two transcriptional pericyte subpopulations and tECs. Pericytes are recruited by PDGFB signaling to capillary walls to stabilize integrity and tube assembly (Lindahl et al., 1997). Next to the known EC-pericyte signaling factors, including further FGF2 and VEGFA, the complex cytokine profiles of the pericytes was resolved by the directionality of the NicheNet ligand-receptor analysis. Here, ligands from the two transcriptional pericyte states, P1 and P2, were inferred, and their activity in attracting EC migration, i.e., IL-1b or IL-24, respectively, was confirmed. In addition, we showed experimentally that inferred *TGFB1* autocrine signals, where sender and ligands are expressed only by tECs, are active in attracting SC-ECs in an *in vitro* assay. The previous signaling axis CXCR4/CXCL12 for pericyte vessel maturation (Salcedo et al., 1999) was also here detected as CSCL12 sent from pericytes to ECs. However, ligand testing of CXCL12 for attraction of EC migration was negative. The present single-cell transcriptomic data and analysis of the vascular structure formation process can be used as a benchmark set for future *in vitro* vascularization approaches, differentiation attempts to alter the specification of ECs, or investigation into disease-specific gene functions.

EXPERIMENTAL PROCEDURES

Resource availability

Corresponding author

Further information should be directed to Matthias Meier (matthias.meier@helmholtz-munich.de).

Materials availability

This study did not generate new unique reagents nor use new biological samples.

Data and code availability

The code for scRNA-seq analysis has been deposited on https://github.com/MeierLabMiBioEng/scRNA_3D_EC_differentiation GitHub and is publicly available. DOIs are listed in the key resources table. Raw data are publicly available on the Gene Expression Omnibus repository under accession code GSE196799. Any additional information required to reanalyze the data reported in this paper is available from the corresponding author upon request.

Experimental model details

When not specified otherwise, experiments were conducted using a hiPSC line, which was kindly provided by Prof. Lickert (Helmoltz Zentrum Munich) and is registered in the human pluripotent stem cell registry (https://hpscreg.eu) under the name HMGUi002-A. The cell line was derived from a Caucasian male donor. The control iPSC line was purchased from the Coriell Institute (Coriell Institute, cat. no. AICS-0036-028). The cell line (AICS) expressed mEGFP constantly from the AAV1 locus and was derived from a parental line from an Asian donor. The general scientific use of the HMGUi002-A cell line was approved by the local ethics committee at the Technical University Munich (reference no. 400/21 S-KH).

Two-dimensional hiPSC cell culture

Human iPSCs were cultured on hESC Matrigel-precoated six-well plates according to the manufacturer's recommendations (Corning, cat. no. 354277) in mTeSR1 medium (StemCell Technologies, cat. no. 85850) at 5% CO_2, 5% O_2, and 37°C with daily medium change and passaged twice a week in a 1:6 ratio using 0.05% trypsin-EDTA (Sigma Aldrich, cat. no. T4174). The hiPSC culture was free of mycoplasma contamination as tested by the MycoSensor PCR assay kit (Agilent Technology, cat. no. 302109).

Three-dimensional suspension culture

For the transfer into a 3D hiPSC cell culture, the medium was aspirated, cells were washed with 2 mL PBS−/−, and 500 μL Accutase (Sigma Aldrich, cat. no. A6964) was added. During incubation at 37°C for 5 min, the cells detached. Addition of 2.5 mL mTeSR stopped the Accutase reaction. The wells were washed with 1 mL mTeSR before centrifugation for 5 min at 200 × *g* and resuspended in 500 μL mTeSR with 10 μM Rock inhibitor Y-27632 dihydrochloride (Abcam, cat. no. HY-10583) and 1% penicillin/streptomycin (P/S) (Thermo Fisher Scientific, cat. no. 15140122). Cells were transferred to six-well ultra-low-attachment plates (Corning, cat. no. 3471) at a cell concentration of 5×10^5 cells/mL. The plate was placed on an orbital shaker in the cell incubator and rotated at a frequency of 100 rpm.

Three-dimensional suspension culture differentiation to ECs

On day −1 of the differentiation, cells were transferred into low-attachment six-well plates at a concentration of 1.5×10^6 cells/well. The differentiation protocol was adopted by combining the protocols from Olmer et al. (2018), Patsch et al. (2015), and Wimmer et al. (2019). From day 0 to day 3 of differentiation, Neurobasal medium with B27 supplement (Life Technologies, cat. nos. 21103049 and 12587010) supplemented with BMP4 (25 ng/mL) (PeproTech, cat. no. 120-05ET-10) and CHIR 99021 (7.5 μM) (Axon Medchem, cat. no. 1385) was used without medium exchange. Medium was changed daily from day 3 to day 7 of differentiation using StemPro-34 (Life Technologies, cat. no. 10639011) with VEGFA (200 ng/mL) (PeproTech,

cat. no. 10-20-100) and forskolin (2 μM) (Abcam, cat. no. ab120058). Afterward, StemPro-34 with VEGFA (30 ng/mL) and FGF2 (30 ng/mL) (Miltenyi Biotech, cat. no. 130-093-838) was used with exchange after 2 days.

Two-dimensional endothelial and mural cell culture

Sorted endothelial and mural cells were cultured in T75 flasks (Sarstedt, cat. no. 83.3911.002). Flasks were coated with a 5 μg/mL fibronectin (Life Technologies, cat. no. 33010018) solution before use. Endothelial and mural cells were cultured in StemPro with 15% fetal bovine serum (FBS), 100 ng/mL VEGFA, and 100 ng/mL FGF2.

Hydrogel cell culture

For cultivation in 24-well plates (Corning, cat. no. 353047), 100 μL undiluted hESC-Qualified Matrigel (Corning, cat. no. 354277) was added into each well of the plate and incubated at 37°C for 1 h. Aggregates were centrifuged at 800 rpm for 5 min at 4°C, resuspended in 80 μL of Matrigel, and spread on top of the first hydrogel layer. Matrigel polymerization was done at 37°C inside the cell incubator on ice to allow a slow temperature adjustment. After 1 h, 0.5 mL medium (StemPro-34 with 100 ng/mL VEGFA, 100 ng/mL FGF2, and 15% FBS; Fisher Scientific, cat. no. 15377636) was added for cultivation. The medium was exchanged every second day. Samples with AA contained 60 μg/mL AA (Sigma Aldrich, cat. no. A4544). For imaging, cell aggregates were embedded in Matrigel on μ-Slides (IBIDI, cat. no. 80826) with 10–20 per well.

Cell analysis

Standard FACS, flow cytometry, and cryoembedding procedures are given in the supplemental experimental procedures.

Cell-type stability experiments

On day 6, aggregates were harvested and prepared for FACS (see Figure S5), and ECs (PECAM1$^+$, PDGFRB$^-$) and mural cells (PDGFRB$^+$, PECAM1$^-$) were sorted. The sorted cell types (2×10^5 each) and the unsorted mixture were seeded and cultured in six-well plates coated with fibronectin bovine (Life Technologies, cat. no. 33010018). The medium composition was equivalent to the 3D differentiation culture from day 6 on.

Fluorescence imaging

Slides were washed with PBS, permeabilized in PBS with 0.1% Triton (Sigma Aldrich, cat. no. 93443) for 30 min at room temperature, washed with 0.2% Tween 20 in PBS (PBST), and blocked with 2% BSA (Proliant Biologicals, cat. no. 68700) in PBST for 1 h. Antibodies were applied in the concentrations recommended by the manufacturer's specifications in the blocking solution. After primary and secondary antibody staining, five washing steps of 5 min with PBST were performed. Before confocal imaging (Zeiss Axio Observer LSM 880), Vectashield mounting medium (Biozol Diagnostica, cat. no. VEC-H-1000) was added to the sample, and it was covered and sealed by a coverslip.

Image analysis

IF and bright-field images were corrected for brightness and contrast with ImageJ. Z projection of fluorescence images was performed using maximal intensity. ImageJ version 1.52p was used (Schindelin et al., 2012).

Cell migration assay

A detailed description of the microfluidic cell migration assay is given in the supplemental experimental procedures.

SUPPLEMENTAL INFORMATION

Supplemental information can be found online at https://doi.org/10.1016/j.stemcr.2023.08.008.

ACKNOWLEDGMENTS

This work is supported by the Helmholtz Pioneer Campus, ERC (Consolidator Grant 772646) and BMBF – SprintD (grant eIslet 031L0251). We thank Thomas Walzthöni for the bioinformatics support provided at the Bioinformatics Core Facility, Institute of Computational Biology, Helmholtz Zentrum München.

AUTHOR CONTRIBUTIONS

S.R., M.A., and M. Meier designed the study. S.R., M.A., C.R., M. Marder, and M. Meier executed the biological experiments. S.R. and M.A. did the imaging and image analysis. J.B. and A.F. did the cryosectioning and imaging. S.R. and S.W. performed the scRNA-seq analysis. S.U., F.T., and M. Meier supervised the study. The manuscript was written by S.R. and M. Meier. All authors corrected and approved the paper.

DECLARATION OF INTERESTS

The authors declare no competing interests.

Received: October 21, 2022
Revised: August 12, 2023
Accepted: August 14, 2023
Published: September 14, 2023

REFERENCES

Abraham, S., Scarcia, M., Bagshaw, R.D., McMahon, K., Grant, G., Harvey, T., Yeo, M., Esteves, F.O.G., Thygesen, H.H., Jones, P.F., et al. (2015). A Rac/Cdc42 exchange factor complex promotes formation of lateral filopodia and blood vessel lumen morphogenesis. Nat. Commun. *6*, 1–14. https://doi.org/10.1038/ncomms8286.

Browaeys, R., Saelens, W., and Saeys, Y. (2020). NicheNet: modeling intercellular communication by linking ligands to target genes. Nat. Methods *17*, 159–162. https://doi.org/10.1038/s41592-019-0667-5.

Catela, C., Kratsios, P., Hede, M., Lang, F., and Rosenthal, N. (2010). Serum and glucocorticoid-inducible kinase 1 (SGK1) is necessary for vascular remodeling during angiogenesis. Dev. Dynam. *239*, 2149–2160. https://doi.org/10.1002/dvdy.22345.

Chatterjee, S. (2021). Endothelial Signaling in Vascular Dysfunction and Disease: From Bench to Bedside (Elsevier Science).

Chen, G., Gulbranson, D.R., Hou, Z., Bolin, J.M., Ruotti, V., Probasco, M.D., Smuga-Otto, K., Howden, S.E., Diol, N.R., Propson, N.E., et al. (2011). Chemically defined conditions for human iPSC derivation and culture. Nat. Methods *8*, 424–429. https://doi.org/10.1038/nmeth.1593.

Dave, J.M., Mirabella, T., Weatherbee, S.D., and Greif, D.M. (2018). Pericyte ALK5/TIMP3 Axis Contributes to Endothelial Morphogenesis in the Developing Brain. Dev. Cell *44*, 665–678.e6. https://doi.org/10.1016/j.devcel.2018.01.018.

Dejana, E., Hirschi, K.K., and Simons, M. (2017). The molecular basis of endothelial cell plasticity. Nat. Commun. *8*, 14361. https://doi.org/10.1038/ncomms14361.

Gerhardt, H., Golding, M., Fruttiger, M., Ruhrberg, C., Lundkvist, A., Abramsson, A., Jeltsch, M., Mitchell, C., Alitalo, K., Shima, D., et al. (2003). VEGF guides angiogenic sprouting utilizing endothelial tip cell filopodia. The Journal of Cell Biology *161*, 1163. https://doi.org/10.1083/jcb.200302047.

Holm, A., Heumann, T., and Augustin, H.G. (2018). Microvascular Mural Cell Organotypic Heterogeneity and Functional Plasticity. Trends Cell Biol. *28*, 302–316. https://doi.org/10.1016/j.tcb.2017.12.002.

Krispin, S., Stratman, A.N., Melick, C.H., Stan, R.V., Malinverno, M., Gleklen, J., Castranova, D., Dejana, E., and Weinstein, B.M. (2018). Growth Differentiation Factor 6 Promotes Vascular Stability by Restraining Vascular Endothelial Growth Factor Signaling. Arterioscler. Thromb. Vasc. Biol. *38*, 353–362. https://doi.org/10.1161/atvbaha.117.309571.

Lindahl, P., Johansson, B.R., Levéen, P., and Betsholtz, C. (1997). Pericyte loss and microaneurysm formation in PDGF-B-deficient mice. Science *277*, 242–245. https://doi.org/10.1126/science.277.5323.242.

Lobov, I.B., Renard, R.A., Papadopoulos, N., Gale, N.W., Thurston, G., Yancopoulos, G.D., and Wiegand, S.J. (2007). Delta-like ligand 4 (Dll4) is induced by VEGF as a negative regulator of angiogenic sprouting. Proc. Natl. Acad. Sci. USA *104*, 3219–3224. https://doi.org/10.1073/pnas.0611206104.

McCracken, I.R., Taylor, R.S., Kok, F.O., de la Cuesta, F., Dobie, R., Henderson, B.E.P., Mountford, J.C., Caudrillier, A., Henderson, N.C., Ponting, C.P., and Baker, A.H. (2020). Transcriptional dynamics of pluripotent stem cell-derived endothelial cell differentiation revealed by single-cell RNA sequencing. Eur. Heart J. *41*, 1024–1036. https://doi.org/10.1093/eurheartj/ehz351.

McInnes, L., Healy, J., and Melville, J. (2018). UMAP: Uniform Manifold Approximation and Projection for Dimension Reduction. Preprint at ArXiv. https://doi.org/10.48550/arXiv.1802.03426.

Muhl, L., Genové, G., Leptidis, S., Liu, J., He, L., Mocci, G., Sun, Y., Gustafsson, S., Buyandelger, B., Chivukula, I.V., et al. (2020). Single-cell analysis uncovers fibroblast heterogeneity and criteria for fibroblast and mural cell identification and discrimination. Nat. Commun. *11*, 3953. https://doi.org/10.1038/s41467-020-17740-1.

Nguyen, J., Lin, Y.-Y., and Gerecht, S. (2021). The next generation of endothelial differentiation: Tissue-specific. Cell Stem Cell *28*, 1188–1204. https://doi.org/10.1016/j.stem.2021.05.002.

Noden, D.M. (1989). Embryonic origins and assembly of blood vessels. Am. Rev. Respir. Dis. *140*, 1097–1103. https://doi.org/10.1164/ajrccm/140.4.1097.

Olmer, R., Engels, L., Usman, A., Menke, S., Malik, M.N.H., Pessler, F., Göhring, G., Göhring, G., Bolten, S., Abdelilah-Seyfried, S., et al. (2018). Differentiation of Human Pluripotent Stem Cells into Functional Endothelial Cells in Scalable Suspension Culture. Stem Cell Rep. *10*, 1657–1672. https://doi.org/10.1016/j.stemcr.2018.03.017.

Osterwalder, M., Speziale, D., Shoukry, M., Mohan, R., Ivanek, R., Kohler, M., Beisel, C., Wen, X., Scales, S.J., Christoffels, V.M., et al. (2014). HAND2 targets define a network of transcriptional regulators that compartmentalize the early limb bud mesenchyme. Dev. Cell *31*, 345–357. https://doi.org/10.1016/j.devcel.2014.09.018.

Paik, D.T., Tian, L., Williams, I.M., Rhee, S., Zhang, H., Liu, C., Mishra, R., Wu, S.M., Red-Horse, K., and Wu, J.C. (2020). Single-Cell RNA Sequencing Unveils Unique Transcriptomic Signatures of Organ-Specific Endothelial Cells. Circulation *142*, 1848–1862. https://doi.org/10.1161/circulationaha.119.041433.

Patsch, C., Challet-Meylan, L., Thoma, E.C., Urich, E., Heckel, T., O'Sullivan, J.F., Grainger, S.J., Kapp, F.G., Sun, L., Christensen, K., et al. (2015). Generation of vascular endothelial and smooth muscle cells from human pluripotent stem cells. Nat. Cell Biol. *17*, 994–1003. https://doi.org/10.1038/ncb3205.

Poole, T.J., Finkelstein, E.B., and Cox, C.M. (2001). The role of FGF and VEGF in angioblast induction and migration during vascular development. Dev. Dynam. *220*, 1–17. https://doi.org/10.1002/1097-0177(2000)9999:9999<;::aid-dvdy1087>3.0.co;2-2.

Ricard, N., Bailly, S., Guignabert, C., and Simons, M. (2021). The quiescent endothelium: signalling pathways regulating organ-specific endothelial normalcy. Nat. Rev. Cardiol. *18*, 565–580. https://doi.org/10.1038/s41569-021-00517-4.

Rohlenova, K., Goveia, J., García-Caballero, M., Subramanian, A., Kalucka, J., Treps, L., Falkenberg, K.D., de Rooij, L.P.M.H., Zheng, Y., Lin, L., et al. (2020). Single-Cell RNA Sequencing Maps Endothelial Metabolic Plasticity in Pathological Angiogenesis. Cell Metabol. *31*, 862–877.e14. https://doi.org/10.1016/j.cmet.2020.03.009.

Salcedo, R., and Oppenheim, J.J. (2003). Role of Chemokines in Angiogenesis: CXCL12/SDF-1 and CXCR4 Interaction, a Key Regulator of Endothelial Cell Responses. Microcirculation *10*, 359–370. https://doi.org/10.1038/sj.mn.7800200.

Salcedo, R., Wasserman, K., Young, H.A., Grimm, M.C., Howard, O.M., Anver, M.R., Kleinman, H.K., Murphy, W.J., and Oppenheim, J.J. (1999). Vascular endothelial growth factor and basic fibroblast growth factor induce expression of CXCR4 on human endothelial cells. In vivo neovascularization induced by stromal-derived factor-1$\alpha. Am. J. Pathol. *154*, 1125–1135. https://doi.org/10.1016/s0002-9440(10)65365-5.

Schindelin, J., Arganda-Carreras, I., Frise, E., Kaynig, V., Longair, M., Pietzsch, T., Preibisch, S., Rueden, C., Saalfeld, S., Schmid, B., et al. (2012). Fiji: an open-source platform for biological-image analysis. Nat. Methods *9*, 676–682. https://doi.org/10.1038/nmeth.2019.

Senger, D.R., Perruzzi, C.A., Streit, M., Koteliansky, V.E., de Fougerolles, A.R., and Detmar, M. (2002). The alpha(1)beta(1) and alpha(2)beta(1) integrins provide critical support for vascular endothelial growth factor signaling, endothelial cell migration, and tumor angiogenesis. Am. J. Pathol. *160*, 195–204. https://doi.org/10.1016/s0002-9440(10)64363-5.

Sweeney, M., and Foldes, G. (2018). It Takes Two: Endothelial-Perivascular Cell Cross-Talk in Vascular Development and Disease. Front. Cardiovasc. Med. *5*, 154. https://doi.org/10.3389/fcvm.2018.00154.

Tan, J.Y., Sriram, G., Rufaihah, A.J., Neoh, K.G., and Cao, T. (2013). Efficient derivation of lateral plate and paraxial mesoderm subtypes from human embryonic stem cells through GSKi-mediated differentiation. Stem Cell. Dev. *22*, 1893–1906. https://doi.org/10.1089/scd.2012.0590.

Traag, V.A., Waltman, L., and van Eck, N.J. (2019). From Louvain to Leiden: guaranteeing well-connected communities. Sci. Rep. *9*, 5233. https://doi.org/10.1038/s41598-019-41695-z.

Tsuji-Tamura, K., and Ogawa, M. (2016). Inhibition of the PI3K-Akt and mTORC1 signaling pathways promotes the elongation of vascular endothelial cells. J. Cell Sci. *129*, 1165–1178. https://doi.org/10.1242/jcs.178434.

Tyser, R.C.V., Mahammadov, E., Nakanoh, S., Vallier, L., Scialdone, A., and Srinivas, S. (2021). Single-cell transcriptomic characterization of a gastrulating human embryo. Nature *600*, 285–289. https://doi.org/10.1038/s41586-021-04158-y.

Ubil, E., Duan, J., Pillai, I.C.L., Rosa-Garrido, M., Wu, Y., Bargiacchi, F., Lu, Y., Stanbouly, S., Huang, J., Rojas, M., et al. (2014). Mesenchymal–endothelial transition contributes to cardiac neovascularization. Nature *514*, 585–590. https://doi.org/10.1038/nature13839.

Utoguchi, N., Ikeda, K., Saeki, K., Oka, N., Mizuguchi, H., Kubo, K., Nakagawa, S., and Mayumi, T. (1995). Ascorbic acid stimulates barrier function of cultured endothelial cell monolayer. J. Cell. Physiol. *163*, 393–399. https://doi.org/10.1002/jcp.1041630219.

Wimmer, R.A., Leopoldi, A., Aichinger, M., Wick, N., Hantusch, B., Novatchkova, M., Taubenschmid, J., Hämmerle, M., Esk, C., Bagley, J.A., et al. (2019). Human blood vessel organoids as a model of diabetic vasculopathy. Nature *565*, 505–510. https://doi.org/10.1038/s41586-018-0858-8.

Wu, S.-P., Dong, X.-R., Regan, J.N., Su, C., and Majesky, M.W. (2013). Tbx18 Regulates Development of the Epicardium and Coronary Vessels. Dev. Biol. *383*, 307–320. https://doi.org/10.1016/j.ydbio.2013.08.019.

Xu, K., and Cleaver, O. (2011). Tubulogenesis during blood vessel formation. Semin. Cell Dev. Biol. *22*, 993–1004. https://doi.org/10.1016/j.semcdb.2011.05.001.

Xu, M., He, J., Zhang, C., Xu, J., and Wang, Y. (2019). Strategies for derivation of endothelial lineages from human stem cells. Stem Cell Res. Ther. *10*, 200. https://doi.org/10.1186/s13287-019-1274-1.

Zarrinpashneh, E., Poggioli, T., Sarathchandra, P., Lexow, J., Monassier, L., Terracciano, C., Lang, F., Damilano, F., Zhou, J.Q., Rosenzweig, A., et al. (2013). Ablation of SGK1 Impairs Endothelial Cell Migration and Tube Formation Leading to Decreased Neo-Angiogenesis Following Myocardial Infarction. PLoS One *8*, e80268. https://doi.org/10.1371/journal.pone.0080268.

Zeng, Y., He, J., Bai, Z., Li, Z., Gong, Y., Liu, C., Ni, Y., Du, J., Ma, C., Bian, L., et al. (2019). Tracing the first hematopoietic stem cell generation in human embryo by single-cell RNA sequencing. Cell Res. *29*, 881–894. https://doi.org/10.1038/s41422-019-0228-6.

Zhang, J., Chu, L.F., Hou, Z., Schwartz, M.P., Hacker, T., Vickerman, V., Swanson, S., Leng, N., Nguyen, B.K., Elwell, A., et al. (2017). Functional characterization of human pluripotent stem cell-derived arterial endothelial cells. Proc. Natl. Acad. Sci. USA *114*, E6072–E6078. https://doi.org/10.1073/pnas.1702295114.

Stem Cell Reports, Volume *18*

Supplemental Information

Single-cell characterization of neovascularization using hiPSC-derived endothelial cells in a 3D microenvironment

Simon Rosowski, Caroline Remmert, Maren Marder, Misao Akishiba, Judith Bushe, Annette Feuchtinger, Alina Platen, Siegfried Ussar, Fabian Theis, Sandra Wiedenmann, and Matthias Meier

Supplemental Figures and legends

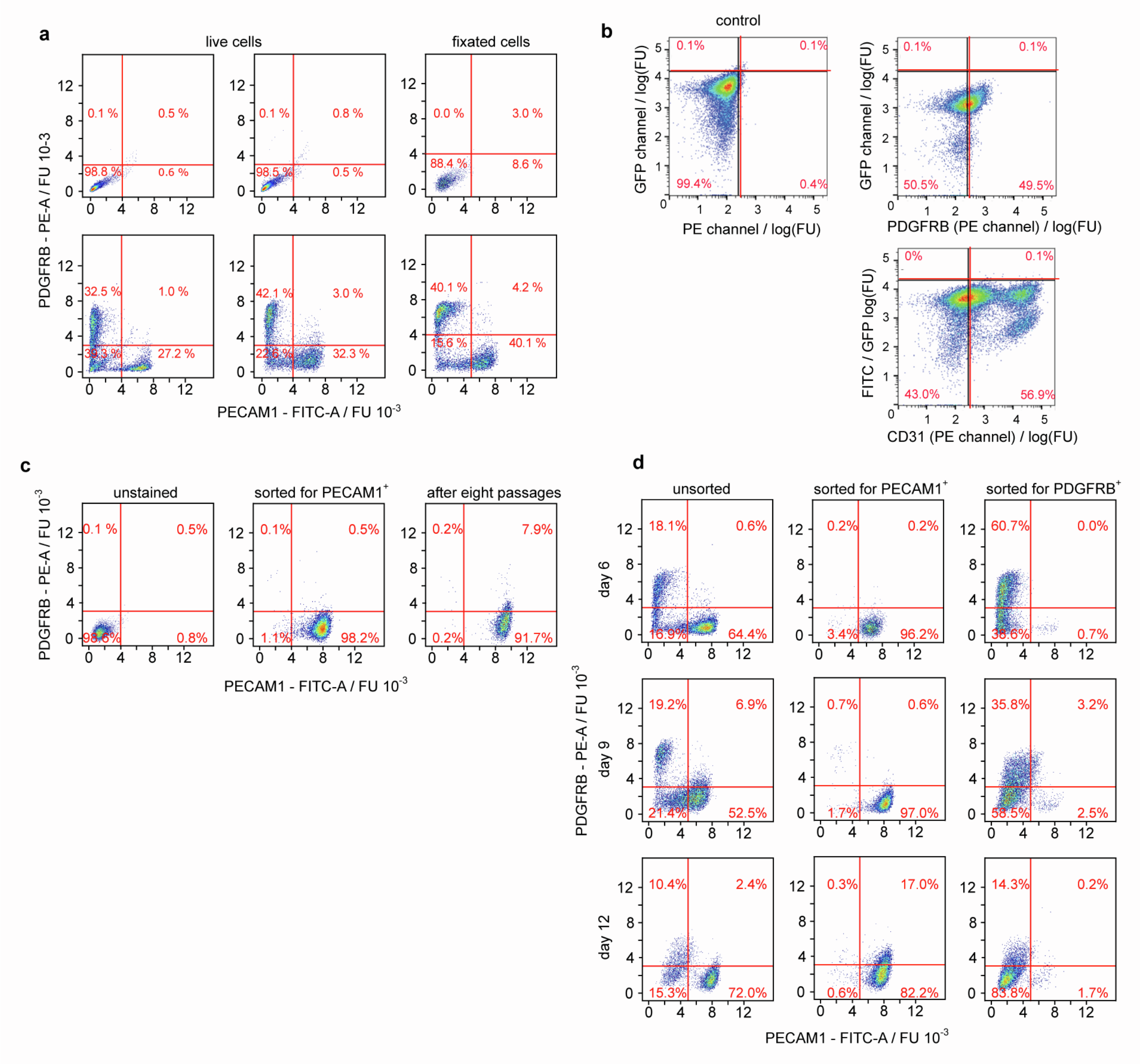

Figure S1. Reproducibility and Cell type stability and long-term culturing of stem cell-derived endothelial cells. **a**, Flow cytometric analysis of three independent EC differentiation experiments. Cells (HMGUi002-A) were stained with PECAM1-FITC and PDGFRB-PE antibodies on day nine of differentiation. Upper left, unstained cell sample control; upper center and right, isotype control of the first EC differentiation experiment; lower row, three independent EC differentiation experiments. **b**, Repeat the EC differentiation with a second fluorescently labeled hiPSC line (*Coriell Institute, Cat# AICS-0036-028*). Fluorescently labeled cells (AICS) were stained with PECAM1-PE or PDGFRB-PE antibodies on day ten of differentiation. Left: FACS plot shows the unstained cell sample control with the intrinsic fluorescence of the GFP reporter signal. Right: FACS plots of the stained sample. **c**, Flow cytometry analysis of sorted PECAM1 positive cells from 3D suspension cultures at day nine of differentiation. Cells are plated on fibronectin coated well plates for long-term culturing in 2D cell culture format within EC maturation media. Flow cytometry analysis of the cells culture in 2D after eight passages showed a comparable fraction of PECAM1 positive cells. **d,** Cell type stability of ECs and mural cells in a 2D culture format under EC maturation media condition. Upon plating 3D suspension cultures on day six of differentiation onto a 2D fibronectin coated 2D culture surface the fraction of PDGFRB positive cells gradually decreased over the time of six days (left panel from top to

bottom). PECAM sorted EC from day six 3D cell cultures maintained their PECAM marker expression over six days within the 2D culture format (middle panel from top to bottom). This is following **c** that PDGFRB sorted mural cells from day six 3D cell cultures lost the expression of PDGFRB over the monitored time frame, which argued that the cell lost their identity. The figure corresponds to the main **Figure 1**.

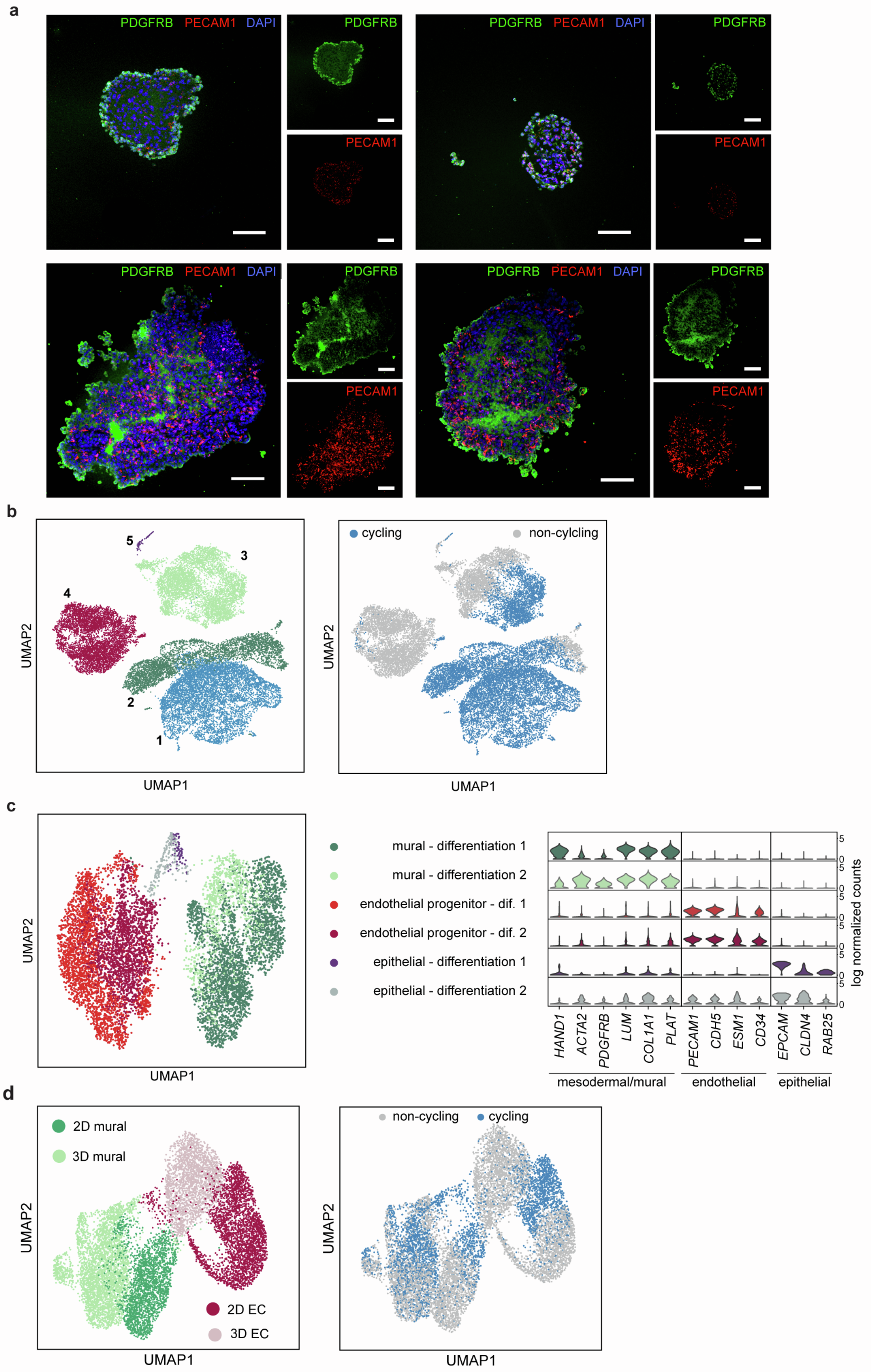
a
PDGFRB PECAM1 DAPI
PDGFRB
PECAM1
b
5
3
4
2
1
UMAP2
UMAP1
cycling
non-cylcling
c
mural - differentiation 1
mural - differentiation 2
endothelial progenitor - dif. 1
endothelial progenitor - dif. 2
epithelial - differentiation 1
epithelial - differentiation 2
HAND1
ACTA2
PDGFRB
LUM
COL1A1
PLAT
PECAM1
CDH5
ESM1
CD34
EPCAM
CLDN4
RAB25
mesodermal/mural
endothelial
epithelial
log normalized counts
d
2D mural
3D mural
2D EC
3D EC
non-cycling
cycling

Figure S2. Cell cycle analysis and reproducibility of the endothelial differentiation in a 3D suspension culture. a, **Left**: UMAP plot of the scRNA-seq dataset from experiment 1 (experiment from the main **Figure 1**), where cells in the G2, S, and M phases were colored in blue, and cells in the G1 phase in grey. **Right:**, UMAP plot corresponding to the left plot with color code for Leiden clustering and cell type annotation. **b**, **Left**: UMAP plot of single-cell transcriptomes from two independent differentiation experiments. The color code denotes the cell types found in the two differentiations. 6618 cells were analyzed in the first and 5035 in the second sequencing experiment. **Right:** Violin plot of common cell type marker genes for annotation of the clusters. The density distribution indicates the normalized cluster mean expression. **c**, **Left**: UMAP plot shows the combined sc-transcriptomes acquired for EC differentiated in 3D suspension and 2D dish culture up to day nine (our study) and eight (McCracken et al.), respectively. **Right**: UMAP plot shows the cells with gene expression relating to S, G2, or M-phase (blue). Cells expressing genes indicative of the G1 phase are denoted with a grey color. The figure corresponds to the main **Figure 3**.

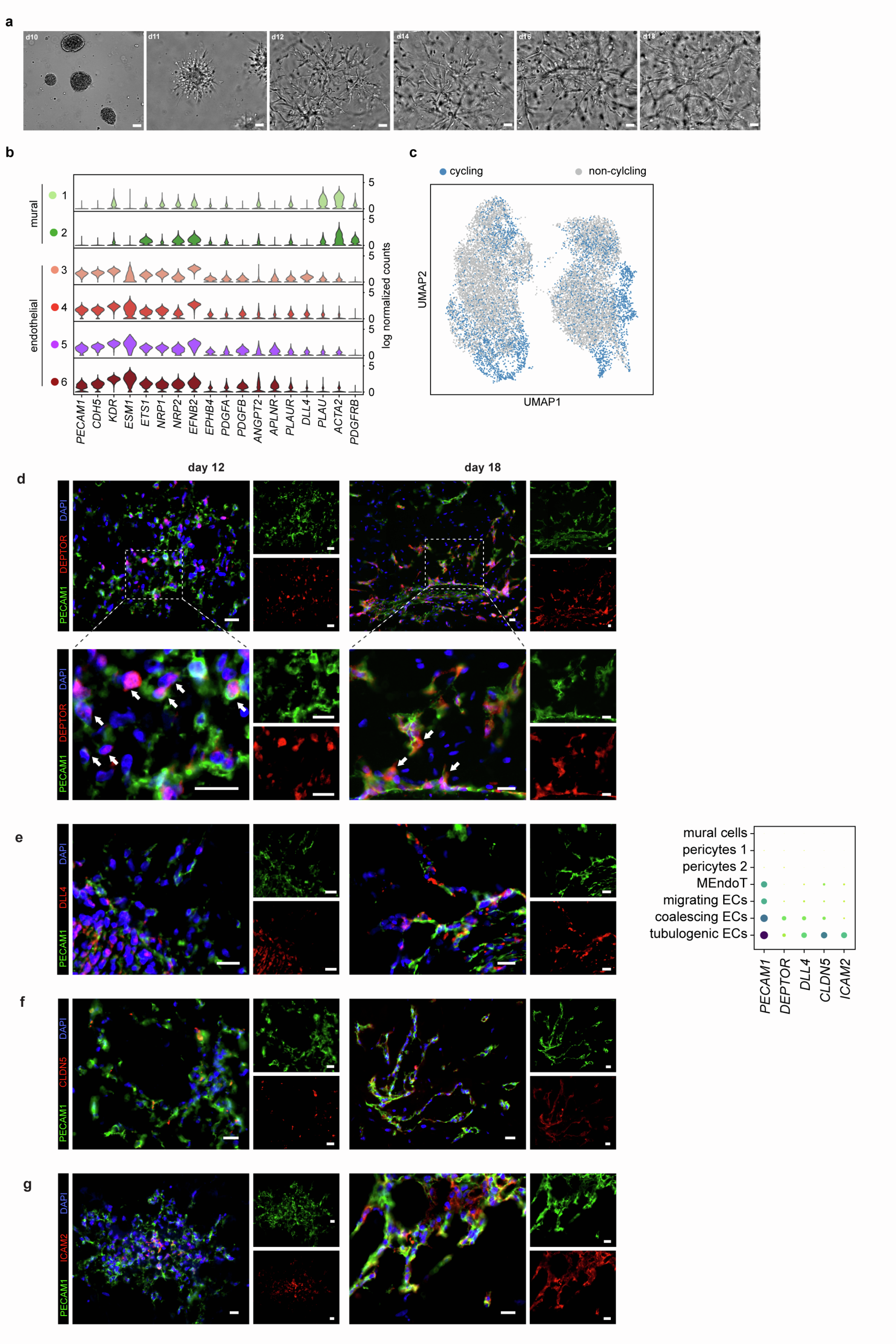

a
d10
d11
d12
d14
d16
d18
b
mural
endothelial
1
2
3
4
5
6
log normalized counts
PECAM1
CDH5
KDR
ESM1
ETS1
NRP1
NRP2
EFNB2
EPHB4
PDGFA
PDGFB
ANGPT2
APLNR
PLAUR
DLL4
PLAU
ACTA2
PDGFRB
c
cycling
non-cylcling
UMAP2
UMAP1
d
day 12
day 18
DAPI
DEPTOR
PECAM1
e
DLL4
mural cells
pericytes 1
pericytes 2
MEndoT
migrating ECs
coalescing ECs
tubulogenic ECs
CLDN5
ICAM2
f
g

Figure S3. Single-cell transcriptomes and immunofluorescence (IF) analysis of ECs and mural cells cultured in Matrigel. **a**, Bright-field images of the Matrigel culture along the timeline of days 10 to 18. Scale: 50 µm. **b**, Violin plot of representative marker genes used to assign the cell clusters in the UMAP plot in main Fig 4. The density distribution indicates the normalized cluster mean expression. **c**, UMAP plot shows the cells with gene expression relating to S, G2, or M-phase (blue). Cells expressing genes indicative of the G1 phase are denoted with a grey color. IF stainings show the protein signal of the differentially expressed genes indicative of the migrating (**d**, **e**), coalescing, and tubulogenic ECs (**f-g**). Arrows in **d**, day 12 (lower left panel) highlight the location of DEPTOR in the nucleus, while on day 18 (lower right panel) DEPTOR is expressed in the nucleus and cytoplasm. Scale: 20 µm. **h**, Dot plot of mRNA expression of genes whose corresponding protein staining is shown above. The color intensity and dot size denote the normalized cluster mean expression and the fraction of cells expressing the gene, respectively. *n*=4 images have been acquired for each condition. The figure corresponds to the main **Figure 4 and 5**.

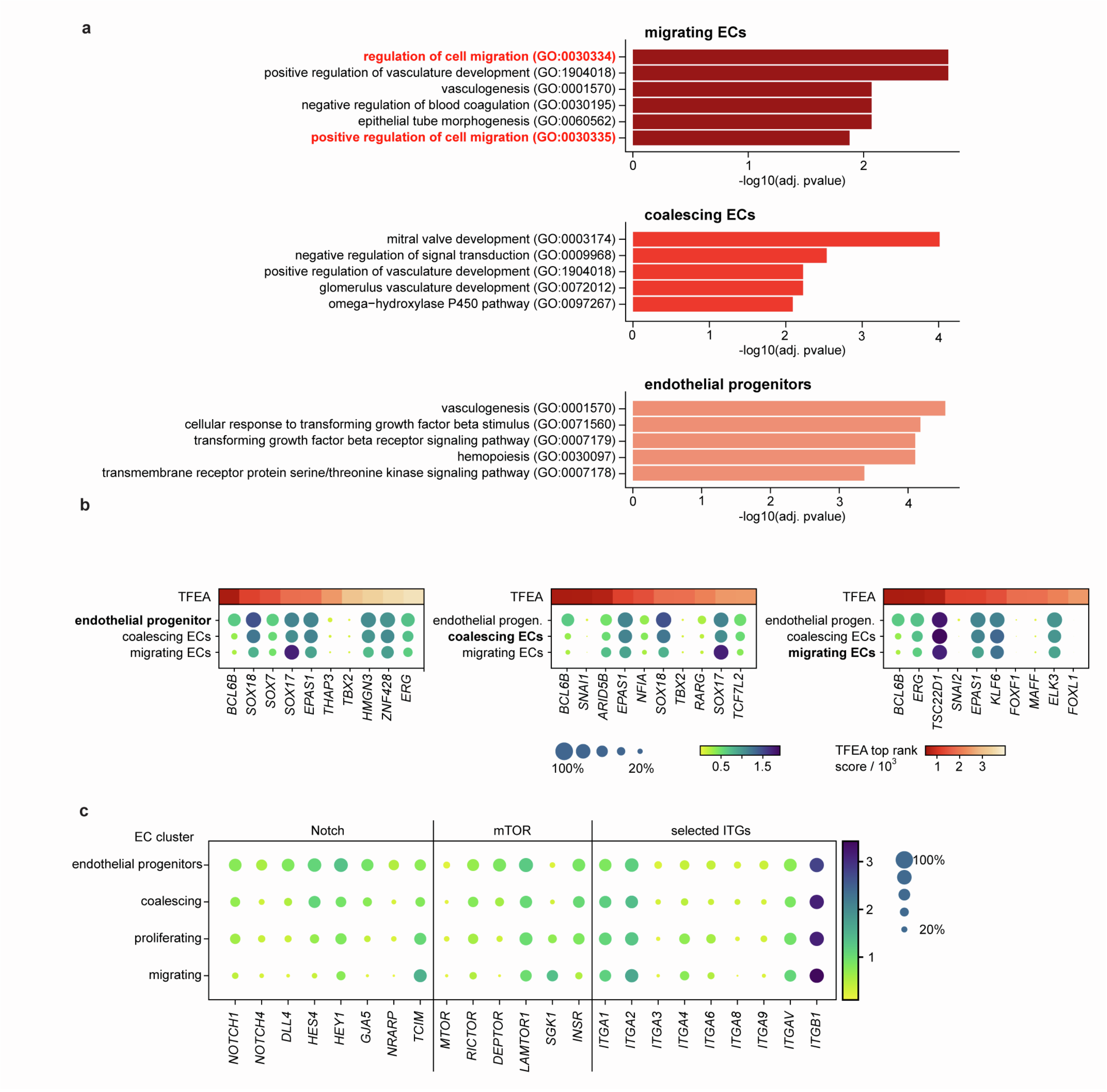

Figure S4. Signal pathway analysis during neovascularization at day 12 of differentiation in Matrigel. **a**, Gene ontology term analysis based on DEGs from the sc-transcriptomes of endothelial progenitor cells, coalescing and migrating ECs. **b**, Transcription factor enrichment analysis based DEGs the endothelial progenitor cells (left), coalescing (middle), and migrating (right) EC cluster. DEGs between the EC clusters were filtered for expression and standard deviation before TFEA. TFEA scores are represented in a color code, whereas the mean expression levels of the corresponding TFs as dot plots. **c**, Dot plot of expression of genes within the mTOR and Notch pathway regulated during the cell state transition from endothelial progenitor cells to coalescing ECs and sprouting ECs. The color intensity and dot size denote the normalized cluster mean expression and the fraction of cells expressing the gene, respectively. The figure corresponds to the main **Figure 4**.

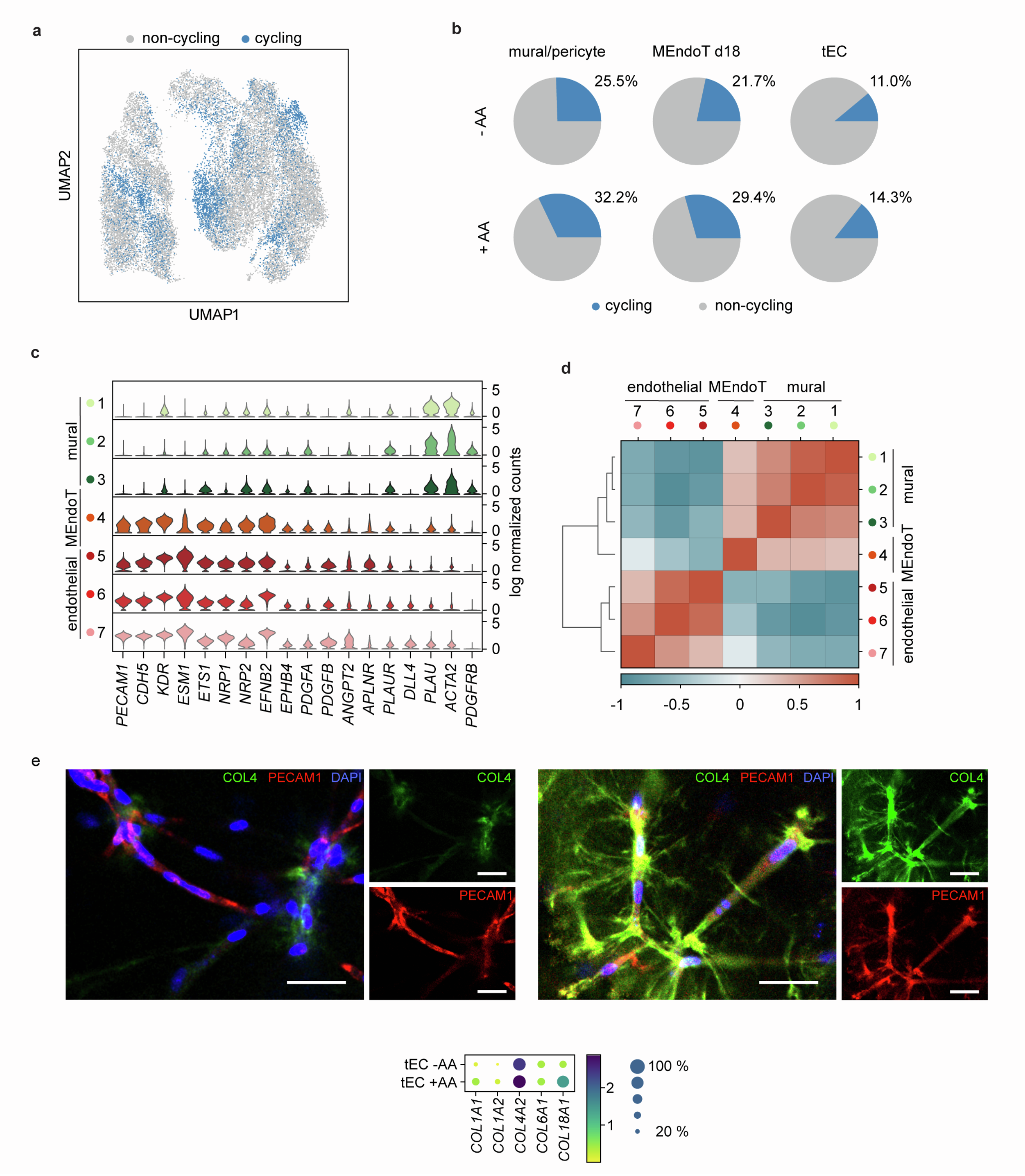

Figure S5. Single-cell transcriptomes and immunofluorescence analysis of stem-cell derived endothelial cells forms microvessels in Matrigel culture on day 18 of differentiation with and without ascorbic acid. a, UMAP plot of scRNA-transcriptomes acquired from cells in the Matrigel cultures on days 12 and 18. Blue colored dots represent cells expressing genes relating to S, G2, or M-phase. **b**, Pie charts show the percentage of cells undergoing the cell cycle within the respective clusters in the presence and

absence of ascorbic acid (AA). **c**, Violin plot of marker genes used to assign the cell cluster. The density distribution indicates the normalized cluster mean expression. **d**, Cell cluster Pearson correlation plot. **e, Left:** IF image of Matrigel culture on day 18 without and **right**: with AA addition to the media, where COL4, DAPI, and PECAM1 were counterstained. Scale: 20 µm. Dot plot of single-cell profile of selected collagen genes with ECM functionality in EC cultures with and without ascorbic acid. The color intensity and dot size denote the normalized cluster mean expression level and the fraction of cell expression for the corresponding gene, respectively. The figure corresponds to the main **Figure 5**.

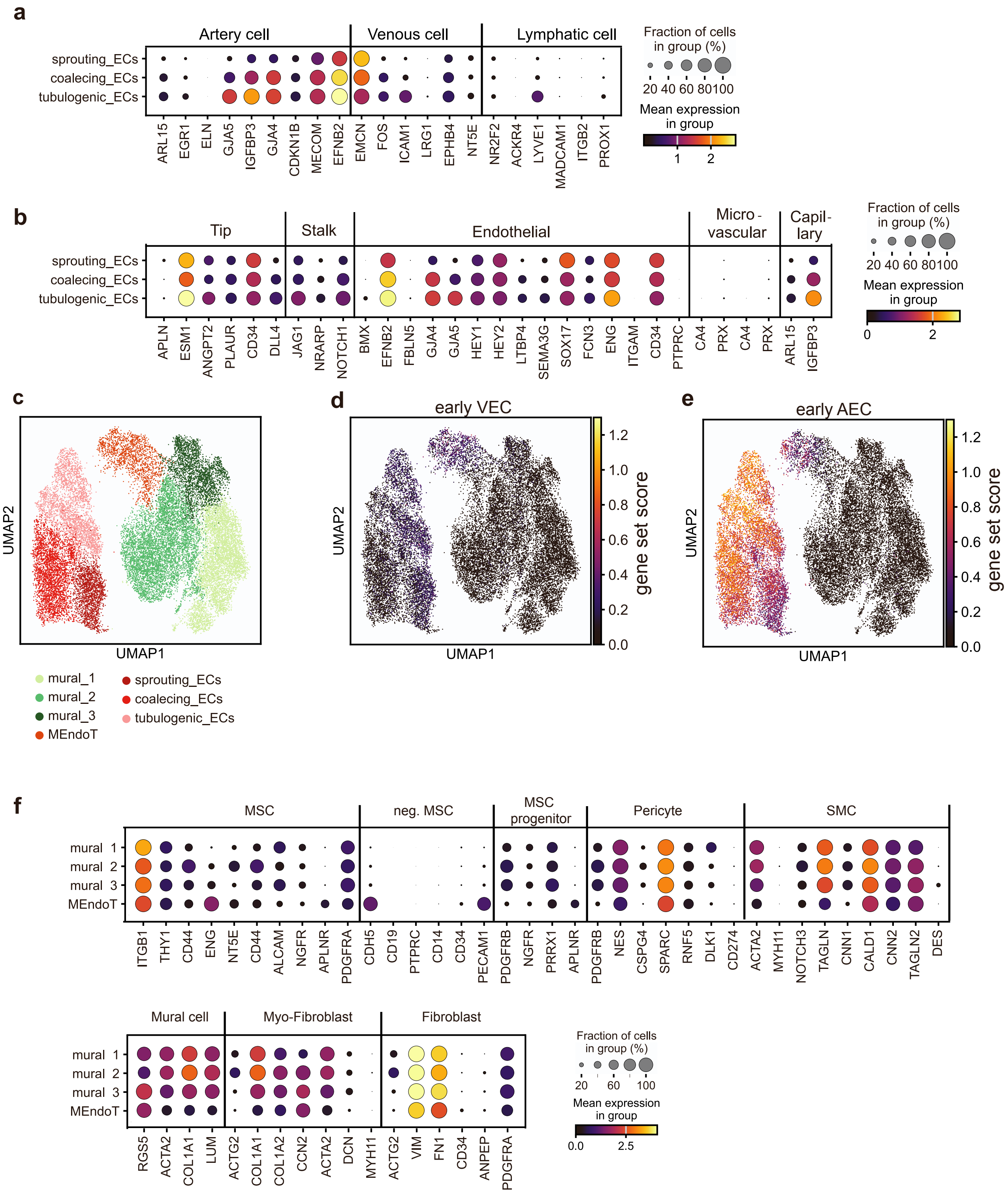

Figure 6. Standard cell type marker within the singe-cell transcriptomes of day 12 and 18 stem cell-derived hydrogel culture. a and **b**, Dot plots of mRNA expressions of literature curated cell type markers

in cells of the ECs clusters. **c**, UMAP plot of the single-cell transcriptomes as shown in Figure 5b of the main text. **d**, and **e**, Gene set enrichment analysis of the top 300 DEGs from single cell transcriptomes of early at the arterial, and venous ECs (Carnegie stage 10 and 11) within the cell clusters shown in **c**. Dot plots of mRNA expressions of literature curated cell type markers in the cells of the mural clusters. The figure corresponds to the main **Figure 5.**

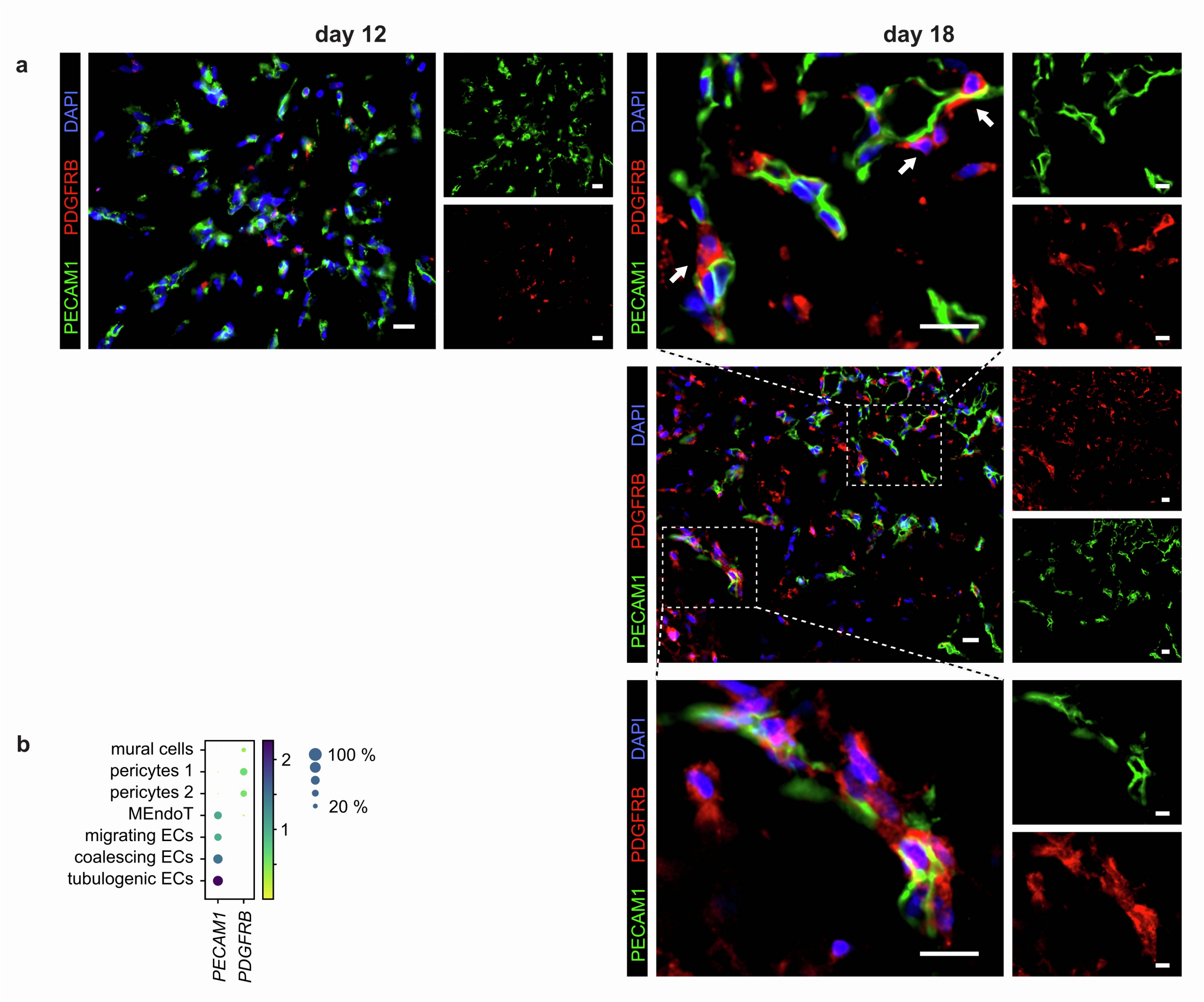

Figure S7. IF images of cryo-sections prepared from 3D hydrogel cultures on days 12 and 18 of differentiation. a, Location of mural cells to ECs is shown upon co-staining of PDGFRB and PECAM1, respectively. On day 18 (right panel) pericyte recruitment is highlighted by arrows. Scale: 20 µm. **b**, Dot plot of mRNA expression of genes whose corresponding protein staining is shown above. The color intensity and dot size denote the normalized cluster mean expression and the fraction of cells expressing the gene, respectively. *n*=4 images have been acquired for each condition. The figure corresponds to the main **Figure 6**.

Supplemental Tables

Table S1. Cell sample and sequencing statistics.

sample ID	condition	percentage of dead cells pre sequencing	total cell numbers pre quality filtering	mean number of genes per cell	mean number of sequencing read counts	number of analyzed cells after quality filtering	cell numbers with more than 10% mitochondrial genes	cell numbers with less than 2000 genes
day 0	3D suspension culture	17.4	8554	4440	22361	6411	811	1332
day 3	3D suspension culture	36.5	9621	4132	19660	4549	490	4582
day 6	3D suspension culture	19.1	6428	3644	12080	4807	345	1276
day 9_1	3D suspension culture	25	8154	4051	15200	6425	211	1517
day 9_2	3D suspension culture	30.8	7010	4388	19199	3011	804	3193
day 12	3D suspension culture	16.1	10551	5354	24817	4114	3161	3276
day 12	3D Matrigel	4.3	16097	3835	12165	13530	689	1876
day 18	3D Matrigel	12.3	11777	4807	19542	6462	2268	3047
day 18	3D Matrigel + ascorbic acid	7.6	14135	4673	19369	8743	2620	2772

The table corresponds to the main **Figure 1**.

Table S2 Culturing conditions of the differentiation in 2D and 3D.

	day of differentiation		this study	McCracken et al.	day of differentiation
**lateral mesoderm induction**	0-3	media	N2B27	N2B27	1-4
		BMP4	25 ng/mL	25 ng/mL	
		CHIR99021	7.5 µM	7 µM	
**endothelial induction**	3-7	media	StemPro-34	StemPro-34	4
		VEGF-A	200 ng/ml	200 ng/ml	
		Forskolin	2 µM	2 µM	
**maturation stage**	from 8 on	media	StemPro-34	EGM-2	until 8
		VEGF-A	30 ng/ml	50 ng/ml	
		FGF-2	30 ng/ml		
		human AB serum		1%	

The table corresponds to the main **Figure 1**.

Table S3. Cell type composition of the two culturing conditions on day 12 and day 18 (corresponds to Figure 4c and Figure 5c)**.**

**Day 12**	**d12 suspension**	**d12 MTG**	**d12 suspension / %**	**d12 MTG / %**
mural_1	1120	5339	34.7	47.2
mural_2	612	904	19	8
Endothelial progenitor	1175	42	36.4	0.4
coalescing_EC	221	2631	6.9	23.3
proliferating_EC	66	422	2	3.7
migrating_EC	30	1966	0.9	17.4

**Day 18**	**d12 MTG**	**d18 -AA**	**d18+AA**	**d12 MTG / %**	**d18 -AA / %**	**d18+AA / %**
mural_1	4950	197	104	44.3	3.5	1.4
mural_2/P1	198	1762	4528	1.8	31.2	59.8
mural_3/P2	980	816	949	8.8	14.4	12.5
MEndoT	13	779	850	0.1	13.8	11.2
migrating_ECs	1789	107	5	16	1.9	0.1
coalescing_ECs	3218	188	17	28.8	3.3	0.2
tubulogenic_ECs	31	1805	1116	0.3	31.9	14.7

The table corresponds to the main **Figure 5**.

Supplemental Experimental Procedures

Sample preparation for scRNA-seq

Matrix-free samples were washed with PBS, resuspended in Accutase, and incubated for cell detachment at 4°C for 30 min. The reaction was stopped by adding five volumes of media. Afterward, cells were washed once with PBS. Matrigel embedded samples were washed with PBS and incubated with 1 mg/mL Collagenase/Dispase solution (Sigma-Aldrich, Cat#10269638001) in StemPro-34 for around 4 h, until organoids detached from the Matrigel. The enzymatic reaction was stopped with 1 mL neutralisation buffer comprising 1%BSA, 1%P/S in DMEM:F12 (Sigma Aldrich, Cat#D6421). The single cells were cryo-preserved in DMEM with 10% heat-inactivated FBS (Thermo Fisher Scientific, Cat#A3160401) and 10% DMSO based on a previously described scRNA-seq sample preparation protocol (Guillaumet-Adkins et al., 2017). For sequencing, cryo-preserved cells were thawed in DMEM:F12. RNA libraries were generated using Chromium Single Cell 3' library and gel bead kit v3.1 (10x Genomics, Cat#1000079). The amplified cDNA library was sequenced on a NovaSeq 6000 S2 flow cell (Illumina, Cat#20028314). The sequenced cell numbers can be found in **Table S1**.

ScRNA-seq data pre-processing

Sequencing raw files were demultiplexed, aligned (reference genome hg38_ensrel97), filtered, barcodes and UMIs counted, and subjected to a quality filter with CellRanger (version 3.0.1, 10xGenomics). The pre-processing and downstream analysis was performed with the package 'Scanpy API' in python with default parameters, if not stated differently (Wolf et al., 2018). First, dead or stressed cells, identified by a percentage of mitochondrial genes higher than 10%, were filtered out. Next, cells with less than 200 and genes expressed in less than three cells were excluded. Afterward, the datasets of different days and experiments were concatenated, normalized to 10^4 gene counts per cell, and log-transformed. Batch effects were corrected using ComBat (Johnson et al., 2007). Further on, the highly variable genes were used for the downstream analysis. We corrected for the total gene counts, percentage of mitochondrial genes, and the cell cycle distribution of S, G2, and M phases to investigate differentiation-dependent changes on the transcriptome level (Luecken and Theis, 2019).

Dimensionality reduction, clustering, and cell-type annotation

The single cell nearest neighborhood graph was computed with the first 50 principal components and ten nearest neighbors. The cells were clustered with the Leiden algorithm with a resolution of 0.5. For visualization, the dimensionality of the data was reduced using Uniform Manifold Approximation and Projection (UMAP). For cell-type annotation, 300 DEGs for each of the clusters were calculated by ranking the clusters against all remaining cells with the t-test method (**Data File 1**). Clusters with proteasome-related genes scored at the top or a significantly reduced gene count were removed from the dataset as representing dying or damaged cells. The remaining clusters were annotated based on known marker genes.

RNA velocity through dynamical modeling

We analyzed the RNA velocity to investigate developmental trajectories by recovering directed dynamic gene information through splicing kinetics. Information like clustering and UMAP coordinates were retrieved from the Scanpy analysis. The pre-processing and downstream analysis was performed with scVelo using default parameters (Bergen et al., 2020). Splice variants and cells were filtered, normalized, and logarithmized with the function scv.pp.filter_and_normalize (parameters: min_cells=3, min_counts=200, min_shared_counts=20, n_top_genes=2000). The moments based on the connectivities were calculated with 40 PCAs and 10 neighbors in the next step. After recovering the dynamics, the latent time was calculated, and the velocity was calculated as a dynamical model.

Integration of datasets from different sequencing approaches
For integration and correction of datasets from different sequencing runs (**Figure 3**, **Figure S3c** and **d**), we applied bbknn to the datasets (neighbors_within_batch=40, n_pcs=10, trim=0, copy=True). We then re-clustered the cells with the Leiden algorithm at a resolution of 0.5 (Polański et al., 2020; Traag et al., 2018)).

Transcription factor enrichment analysis
The identified cluster-specific DDGs or DEGs (**Data File 1**) were entered in the ChEA3 web tool (Keenan et al., 2019) and the mean rank was plotted using R.

Enriched gene expression of gene sets

For the gene enrichment UMAP plots from Figure 1f, we used the data from Tyser et al. and converted the raw data to anndata file using scanpy. Cells were normalized to 10000 reads per cell and logarithmized. For enrichment scoring we used the scanpy command sc.tl.score_genes. The score function subtracts the average expression of a set of genes (top 300 DEGs of the clusters) with a reference gene set expression, randomly sampled from the whole gene pool.

Pathway and Gene Ontology enrichment
DEGs were filtered by their unique expression over all clusters (standard deviation above 0.5) and an expression value above 0.5. For the GO term enrichment, the R package enrichR was used with the "GO Biological Process 2018" database and plotted in R (Chen et al., 2013; Kuleshov et al., 2016).

CellphoneDB
The count matrix and cluster annotation were exported from scanpy, imported into R, and processed as recommended by the authors (Efremova et al., 2020). Cell-cell interactions were selected by the highest mean score and lowest p-value.

NicheNet
As Target gene input, the top 300 DEGs have been used. The calculation was done in R, converting the anndata element into a Seurat object. The process was performed as recommended by the authors (Browaeys et al., 2020).

Software specifications
The scRNA-seq alignment was run in CellRanger version 3.0.1 and the analyses were run in python 3.7.4 with Scanpy API version 1.4.4 or 1.5.1, anndata version 0.6.22 or 0.7.6, umap version 0.4.6, numpy version 1.17.4, scipy version 1.5.2, pandas version 1.1.3 or 1.0.5, scikit-learn version 0.22, statsmodels version 0.10.1, python-igraph version 0.7.1, scvelo version 0.2.1, matplotlib version 3.2.1, seaborn version 0.9.0, loompy version 3.0.6, XlsxWriter version 1.2.6, bbknn version 1.3.6 and scrublet version 0.2.1.

The plots of TFEA and GO term analysis were generated in RStudio with R version 3.6.0 with the usage of the R packages enrichR_3.0, ggpubr_0.4.0, ggplot2_3.3.3, stringr_1.4.0, EBImage_4.32.0, and bioimagetools_1.1.5.

NicheNet analysis was performed using following package versions: xlsx_0.6.5, ggpubr_0.4.0.999, cowplot_1.1.1, RColorBrewer_1.1-2, circlize_0.4.13, forcats_0.5.1, stringr_1.4.0, dplyr_1.0.7, purrr_0.3.4, readr_2.1.1, tidyr_1.1.4, tibble_3.1.0, ggplot2_3.3.5, tidyverse_1.3.1, SeuratObject_4.0.4, Seurat_4.0.2, nichenetr_1.0.0, gridBase_0.4-7, and ComplexHeatmap_2.6.2

Fluorescence-activated cell sorting (FACS)
Harvested cells were washed three times with PBS (200 x g for 5 minutes) and singularized using Accutase. Five volumes of FACS buffer (10% FBS in PBS) were added and centrifuged at 300 x g for 5 minutes. Blocking was done for 20 minutes with StemPro-34 plus 10 % FBS on ice. The cell suspension was filtered using a 40 µm nylon cell strainer (Corning, Cat#431750). Live dead staining was performed with Trypan

Blue (Fisher Scientific, Cat#15393661) while cells were counted. 20 µL of the antibody FITC anti-Human CD31 (BD Pharmingen, Cat#555445) and PE anti-Human CD140b (BD Pharmingen, Cat#558821), and 1 µL of violet, fluorescent reactive dye (Invitrogen, Cat# L34963A) were added per 10^6 cells and incubated for 30 minutes at room temperature (RT). After washing once with the FACS buffer, cells were resuspended in Tyto buffer (10^6 cells/mL, MACSQuant Tyto (TM) Running Buffer (Miltenyi Biotech, Cat#130-107-207). The sorting was performed on a MACSQuant Tyto. For isotype controls the FITC mouse IgG1 κ isotype control (BD Pharmingen, Cat#554679) and PE mouse IgG2a κ Isotype Control (BD Pharmingen, Cat#559319) were used.

Flow cytometry
Singularized cells were washed once with PBS (centrifugation conditions: 300 x g, 5 min). Cells were fixated at RT for 15 minutes with 4% PFA (LifeTechnologies, Cat#28908) in PBS and afterward washed twice with FACS buffer. 10^5 cells were transferred into a U-bottom-shaped 96-well plate (Greiner, 650101) and an antibody (2 µL per 15^5 cells) diluted in 100 µL FACS buffer was applied per well. After incubation for 30 minutes at RT, cells were washed twice with FACS buffer. For the measurement, the pellet was resuspended in 200 µL of FACS buffer and transferred through a cell strainer into 5mL round-bottom FACS-tubes (Coring, Cat#352052). Flow cytometry analysis was performed on a MACSQuant VYB.

Cryo-embedding
For embedding of 3D suspension culture aggregates, 500 µL of 4% PFA were added and incubated for 15 minutes on ice. The disc was detached from the walls with a needle. After two washing steps with PBS, an incubation with, first, 10%, second, 30% sucrose at RT for 2 h and, third, with a 1:1 mixture of 30% sucrose and OCT medium (Carl Roth, 6478.1) at 4°C overnight. All incubation steps were implemented on a wave shaker at RT. The medium was replaced by pure OCT and the sample was frozen on dry ice. The slicing was performed on a Leica CM1860 cryostat, where the thickness was adjusted to 20 µm. We used the following antibodies to prepare the immunofluorescence stainings: PECAM1 (Thermofisher, Cat#AbWM59), PDGFRB (Thermofisher, Cat#AbAPB5), DLL4 (Cell Signaling, Cat#mAb 96406), DEPTOR (Cell Signaling, Cat#mAB 11816), CLDN5 (Thermofisher, Cat#MA5-32614), ICAM2 (Cell Signaling, Cat#mAB 13355), Collagen IV (Abcam, Cat#ab6586), anti-mouse Alexa 488 (Thermofisher, Cat#A21202), and anti-rabbit Alexa 555 (Invitrogen, Cat#A31572).

Cell migration assay

A fluorescently labeled iPSC line (Corell Institute, Cat# AICS-0036-028) was differentiated according to the above reported 3D differentiation protocol. On day 6 of differentiation, endothelial progenitors were FACS sorted for $PECAM1^+$ (Becton Dickinson, Cat#555455) and then cultivated in EGM2 media (Angio-Proteomie, Cat#cAP-02) with supplements (EGM2) in 6-wells following the given procedure for 2D cell cultures. Sorted endothelial progenitors were two times passaged before use. The cell migration assay was set up on a commercially available microfluidic chip from Mimetas (Mimetas, OrganoPlate 3-lane 40 Cat#4004-400-B). For handling the chip platform, we followed the vendor's instructions. The chip comprises three converging microfluidic channels with inlet and outlet ports. Upon filling the middle channel with a 2.5 mg/ml neutralized Collagen-1 (Corning, Cat#354236) hydrogel solution solved in PBS, the two outer channels were separated by a diffusion barrier. After gelation at 37°C for 30 min the inlet port of the gel chamber was filled with Hanks' Balanced Salt solution (Gibco, Cat#14175095) to prevent drying out of the collagen solution. Then, the two outer microchannels were filled with EGM2 supplemented with half the volume of a given growth factor supplement mix solution (EGM2-s/2) to reduce the internal growth factor contribution to the assay. For cell seeding on the chip, the endothelial progenitors were retrieved from the cell culture plate with Accutase (Sigma Aldrich, Cat#A6964) treatment for 5 minutes. After two times washing SC-ECs with EGM2-s/2, the cells were pipette into one of the microchannel inlet ports with a

concentration of 15.000 cells/ml. 30 minutes after seeding the inlet ports were filled up with 50 µL EGM2-s/2 and placed on the rock shaker (tilt angle 7° with an inverting interval of 8 minutes) to induce flow conditions. After SC-EC reached confluency between 30% and below 80% within the microfluidic channel the ligands were added to the inlet port of the microchannel opposing the microchannel with the cells. The cell confluency was measured as described below. Fluorescence images of the hydrogel chamber were taken before and 24h after ligand addition. All ligands had a concentration of 10ng/mL, except for angiopoietin and CRX12 which had a concentration of 100ng/mL. The increase of the cell confluency within the hydrogel area or seeding channel of the chip platform was obtained by first cropping the same region of interest (ROI) from all fluorescence images. Cropped images were converted into binary images by using a constant global threshold value. Cell occupancy areas in the binary images were determined with the measure function of ImageJ.

References

Bergen, V., Lange, M., Peidli, S., Wolf, F.A., and Theis, F.J. (2020). Generalizing RNA velocity to transient cell states through dynamical modeling. Nature Biotechnology 2020 38:12 *38*, 1408–1414. https://doi.org/10.1038/s41587-020-0591-3.

Browaeys, R., Saelens, W., and Saeys, Y. (2020). NicheNet: modeling intercellular communication by linking ligands to target genes. Nature Methods *17*, 159–162. https://doi.org/10.1038/s41592-019-0667-5.

Chen, E.Y., Tan, C.M., Kou, Y., Duan, Q., Wang, Z., Meirelles, G.V., Clark, N.R., and Ma'ayan, A. (2013). Enrichr: Interactive and collaborative HTML5 gene list enrichment analysis tool. BMC Bioinformatics *14*, 128. https://doi.org/10.1186/1471-2105-14-128.

Efremova, M., Vento-Tormo, M., Teichmann, S.A., and Vento-Tormo, R. (2020). CellPhoneDB: inferring cell–cell communication from combined expression of multi-subunit ligand–receptor complexes. Nature Protocols *15*, 1484–1506. https://doi.org/10.1038/s41596-020-0292-x.

Johnson, W.E., Li, C., and Rabinovic, A. (2007). Adjusting batch effects in microarray expression data using empirical Bayes methods. Biostatistics *8*, 118–127. https://doi.org/10.1093/biostatistics/kxj037.

Keenan, A.B., Torre, D., Lachmann, A., Leong, A.K., Wojciechowicz, M.L., Utti, V., Jagodnik, K.M., Kropiwnicki, E., Wang, Z., and Ma'ayan, A. (2019). ChEA3: transcription factor enrichment analysis by orthogonal omics integration. Nucleic Acids Research *47*, W212–W224. https://doi.org/10.1093/nar/gkz446.

Kuleshov, M.V., Jones, M.R., Rouillard, A.D., Fernandez, N.F., Duan, Q., Wang, Z., Koplev, S., Jenkins, S.L., Jagodnik, K.M., Lachmann, A., et al. (2016). Enrichr: a comprehensive gene set enrichment analysis web server 2016 update. Nucleic Acids Research *44*, W90–W97. https://doi.org/10.1093/nar/gkw377.

Luecken, M.D., and Theis, F.J. (2019). Current best practices in single-cell RNA-seq analysis: a tutorial. Molecular Systems Biology *15.* https://doi.org/10.15252/msb.20188746.

Polański, K., Young, M.D., Miao, Z., Meyer, K.B., Teichmann, S.A., and Park, J.E. (2020). BBKNN: fast batch alignment of single cell transcriptomes. Bioinformatics *36*, 964–965. https://doi.org/10.1093/bioinformatics/btz625.

Traag, V.A., Waltman, L., and Eck, N.J. van (2018). From Louvain to Leiden: guaranteeing well-connected communities. Sci. Reports *9*, 5233. https://doi.org/10.1038/s41598-019-41695-z.

Wolf, F.A., Angerer, P., and Theis, F.J. (2018). SCANPY: Large-scale single-cell gene expression data analysis. Genome Biology *19*, 15. https://doi.org/10.1186/s13059-017-1382-0.
